# gPKPDSim: a SimBiology^®^-based GUI application for PKPD modeling in drug development

**DOI:** 10.1007/s10928-017-9562-9

**Published:** 2018-01-04

**Authors:** Iraj Hosseini, Anita Gajjala, Daniela Bumbaca Yadav, Siddharth Sukumaran, Saroja Ramanujan, Ricardo Paxson, Kapil Gadkar

**Affiliations:** 1 Genentech Inc., South San Francisco, CA USA; 2 MathWorks Inc., Consulting Services, Natick, MA USA; 3 MathWorks Inc., Natick, MA USA

**Keywords:** Modeling and simulation, Graphical-user-interface, Non-compartmental analysis, Pharmacokinetics and pharmacodynamics models

## Abstract

Modeling and simulation (M&S) is increasingly used in drug development to characterize pharmacokinetic-pharmacodynamic (PKPD) relationships and support various efforts such as target feasibility assessment, molecule selection, human PK projection, and preclinical and clinical dose and schedule determination. While model development typically require mathematical modeling expertise, model exploration and simulations could in many cases be performed by scientists in various disciplines to support the design, analysis and interpretation of experimental studies. To this end, we have developed a versatile graphical user interface (GUI) application to enable easy use of any model constructed in SimBiology^®^ to execute various common PKPD analyses. The MATLAB^®^-based GUI application, called gPKPDSim, has a single screen interface and provides functionalities including simulation, data fitting (parameter estimation), population simulation (exploring the impact of parameter variability on the outputs of interest), and non-compartmental PK analysis. Further, gPKPDSim is a user-friendly tool with capabilities including interactive visualization, exporting of results and generation of presentation-ready figures. gPKPDSim was designed primarily for use in preclinical and translational drug development, although broader applications exist. gPKPDSim is a MATLAB^®^-based open-source application and is publicly available to download from MATLAB^®^ Central™. We illustrate the use and features of gPKPDSim using multiple PKPD models to demonstrate the wide applications of this tool in pharmaceutical sciences. Overall, gPKPDSim provides an integrated, multi-purpose user-friendly GUI application to enable efficient use of PKPD models by scientists from various disciplines, regardless of their modeling expertise.

**Electronic supplementary material:**

The online version of this article (10.1007/s10928-017-9562-9) contains supplementary material, which is available to authorized users.

## Introduction

Characterization of drug pharmacokinetics (PK) and its relationship with pharmacodynamic (PD), efficacy and safety endpoints are critical in pharmaceutical research and development and are used to inform efforts such as target and molecule selection, human PK projection, and clinical dose and schedule determination. Quantitative analysis and modeling and simulation (M&S) tools are frequently employed to characterize and predict PKPD relationships, understand drug mechanism of action, support optimal design of studies and reduce animal usage in preclinical studies, and guide clinical translation and clinical study design. Commonly used M&S approaches include non-compartmental analysis and compartmental PK models with empirical or mechanistic pharmacodynamics (PD) and toxicology and/or efficacy models. Further, exploring the variability within these models is a critical component of model-based analyses.

Typically, these tasks are performed by scientists with training and expertise in PKPD modeling and simulation. However, while model development might require such expertise, certain model-based exploration and applications could be conducted by scientists from other disciplines (e.g., pharmacology, toxicology, biomarker discovery & development, bioanalytical sciences, and clinical sciences) if provided with validated models in user-friendly, graphical-user-interface (GUI) based tools. These scientists could ideally directly utilize such modeling tools in their study design, data analysis, interpretation and decision-makings.

A variety of software and applications are available to perform PKPD analysis, modeling, and simulation. Some of the most commonly used PKPD modeling tools include Phoenix^®^ WinNonlin^®^, NONMEM^®^, Adapt V, SAAM II, R and MATLAB^®^ SimBiology^®^. These modeling tools can be script-based or have a graphical user interface to help construct and simulate models. Pharmacometricians and PKPD M&S scientists are typically trained in one or more of these tools. Although some of the tools are more user-friendly than others, these are rarely used by non-specialists even when the mathematical models are already available. Hence a simpler multi-purpose tool with a user-friendly interface can promote the broader use of PKPD models in various stages of drug development, by scientists from diverse disciplines.

Wojciechowski et al. [1] have developed an interactive application using Shiny for the R programming language that can help with sharing and communication of pharmacometric model results. Shiny allows for customization of the application’s user-interface to provide an environment for displaying user-input controls and simulation output that can be simultaneously updated with changing input. Ermakov et al. [2] have developed the Virtual Systems Pharmacology software platform for simulations of models in a software free environment. The platform-enabled configuration of models developed in any software of choice for simulations on a web based interface with a back-end database capability. The platform, however, is not publicly available. Germani et al. [3] introduced the A4S Simulator, developed in MATLAB^®^, that uses a library of models and provides different dosing options; however, features such as non-compartmental analysis, interactive visualization and project management are limited in this program. Krause et al. [4] demonstrated the visualization of pharmacometric models using the Berkeley Madonna software with a particular focus on interactive sessions to facilitate communications with non-modelers.

We have developed a GUI-based application called gPKPDSim (Genentech PKPD Simulator) to broaden utilization of preclinical and translational PKPD models in drug development. The tool is made available with this manuscript and allows deployment of models developed by expert modelers to a broader group of scientists and researchers for hands-on use. Any model developed within SimBiology^®^ can be configured into gPKPDSim. The application and features of gPKPDSim are demonstrated using a library of standard PKPD models for typical model-related drug development tasks.

## Methods

### Overview of software development

The gPKPDSim application was developed using MATLAB^®^, SimBiology^®^ and the Statistics and Machine Learning Toolbox and the user requires a MATLAB^®^ license with the mentioned toolboxes to be able to launch the application. If the Optimization or Global Optimization Toolbox is installed, gPKPDSim can also use the parameter estimation methods from either of those toolboxes. The default choice for the optimization method is *nlinfit* (which is suitable for nonlinear least-square problems), however, the modeler can change the optimization method when the Session file is configured (see Supplementary Method S1 for how to create a session file)

The implementation of the application is based on the model-view-controller or MVC architecture. The “model” contains the analysis or configuration. This can be executed from the MATLAB^®^ command-line independently from the front-end viewer. The model is represented by the back-end objects with the “+PKPD” MATLAB^®^ package folder. The “viewer” designates the graphical user interface (GUI), which is mostly contained within the “+PKPDViewer” package. Implementation of the viewer is based on the “GUI Layout Toolbox”, a programmatic layout manager, from MATLAB^®^ Central^™^. The “controller”, represented by the viewer’s callback functions, responds to inputs by the user by updating the model and subsequently the viewer. The controller is also contained with the “+PKPDViewer” package folder.

The application has been tested in MATLAB^®^ R2016a and R2016b. The software is not compatible with earlier MATLAB^®^ releases and currently has not been tested for releases after MATLAB^®^ R2016b. The application requires MATLAB^®^ and is *not* supported for distribution via MATLAB^®^ Compiler. Both Windows and Mac operating systems are supported. The software is available as Supplementary Material and on MATLAB^®^ Central™ (The gPKPDSim and NCA toolboxes are available at: https://www.mathworks.com/matlabcentral/fileexchange/65399-gpkpdsimtoolbox and https://www.mathworks.com/matlabcentral/fileexchange/65303-simbiologynca)

### Library of models

**Fig. 1:**
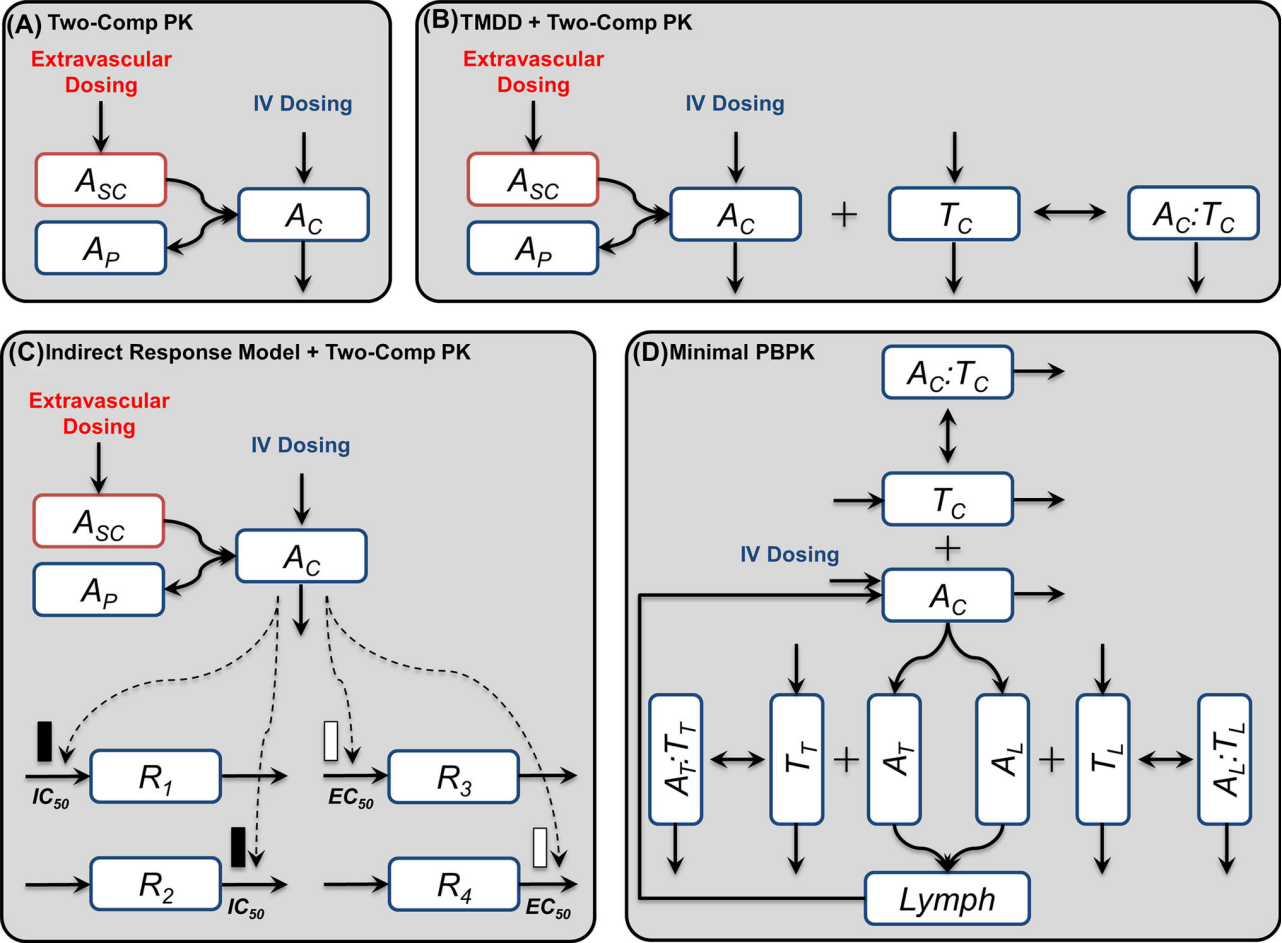
A schematic diagram of the PKPD models in the library. The models include **a** two-compartment PK model with specific and non-specific clearance, **b** target-mediated drug disposition (TMDD) model, **c** physiologic indirect response model, and **d** minimal physiologically based pharmacokinetic (PBPK) model. Symbols *A*, *T* and *A:T* denote the antibody, target and the antibody:target complex. Subscripts indicate the different compartments in each model (*C*: central, *P*: peripheral, *SC*: subcutaneous or (extravascular), *T*: tight tissues, *L*: leaky tissues). In the indirect response model, solid and open symbols represent inhibition and stimulation

With this manuscript, we have provided a set of PKPD models that are frequently used in the pharmaceutical industry; however, any other model built in SimBiology^®^ can be easily configured for gPKPDSim. The models in our library include (1) two-compartment pharmacokinetics model with IV and extravascular dosing. The model includes both non-specific clearance (typically captured by a linear term) and specific clearance (typically captured by a nonlinear Michaelis–Menten term to model target-mediated drug disposition). (2) Target-mediated drug disposition (TMDD) model [5], (3) physiologic indirect response models (with inhibitory or stimulatory effects on synthesis or degradation of some endogenous target or mediator) [6] and (4) minimal physiologically based pharmacokinetic (PBPK) model with target represented in the central compartment, leaky and tight tissues [7]. Each SimBiology^®^ model is encapsulated in a “Session” file, that can be loaded in gPKPDSim by end-users for performing PKPD tasks. The schematic diagram of each model in the library is shown in Fig. 1.

### GUI application overview

**Fig. 2:**
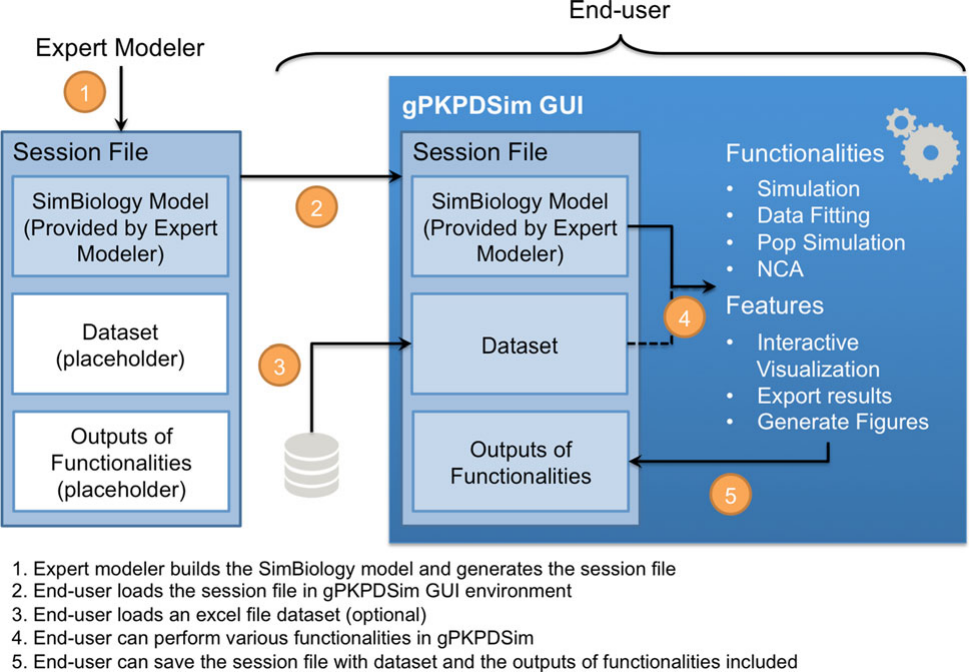
A schematic workflow of how modelers and end-users employ gPKPDSim. The expert modeler develops the model in SimBiology^®^ and encapsulates it into a “Session” file, which can be launched in gPKPDSim by end-users. The Session file, once loaded in gPKPDSim, enables the end-user to perform different functionalities, including simulation, data fitting (parameter estimation), population simulation, and NCA. For some of these functionalities, the user must provide a dataset. All the functionalities are supported by features such as interactive visualization, and export of results as presentation-ready figures and Excel datasets. When the user completed their tasks, the session file can be saved for future use. Note that the end-user works exclusively in the environment of gPKPDSim

In the framework developed in this work, model development performed by the expert modeler is separated from utilization of the model, a task that can be performed by a broader range of scientists and researchers. Thus, using the framework shown schematically in Fig. 2, standard PKPD models can be developed once by the expert and used repeatedly by end-users in diverse projects.

**Fig. 3:**
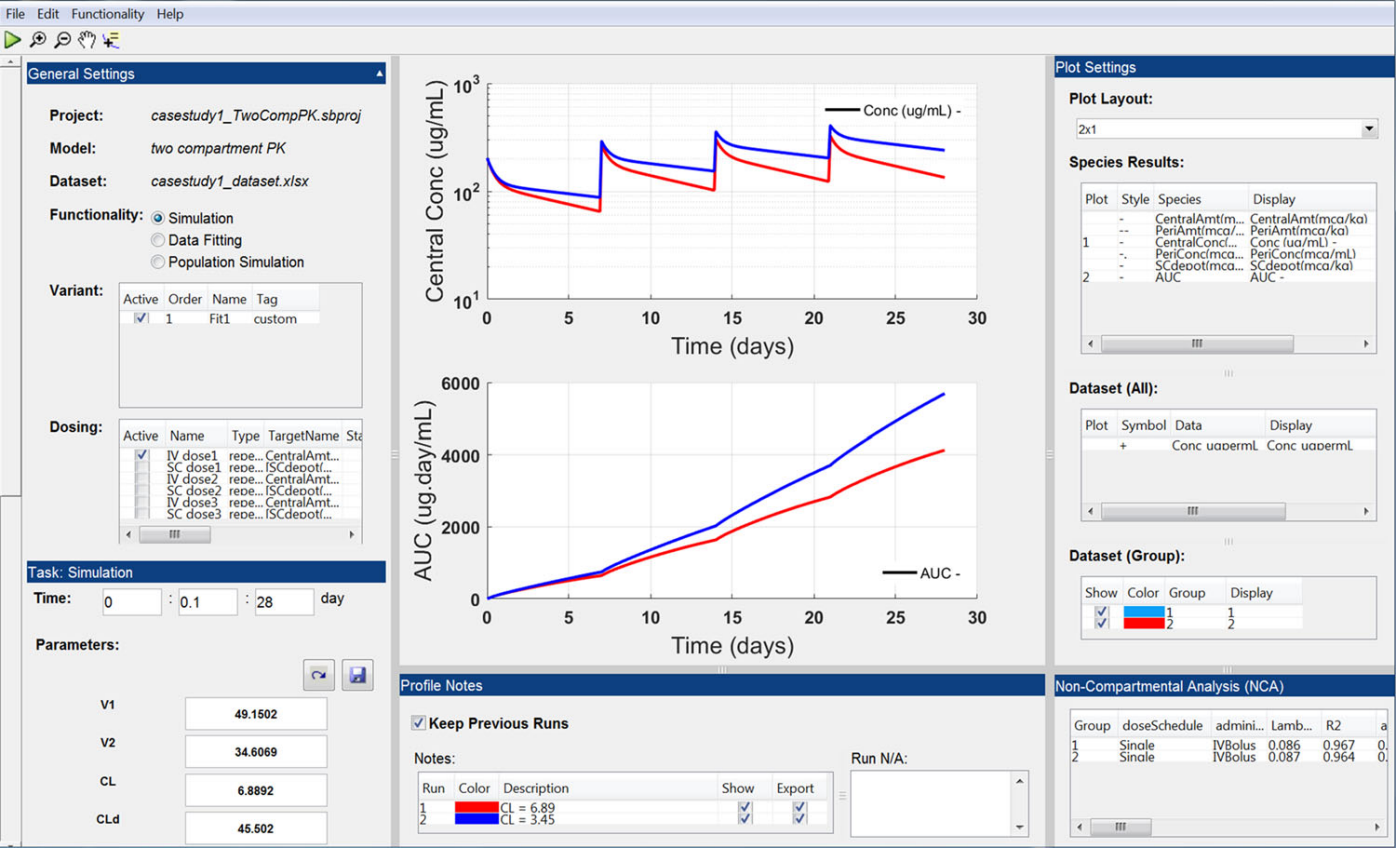
gPKPDSim: simulation functionality view. This view has multiple sections: *1*) general settings (top left); *2*) functionality-specific settings including simulation time and parameter values (bottom left); *3*) plot settings including species from the SimBiology^®^ model and dataset groups and column headers (top right); *4*) non-compartmental analysis (NCA, bottom right); *5*) profile notes including a list of most recent simulations and their detailed summary (bottom middle); and *6*) interactive plots (top middle). See “Methods” for detailed description of each section

The expert modeler builds the model in SimBiology^®^ and configures it into a “Session” file, which contains specification of the SimBiology^®^ model Variants, Doses, Species, Parameters and Settings for use within the user interface (see Supplementary Method S1 for how to create a session file). The Session file, once loaded in gPKPDSim, enables functionalities that include (1) simulation, (2) data fitting (parameter estimation), (3) population simulation, and (4) NCA. All these functionalities are supported by some common features such as interactive visualization, export of results as presentation-ready figures and Excel datasets, and saving the working Session files for future use. The end-user works exclusively in the environment of gPKPDSim and can send the saved session files to communicate with collaborators and share the results. Table 1 shows the description of different components of the gPKPDSim framework, and Fig. 3 shows a snapshot of gPKPDSim GUI.

**Table 1:** Description of the components within the gPKPDSim framework

Components	Description
SimBiology^®^ Project	A SimBiology^®^.sbproj file containing the PKPD model of interest (this is created by the modeler)
gPKPDSim GUI	Main application for end-users to interact with the SimBiology^®^ model
Session File	A MATLAB^®^.mat session file created for a SimBiology^®^ project. The file is generated from configuration.m (Supplementary Method S1), which determines how the model is loaded into gPKPDSim. Once launched in gPKPDSim, the end-user can store a dataset and the outputs of functionalities in the session file
Dataset File	A preclinical/clinical dataset to be used for visualization, NCA or data fitting (see Supplementary Method S2 for the dataset formats)
Functionality	Different functionalities within the application: 1. Simulation 2. Data fitting (parameter estimation) 3. Population simulation 4. NCA
Feature	Common features including interactive visualizations, exporting results as figures and datasets, and saving working session files support all the functionalities

### gPKPDSim general settings

This section includes the common settings between the different functionalities. Table 2 lists a summary of the general settings and menus in the GUI. A more detailed description of variants and dosing are provided below (see Supplementary Method S3 for more details on the remaining gPKPDSim settings).

**Table 2:** A summary of different settings in gPKPDSim

General settings	Description
Project	SimBiology^®^ project file within the session file that is open
Model	SimBiology^®^ model used
Dataset	Imported data file
Functionality	Radio button selection for functionality
Variant	List of SimBiology^®^ parameter variants
Dosing	Dosing options with dosing information including start time, dose amount, number of repeats and repeat interval
*Menus*	
File menu	Provides options for opening a session, saving current session, importing a dataset and exporting results for the output of each functionality
Edit menu	Contains property inspector for modifying plot properties
Functionality menu	Allows user to run or perform a functionality such as simulation, fitting, or population simulation

#### Variant

Displays a list of built-in variants from the SimBiology^®^ model. A variant stores the names and values of parameters of the SimBiology model and allows the user to use values stored in a variant object as the alternate value to be applied during a simulation. For example, if you have different values for PK parameters in animals and humans, those can be differentiated through variants (see Fig. S1 for a visual example and https://www.mathworks.com/help/simbio/ref/variantobject.html for more information). Each session file can select from the variants available in the SimBiology^®^ sbproj file; not all variants from the sbproj file need to be included in the session file. If a single variant is activated, the values of the parameters in gPKPDSim will be overwritten by the values stored in the variant. If multiple variants are activated, the variants will overwrite the parameter values in an ascending order. The user then can change parameter values in the functionality-specific section after activating the variants. At any point, the user can reset the value to default by clicking “Reset to defaults” button. To summarize, here is the order in which the parameter values are assigned:

1. Default parameter values from the session file.
2. Activated variants change the values in ascending order.
3. The user can then manually overwrite parameter values.

#### Dosing

Displays a list of dosing objects available to the user. Each row contains dosing information that can be edited by the user. This includes the start time, amount, interval, rate and repeat-count (note that only SimBiology^®^ “repeat dose” dose objects will be shown in the table. See https://www.mathworks.com/help/simbio/ref/repeatdoseobject.html for more information). For example, if there is an experiment with weekly dosing at 1000 μg/kg at Day 0, 7, 14 and 21, this can be defined as a dose object with the following values (amount = 1000 μg/kg, start time = 0, interval = 7 days, and repeat-count = 3). The user can select as many doses as required to build the desired dosing regimen. The dosing table is only utilized for the simulation and population simulation functionalities (for fitting tasks the dose information is obtained from the data file).

### Functionalities

#### Simulation

Enables simulation of the model encapsulated in the session file with different parameter values, variants, dosing regimens and simulation time. Once the simulation is completed, the user can visualize results using the interactive plotting features. The user can choose to run multiple simulations and retain the results of previous simulations for the purpose of visualization or results export to Excel files. The profile notes section of gPKPDSim includes a detailed summary of each simulation run. In addition to simulation, the user can also import data for visualization and comparison with the simulation results (Supplementary Method S3).

#### Non-compartmental analysis (NCA)

Estimate the PK parameters for the imported dataset. The dataset can include any combination of dosing route (IV and/or extravascular dosing), and schedule (single-dose and/or multi-dose scenarios). gPKPDSim can compute NCA for sparse (a PK sampling scheme in which not all subjects were sampled at all the time points) and serial (a PK sampling scheme in which all subjects were sampled at all the time points) schemes with automatic detection of the dosing values from the dataset (see Supplementary Method S2 for the dataset formats). It also allows the user to provide an array of time ranges to estimate C_max_ and partial AUC values. For calculating the terminal half-life, the NCA method has two options: (1) the “Automatic” option for which, the NCA method automatically determines the time points used in estimating the half-life and (2) the “Custom” option for which the user must provide a time range [t_0_ t_N_], which determines the time points to be included in the estimation of terminal half-life (Supplementary Method S3). The NCA outputs and their definitions are as follows:

- *Group*: Shows the identifier for the Group column header in the dataset. As mentioned before, a Group is defined as a number of Subjects receiving the same dose material, through the same dose route, and according to the same dose schedule.
- *Dose schedule*: Frequency of administration. This could be single or multiple.
- *Administration route*: Intravenous or extravascular administration.
- *Lambda_Z*: First-order rate constant of elimination (commonly referred to as the terminal slope).
- *R2*: A coefficient of determination that is a statistical measure of how well the regression line for half-life determination approximates the real data points.
- *adjusted_R2*: A modified version of R2 that has been adjusted for the number of points being included in the regression line for half-life determination.
- *Num_points*: Number of points included in the regression line for half-life determination.
- *AUC_0_last*: Area under the concentration time curve from time zero to the last measured time point.
- *Tlast*: The last measured time point.
- *C_max*: The maximum concentration following the first dose and before the second dose.
- *C_max_Dose*: C_max_ normalized by the dose level.
- *T_max*: The time at which C_max_ was assessed.
- *MRT*: Mean residence time; the average amount of time the drug remains in a compartment.
- *T_half*: Terminal half-life.
- *AUC_infinity*: Area under the concentration time curve from time zero and extrapolated to infinity.
- *AUC_infinity_dose*: AUC_infinity normalized by the dose level.
- *AUC_extrap_percent*: Percentage of the AUC_infinity value that is from extrapolation.
- *CL*: Clearance
- *DM*: Dose level
- *V_z*: Volume of distribution of the terminal phase.
- *AUMC_0_last*: Area under the moment curve from time zero to the last measured time point.
- *AUMC*: Area under the moment curve from time zero and extrapolated to infinity.
- *AUMC_extrap_percent*: Percentage of the AUMC_infinity value that is from extrapolation.
- *V_ss*: Volume of distribution at steady state.
- *C_0*: Concentration at time zero.
- *AUC_x__y*: Area under the concentration time curve from time x to time y.
- *C_max_x__y*: The maximum concentration between time x and time y.
- *T_max_x__y*: Time at which the maximum concentration between time x and time y occurred.
- *C_avg*: Average concentration at steady state.
- *PTF_Percent*: Percent the concentrations fluctuate between Cmax and Cmin at steady state.
- *Accumulation_Index*: Estimation of drug accumulation at steady state.
- *AUC_Tau*: Area under the concentration time curve at steady state for the dose interval.
- *AUMC_Tau*: Area under the moment curve at steady state for the dose interval.
- *T*_*lag*_: Lag time between the time of dosing and the time of first appearance of measurable concentrations.

#### Data fitting (parameter estimation)

This functionality allows the end-user to estimate model parameter values, providing a best fit of model simulation to data. The user imports the dataset in gPKPDSim and maps all the data outputs to be used in data fitting as well as the dosing information for each group of subjects. The user can also select the error model for the objective function, specify if pooled data should be used to perform the data fitting and select parameters to be estimated. After the data fitting algorithm is complete, the user can save estimated parameter values as a new variant (which will appear in the list of variants and the tag column will show “Custom”) within the current session, enabling use of the parameter estimates for subsequent simulation or population simulation functionality (Supplementary Method S3). Note that the data fitting functionality in gPKPDSim does not provide parameter estimation for mixed-effect models, which is available in other software such as SimBiology^®^, NONMEM^®^ and Monolix.

#### Population simulation

The purpose of this functionality is to explore the impact of parameter variability on the model outputs of interest. The user specifies the distribution of parameters to be used; this can be based on expert knowledge or based on the standard errors in parameter estimates from the fitting task. Note that the population simulation functionality is not based on parameter distributions estimated from mixed-effect models, but relies on the end-user to provide the variability of each parameter. And example of how covariates are handled in gPKPDSim is provided in Supplementary Method S3. The user also specifies the number of simulations, i.e., the number of times the parameter distributions are sampled and the model is run. Once the population simulation is complete, the visualization plots display the median curve as well as a shaded region corresponding to the 5–95% boundaries for the output selected. Similar to other functionalities, the user can save the plots and/or export the results to an Excel file.

## Results

### Case Study #1: NCA, two-compartment model fit, and multi-dose projection for PK of a large molecule

A common scenario encountered in pharmaceutical R&D is the use of data obtained with one dose regimen to characterize PK and to then project PK for other dosing scenarios. In this example, gPKPDSim is used to perform NCA [8] for PK data for single dose administration of a monoclonal antibody [9], followed by fitting of the data to a two-compartment PK model, which is then used to simulate and predict multi-dose PK and population variability. Thus, this example uses NCA, fitting, simulation, and population simulation functionalities of gPKPDSim. The instructions in Supplementary Instruction S1 can be followed for working through this exercise (see Table S5 for initial conditions). The dataset used in the analyses is provided in the supplement and includes systemic PK measurements for the study design in Table 3.

**Table 3:** Study design for a single-dose IV administration (Case Study #1)

Group no.	No. of subjects	Admin. route & dose schedule	Dose level (mg/kg)	Duration (days)
1	3	Single-dose IV	10	35
2	3	Single-dose IV	100	35

NCA outputs are calculated for two scenarios: (1) sparse (or group-level) analysis resulting in two sets of NCA parameters, one for each dosing group (Table 4) and (2) serial (or sub-group-level) analysis resulting in six sets of NCA parameters, one for each animal and (Table 5). The NCA results are computed to NCA results estimated by WinNonlin^®^ for both cases (Table 4 and Supplementary Table S3). We also included thirteen additional datasets for different dosing regimens including IV and extravascular, single and multi-dose scenarios (see Table S4 and Supplementary Files). In all cases, the absolute difference between the NCA results of gPKPDSim and those of WinNonlin^®^ was less than 0.1%, when the number of data points used for calculation of parameters are matched. As seen in Table 5, the estimated value of clearance (CL) varies between 6.01 and 8.02 mL/kg/day across animals and the range for estimated volume of distribution (V_z) is 74.49–81.84 mL/kg. Next, we use the data fitting functionality to fit the PK data to a two-compartment model to estimate the PK parameters. The standard two-compartment antibody PK model with non-specific (captured by a linear term) and specific (captured by a nonlinear term) clearance used here is described by the following set of differential equations:

1 $$\frac{{d A_{C} }}{dt} = k_{abs} \cdot f_{bio} \cdot A_{SC} - CL \cdot X_{C} - \frac{{V_{m} \cdot X_{C} }}{{K_{m} + X_{C} }} - CL_{d} \left( {X_{C} - X_{P} } \right),$$

2 $$\frac{{d A_{P} }}{dt} = CL_{d} \cdot \left( {X_{C} - X_{P} } \right),$$

3 $$\frac{{d A_{SC} }}{dt} = - k_{abs} \cdot A_{SC} ,$$

4 $$\frac{d AUC}{dt} = X_{C} ,$$

5 $$X_{C} = \frac{{A_{C} }}{{V_{1} }},$$

6 $$X_{P} = \frac{{A_{P} }}{{V_{2} }},$$

where *A*_*C*_, *A*_*P*_, and *A*_*SC*_ represent the amount of drug in the central, peripheral, and extravascular (subcutaneous) compartments; *X*_*C*_ and *X*_*P*_ denote the drug concentration in the central and peripheral compartments; and *AUC* represents the area under the curve for *X*_*C*_. The units for antibody amounts and concentrations are μg/kg and μg/mL. Parameters *V*_1_ and *V*_2_ represent the volume of central and peripheral compartments, *CL* represents the clearance from the central compartment, *CL*_*d*_ denotes the distribution clearance between the central and peripheral compartments, *k*_*abs*_ represents the absorption rate from the extravascular compartment and *f*_*bio*_ is the bioavailability, that is the fraction of drug available in the central compartment after extravascular dosing (see Supplementary File casestudy1_TwoCompPK_equations.pdf for the units of parameters and species in the model).

**Table 4:** NCA results for the sparse sampling scheme from gPKPDSim and WinNonlin^®^ (two sets of NCA parameters corresponding to two groups)

Group	gPKPDSim		WinNonlin^®^	
	1	2	1	2
Dose schedule	Single	Single	Single	Single
Administration route	IV Bolus	IV Bolus	IV Bolus	IV Bolus
Lambda_Z (1/day)	0.086	0.087	0.086	0.087
R2	0.967	0.964	0.967	0.964
adjusted_R2	0.963	0.959	0.963	0.959
Num_points	9	9	9	9
AUC_0_last (day μg/mL)	1389.4	14,209.2	1389.4	14,209.2
Tlast (day)	35	35	35	35
C_max (μg/mL)	194.6	2080.0	194.6	2080.0
C_max_Dose (kg μg/mL/μg)	19.4	20.8	19.4	20.8
T_max (day)	0.01047	0.01047	0.01047	0.01047
MRT (day)	11.4	11.3	11.4	11.3
T_half (day)	8.1	8.0	8.1	8.0
AUC_infinity (day μg/mL)	1449.4	14,772.5	1449.4	14,772.5
AUC_infinity_dose (day kg μg/mL/μg)	145.0	147.7	144.9	147.7
AUC_extrap_percent (%)	4.1	3.8	4.1	3.8
CL (mL/day/kg)	6.9	6.8	6.9	6.8
DM (μg/kg)	10,000	100,000	10,000	100,000
V_z (mL/kg)	80.5	77.9	80.5	77.9
AUMC_0_last (day day μg/mL)	13,785.0	140,560.9	13,785.3	140,561.2
AUMC (day day μg/mL)	16,584.7	166,756.3	16,585.6	166,757.2
AUMC_extrap_percent (%)	16.9	15.7	16.9	15.7
V_ss (mL/kg)	79.0	76.4	79.0	76.4
C_0 (μg/mL)	196.1	2099.4	196.1	2099.4

**Table 5:** NCA results for the serial sampling scheme from gPKPDSim (two groups × three animals = six sets of NCA parameters)

Group	gPKPDSim					
	1	1	1	2	2	2
Dose schedule	Single	Single	Single	Single	Single	Single
Administration route	IV Bolus	IV Bolus	IV Bolus	IV Bolus	IV Bolus	IV Bolus
Animal_ID	A	B	C	D	E	F
Lambda_Z (1/day)	0.085	0.076	0.102	0.087	0.077	0.101
R2	0.956	0.963	0.980	0.964	0.945	0.979
adjusted_R2	0.950	0.957	0.977	0.959	0.937	0.976
Num_points	9	9	9	9	9	9
AUC_0_last (day μg/mL)	1384.6	1565.6	1217.9	14,012.4	15,592.0	13,023.1
Tlast (day)	35	35	35	35	35	35
C_max (μg/mL)	191.3	205.7	186.7	2051.2	2145.9	2042.8
C_max_Dose (kg μg/mL/μg)	19.1	20.6	18.7	20.5	21.4	20.4
T_max (day)	0.01047	0.01047	0.01047	0.01047	0.01047	0.01047
MRT (day)	11.6	12.8	9.7	11.3	12.7	9.8
T_half (day)	8.2	9.1	6.8	8.0	9.0	6.9
AUC_infinity (day μg/mL)	1443.7	1663.4	1246.9	14,585.4	16,448.2	13,330.6
AUC_infinity_dose (day kg μg/mL/μg)	144.4	166.3	124.7	145.9	164.5	133.3
AUC_extrap_percent (%)	4.1	5.9	2.3	3.9	5.2	2.3
CL (mL/day/kg)	6.9	6.0	8.0	6.9	6.1	7.5
DM (μg/kg)	10,000	10,000	10,000	100,000	100,000	100,000
V_z (mL/kg)	81.8	78.7	79.0	78.9	78.7	74.5
AUMC_0_last (day day μg/mL)	13,984.1	16,579.6	10,791.1	137,861.6	167,597.7	116,223.5
AUMC (day day μg/mL)	16,747.8	21,283.6	12,092.7	164,509.2	208,640.7	130,038.2
AUMC_extrap_percent (%)	16.5	22.1	10.8	16.2	19.7	10.6
V_ss (mL/kg)	80.4	76.9	77.8	77.3	77.1	73.2
C_0 (μg/mL)	192.8	207.6	188.1	2068.8	2166.5	2063.1

**Fig. 4:**
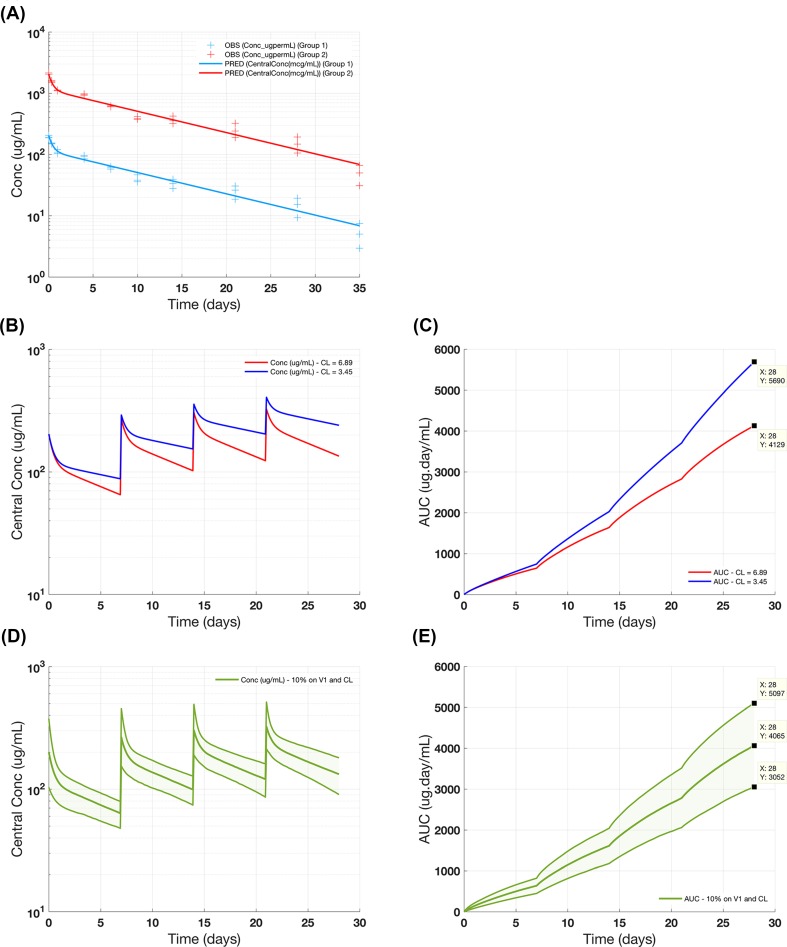
Case Study #1: two-compartment PK model for antibody. The results show **a** the goodness of fit for the model fitted to data for the low and high dose groups; **b** and **c** simulated PK profiles and AUC curves, for two different CL values at 10 mg/kg; and **d** and **e** population simulation for exploring the impact of variability on PK and AUC (median and 5–95% bounds)

The quality of the parameter estimation can be evaluated by the value of standard error on the fitted parameter values and the visual predictive checks, included in the Fitting Summary report (see Supplementary File casestudy1_fitting_summary_combined_pooled.pdf). For this dataset, the non-linear clearance term is not required to explain the data, as the drug exposure is dose-proportional for the low and high dose groups. The results for the pooled fitting are shown in Fig. 4a. The parameter estimates for the pooled and group-specific fittings, shown in Table 6, have a relatively low standard error. The estimated value of CL is 6.89 mL/Kg/day, which is consistent with the NCA results. The central and peripheral compartment volumes are estimated at 49.1 and 34.6 mL/Kg, respectively. Note that the performance of data fitting in gPKPDSim is essentially that of “Fit Data” task in SimBiology^®^.

**Table 6:** Parameter estimates (± standard error) for the pooled and group-specific fittings

Name	Initial	Pooled fitting	Group-specific fitting		Units
		Fit—Group	Fit—Group 1	Fit—Group 2	
*V*_1_	40	49.1 ± 3.8	50.7 ± 1.4	47.3 ± 1.0	mL/kg
*V*_2_	40	34.6 ± 5.2	31.0 ± 3.9	32.8 ± 3.4	mL/kg
*CL*	5	6.9 ± 0.2	7.2 ± 0.3	7.1 ± 0.3	mL/kg/day
*CLd*	10	45.5 ± 17.4	45.7 ± 9.4	50.1 ± 7.8	mL/kg/day

Finally, we use the model and parameter estimates together to project PK for an alternate dosing regimen and explore potential PK variability. The parameter estimates from the pooled-fitting were saved in the form of a “variant”, and then used for projecting the PK profile of a 10 mg/kg 4qw (weekly dosing for 4 weeks) IV dosing regimen under different conditions listed in Table 7 to explore the effect of the PK parameters on drug exposures; this was done using the simulation and population simulation functionalities. The PK profiles are shown in Fig. 4b and d and the AUC curves are depicted in Fig. 4c and e (AUC_d0-28_ for the three scenarios are reported in Table 7). The simulation results suggest that decreasing the clearance by half results in a 38% increase in AUC for this molecule. Additionally, assuming 10% population variability on *V*_1_, and *CL* results in a median AUC value of 4065 μg × day/mL with the 5–95% boundaries being 25% lower or higher than the median.

**Table 7:** Estimated AUC values for the period of 0–28 days of a 10 mg/kg 4qw IV dosing regimen under different conditions (Fig. 4c and e)

gPKPDSim functionality	Scenario	AUC_d0–28_ (μg day/mL)
Simulation	*CL*_1_ = 6.89 (result of fitting)	4129
Simulation	*CL*_2_ = 0.5 × *CL*_1_ = 3.45	5690
Population simulation	*CL*3 = 6.89 + 10% CV *V*_1_ = 49.14 + 10% CV	Median = 4065 5–95% = 3052–5097

### Case Study #2: target-mediated drug disposition model to predict antibody PK and target engagement

In addition to characterizing, fitting, and projecting PK as in example 1, antibody drug development often involves projecting target occupancy as a function of dose regimen and antibody affinity, in order to determine the antibody affinity needed or identify the dosing requirements for desired target engagement. In this case study, we consider a two-compartment model with target mediated antibody drug disposition in the central compartment [5]. This model is an extended version of the two-compartment model, in which the non-linear Michaelis–Menten clearance term is replaced with target mediated drug disposition. The model equations are shown below:

7 $$\frac{{d A_{C, free} }}{dt} = k_{abs} \cdot f_{bio} \cdot A_{SC, free} - CL \cdot X_{C, free} - CL_{d} \left( {X_{C, free} - X_{P, free} } \right) - \left( {k_{on} \cdot X_{{C, free\left( {nM} \right)}} \cdot T_{{C, free\left( {nM} \right)}} - k_{on} \cdot K_{D} \cdot C_{{C\left( {nM} \right)}} } \right) \cdot \frac{{MW_{Ab} }}{1000} \cdot V_{1} ,$$

8 $$\frac{{d A_{P, free} }}{dt} = CL_{d} *\left( {X_{C, free} - X_{P, free} } \right),$$

9 $$\frac{{d A_{SC, free} }}{dt} = - k_{abs} \cdot A_{SC, free} ,$$

10 $$\frac{{d T_{C, free(nM)} }}{dt} = \frac{{{ \ln }(2)}}{{t_{1/2, T} }}T_{{C, init\left( {nM} \right)}} - \frac{{{ \ln }(2)}}{{t_{1/2, T} }}T_{{C, free\left( {nM} \right)}} - \left( {k_{on} \cdot X_{{C, free\left( {nM} \right)}} \cdot T_{{C, free\left( {nM} \right)}} - k_{on} \cdot K_{D} \cdot C_{{C\left( {nM} \right)}} } \right),$$

11 $$\frac{{d C_{C(nM)} }}{dt} = \left( {k_{on} \cdot X_{{C, free\left( {nM} \right)}} \cdot T_{{C, free\left( {nM} \right)}} - k_{on} \cdot K_{D} \cdot C_{{C\left( {nM} \right)}} } \right) - \alpha_{CL} \frac{CL}{{V_{1} }} \cdot C_{{C\left( {nM} \right)}} ,$$

12 $$X_{C, free} = \frac{{A_{C, free} }}{{V_{1} }},$$

13 $$X_{P, free} = \frac{{A_{P, free} }}{{V_{2} }},$$

14 $$X_{C, free(nM)} = X_{C, free} \frac{1000}{{MW_{Ab} }},$$

15 $$X_{C, total} = \left( {X_{{C, free\left( {nM} \right)}} + C_{{C\left( {nM} \right)}} } \right)\frac{{MW_{Ab} }}{1000},$$

16 $$T_{C, free} = T_{C, free(nM)} \cdot MW_{T} ,$$

17 $$T_{C, total} = \left( {T_{{C, free\left( {nM} \right)}} + C_{{C\left( {nM} \right)}} } \right) \cdot MW_{T} ,$$

18 $$f_{T, bound} = \hbox{min} \left( {1, { \hbox{max} }\left( {0, 1 - \frac{{T_{C, free(nM)} }}{{T_{{C, init\left( {nM} \right)}} }}} \right)} \right),$$

where *A*_*C, free*_, *A*_*P, free*_, and *A*_*SC, free*_ represent the amount of free drug in the central, peripheral, and SC compartments; the concentration of free target and the drug:target complex is represented by *T*_*C, free(nM)*_ and *C*_*c(nM)*_; the concentration of free drug in the central and peripheral compartments is denoted by *X*_*C, free*_ and *X*_*P, free*_. Unless otherwise noted, the units for antibody amounts, antibody concentrations, and target concentrations are μg/Kg, μg/mL, and ng/mL. Compared to the model presented in Case Study #1, this model has additional parameters including *k*_*on*_, *K*_*D*_, *MW*_*Ab*_, *MW*_*T*_, *t*_1*/*2*,T*_, *T*_*c,init(nM)*_, and *α*_*CL*_ representing the drug-to-target association rate constant, equilibrium dissociation constant, molecular weight of drug, molecular weight of target, half-life of target, initial concentration of target, and the ratio of the clearance of the bound drug-target complex to free drug clearance. In this model the target is considered to be a soluble target present in circulation. The stoichiometry of binding is taken to be 1:1 for antibody and target to form the complex (see Supplementary File casestudy2_TMDD_equations.pdf for the units of parameters and species in the model).

**Fig. 5:**
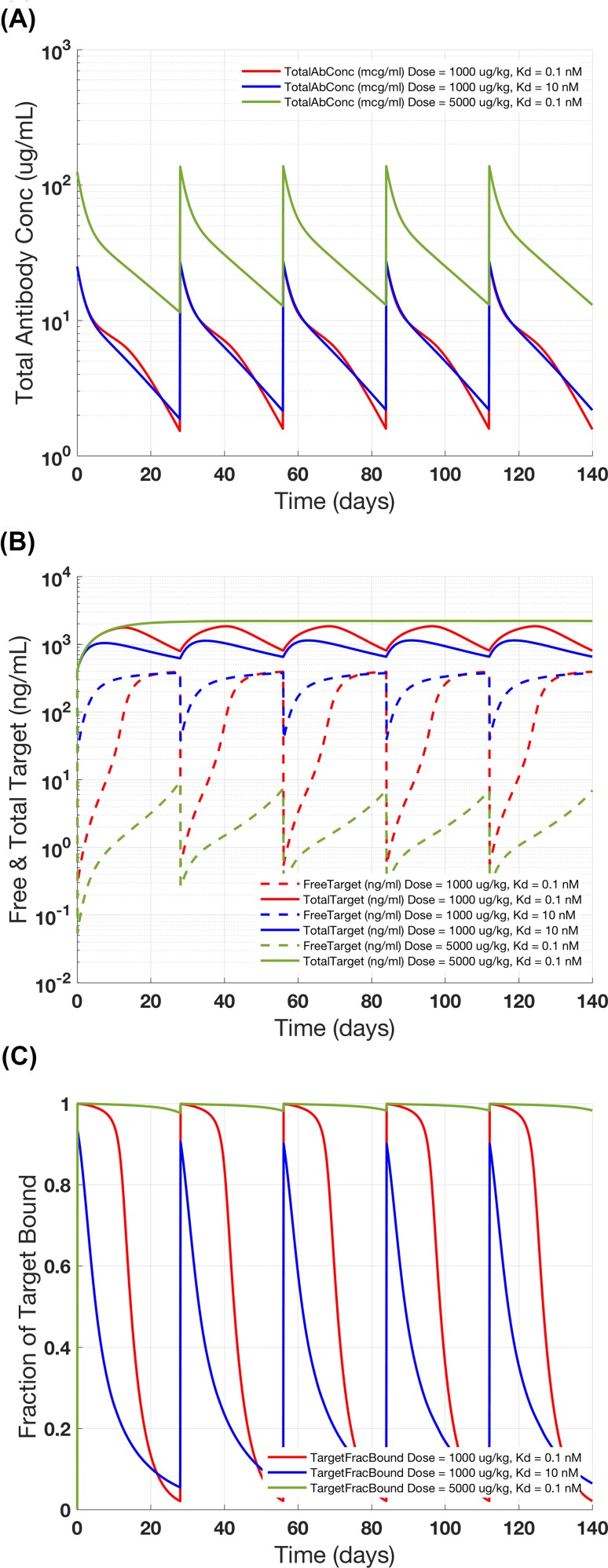
Case Study #2: target-mediated drug disposition model. The results show the impact of antibody affinity to target and dose level on **a** total antibody concentration, **b** free and total target concentration, and **c** fraction of target bound

The instructions in Supplementary Instruction S2 can be followed for working through this study (see Table S5 for initial conditions). The goal is to evaluate the impact of (1) antibody-target binding affinity and (2) dose level on target engagement. The settings for the simulations are described in Table 8. Figure 5a–c show the results for the TMDD model simulations. Comparing Simulations 2.1 and 2.2 (*K*_*D*_ = 0.1 and 10 nM), we see that the fraction of the target that is antibody-bound remains high in the simulation with the higher affinity and drops with reducing antibody concentration but it is low over the course of the simulated time course using the low affinity antibody (Fig. 5c ). The exposure of the antibody in both simulations is comparable (Fig. 5a), indicating that while the difference in affinity significantly influences target binding, it does not impact drug PK. The profiles for free and total (bound + free) target for the two simulations are shown in Fig. 5b. The effect of dose is seen where we compare the target engagement of Simulation 1 (1000 μg/kg dose Q4 W) with Simulation 2.3 (5000 μg/kg dose Q4 W). In this case, the model suggests that with 5 times higher dose and systemic drug exposure, the target remains > 98% bound over the entire simulation time-window.

**Table 8:** gPKPDSim settings used for Case Study #2

	Simulation 2.1	Simulation 2.2	Simulation 2.3
Dose level (μg/kg)	1000	1000	5000
Target initial value (nM)	10	10	10
*K*_*D*_ (nM)	0.1	10	0.1

The case study demonstrates how scientists can use gPKPDSim to answer some typical questions in drug development such as “knowing the target levels and turn over rate, at what dose level does the impact of TMDD on the PK profile become negligible?”, “Is the profile of total antibody different from the free antibody?”, etc. The end-user can also test “what if” scenarios for the particular antibody of interest, for example by selecting dose levels, frequency and changing parameter values using sliders. The interactive plots allow visualization of different outputs across multiple simulations with appropriate scales (log/linear) for the y-axis. The user can also save the figures to be used in presentations, documents and manuscripts. Further, the results are saved within the session file, which serves as a useful documentation for the future use.

### Case Study #3: physiologic indirect response models to link PK with PD biomarkers

Beyond PK and target engagement or inhibition, another important consideration in pharmaceutical sciences is the biological impact as measured by pharmacodynamics (PD) biomarkers. Basic PD models have been classified as direct or indirect models. This section illustrates the use of gPKPDSim with a two-compartment PK model integrated with either one of four basic physiologic indirect response models to characterize pharmacodynamics effects of a drug, whose mechanisms of action can be summarized as inhibition or stimulation of the synthesis or degradation of some endogenous target or mediator [6]. The selection of the desired indirect response model is provided as a variant selection in gPKPDSim. The model is described by the following set of differential equations:

19 $$\frac{{d A_{C} }}{dt} = k_{abs} \cdot f_{bio} \cdot A_{SC} - CL \cdot X_{C} - \frac{{V_{m} \cdot X_{C} }}{{K_{m} + X_{C} }} - CL_{d} \left( {X_{C} - X_{P} } \right),$$

20 $$\frac{{d A_{P} }}{dt} = CL_{d} \cdot \left( {X_{C} - X_{P} } \right),$$

21 $$\frac{{d A_{SC} }}{dt} = - k_{abs} \cdot A_{SC} ,$$

22 $$\frac{d AUC}{dt} = X_{C} ,$$

23 $$\frac{{d R_{1} }}{dt} = k_{in} \cdot \left( {1 - \frac{{X_{c} }}{{X_{c} + IC_{50} }}} \right) - k_{out} \cdot R_{1} ,$$

24 $$\frac{{d R_{2} }}{dt} = k_{in} - k_{out} \cdot R_{2} \cdot \left( {1 - \frac{{X_{c} }}{{X_{c} + IC_{50} }}} \right),$$

25 $$\frac{{d R_{3} }}{dt} = k_{in} \cdot \left( {1 + \frac{{E_{max} \cdot X_{c} }}{{X_{c} + EC_{50} }}} \right) - k_{out} \cdot R_{3} ,$$

26 $$\frac{{d R_{4} }}{dt} = k_{in} - k_{out} \cdot R_{4} \cdot \left( {1 + \frac{{E_{max} \cdot X_{c} }}{{X_{c} + EC_{50} }}} \right),$$

27 $$X_{C} = \frac{{A_{C} }}{{V_{1} }},$$

28 $$X_{P} = \frac{{A_{P} }}{{V_{2} }},$$

29 $$Response = \left\{ {\begin{array}{*{20}c} {R_{1} } & {for \, Inhibition \, on \, Synthesis} \\ {R_{2} } & {for \, Inhibition \, on \, Degradation} \\ {R_{3} } & {for \, Stimulation \, on \, Synthesis} \\ {R_{4} } & {for \, Inhibition \, on \, Degradation} \\ \end{array} } \right.$$

where Eqs. (19)–(22) describe the two-compartment PK model presented in Case Study #1 and Eqs. (23)–(26) describe the four basic physiologic indirect response models and the impact of drug concentration on synthesis (*k*_*in*_) or degradation (*k*_*out*_) of factors controlling the response variable (*R*_*x*_) via an inhibitory (*IC*_50_) or stimulatory (*EC*_50_ and *E*_*max*_) mechanism. Parameters *IC*_50_ and *EC*_50_ have the units of μg/mL, whereas *k*_*in*_, and *k*_*out*_ take the units that suit the problem of interest. Four variants are included in the session file, corresponding to the four basic models. Note that only one of these variants should be active at any given time (see Supplementary File casestudy3_IDR_TwoCompPK_equations.pdf for the units of parameters and species in the model).

**Fig. 6:**
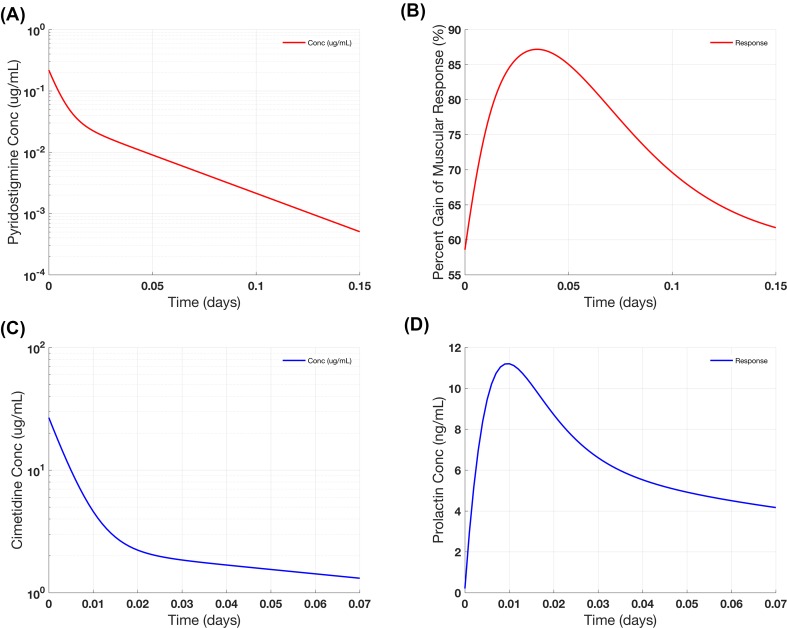
Case Study #3: physiologic indirect response model. Example 1 shows **a** the projected PK of pyridostigmine, and **b** patterns of percent gain of muscular response (using the inhibition on degradation model) after IV administration of 5 mg pyridostigmine. Example 2 shows **c** the projected PK of cimetidine, and **d** plasma concentration of prolactin (using the stimulation on synthesis model) after IV administration of 300 mg cimetidine

The first example (Simulation 3.1) in this section describes the effects of pyridostigmine on inhibition of cholinesterase, which leads to increased levels of acetylcholine in the neuromuscular junction and subsequently increases muscular activity, which is considered the PD effect in the treatment of myasthenia gravis [10, 11]. Here, we aim to replicate the PK profile and the muscle response (Fig. 6 from [6]) in a patient that received a 5 mg dose of intravenous pyridostigmine. The PK of pyridostigmine has a bi-exponential decay and can be described by a two-compartment model with parameter values listed in Table 9 (the calculations are done for a 70 kg patient). For the PD effect, we use the second indirect response model (*R*_2_) with the inhibition on degradation. Profiles generated in Fig. 6a and b characterize the PK profile of pyridostigmine and its PD effect and corroborate the results reported in [6].

**Table 9:** gPKPDSim settings used for Case Study #3

	Simulation 3.1:	Simulation 3.2:
	Cholinesterase inhibition	Prolactin secretion
IDR model	*R*_2_ (Inhibition-kout)	*R*_3_ (stimulation-kin)
Dose level	71.4 μg/kg	4286 μg/kg
*V*_1_	327.7 mL/kg	160.1 mL/kg
*V*_2_	438 mL/kg	827 mL/kg
*CL*	33,180 mL/day/kg	10,984 mL/day/kg
*CLd*	26,902 mL/day/kg	22,800 mL/day/kg
*K*_*in*_	2362 gain/day	21.6 ng/mL/day
*K*_*out*_	40.3 day^−1^	105.1 day^−1^
*IC*_50_	0.029 μg/mL	N/A
*EC*_50_	N/A	14.3 μg/mL
*E*_*max*_	N/A	212
Duration	0.15 day	0.07 day

The second example (Simulation 3.2) describes the impact of cimetidine on stimulating secretion of prolactin [12]. The plasma concentration of cimetidine after IV administration of 300 mg in patients can be characterized by a two-compartment model. For the PD effect, we use the third indirect response model (*R*_3_) with the stimulation on synthesis. Profiles generated in Fig. 6c and d describe cimetidine PK profile and its effect on prolactin concentration and agree with the results reported in Fig. 9 of [6]. The case study presented here showcases how scientists can reproduce and validate models from literature, use them to inform their project and help them design their studies. The instructions for this case study is provided Supplementary Instruction S3 (see Table S5 for initial conditions).

### Case Study #4: minimal physiologically based pharmacokinetic (PBPK) model

In this example, we use a minimal antibody PBPK model, which incorporates target mediated drug disposition by including target-binding in plasma and two groups of tissues, namely, leaky and tight tissues [7]. As in the original publication, the model assumes that only free antibody flows between compartments and the drug-receptor complex is immobile. The binding equations in the model were revised to fix a typographical error in the original paper [personal communication with the author]. Additionally, the model now enables representation of the target in all compartments, simultaneously. The differential equations for this model are:

30 $$\frac{{d A_{C, free} }}{dt} = L \cdot X_{L, free} - CL_{c} \cdot X_{C, free} - \left( {\left( {1 - \sigma_{TT} } \right) \cdot L_{TT} + \left( {1 - \sigma_{LT} } \right) \cdot L_{LT} } \right) \cdot X_{C, free} - \left( {k_{on} \cdot X_{{C, free\left( {nM} \right)}} \cdot T_{{C, free\left( {nM} \right)}} - k_{on} \cdot K_{D} \cdot C_{{C\left( {nM} \right)}} } \right) \cdot MW_{Ab} \cdot V_{c} ,$$

31 $$\frac{{d A_{TT, free} }}{dt} = \left( {1 - \sigma_{TT} } \right) \cdot L_{TT} \cdot X_{C, free} - \left( {1 - \sigma_{L} } \right) \cdot L_{TT} \cdot X_{TT, free} - \left( {k_{on} \cdot X_{{TT, free\left( {nM} \right)}} \cdot T_{{TT, free\left( {nM} \right)}} - k_{on} \cdot K_{D} \cdot C_{{TT\left( {nM} \right)}} } \right) \cdot MW_{Ab} \cdot V_{TT} ,$$

32 $$\frac{{d A_{LT, free} }}{dt} = \left( {1 - \sigma_{LT} } \right) \cdot L_{LT} \cdot X_{C, free} - \left( {1 - \sigma_{L} } \right) \cdot L_{LT} \cdot X_{LT, free} - \left( {k_{on} \cdot X_{{LT, free\left( {nM} \right)}} \cdot T_{{LT, free\left( {nM} \right)}} - k_{on} \cdot K_{D} \cdot C_{{LT\left( {nM} \right)}} } \right) \cdot MW_{Ab} \cdot V_{TT} ,$$

33 $$\frac{{d A_{L, free} }}{dt} = \left( {1 - \sigma_{L} } \right) \cdot L_{TT} \cdot X_{TT, free} + \left( {1 - \sigma_{L} } \right) \cdot L_{LT} \cdot X_{LT, free} - L \cdot X_{L, free} ,$$

34 $$\frac{{d T_{C, free(nM)} }}{dt} = k_{C, deg} \cdot T_{{C, init\left( {nM} \right)}} - k_{C, deg} \cdot T_{{C, free\left( {nM} \right)}} - \left( {k_{on} \cdot X_{{C, free\left( {nM} \right)}} \cdot T_{{C, free\left( {nM} \right)}} - k_{on} \cdot K_{D} \cdot C_{{C\left( {nM} \right)}} } \right),$$

35 $$\frac{{d T_{TT, free(nM)} }}{dt} = k_{TT, deg} \cdot T_{{TT, init\left( {nM} \right)}} - k_{TT, deg} \cdot T_{{TT, free\left( {nM} \right)}} - \left( {k_{on} \cdot X_{{TT, free\left( {nM} \right)}} \cdot T_{{TT, free\left( {nM} \right)}} - k_{on} \cdot K_{D} \cdot C_{{TT\left( {nM} \right)}} } \right),$$

36 $$\frac{{d T_{LT, free(nM)} }}{dt} = k_{LT, deg} \cdot T_{{LT, init\left( {nM} \right)}} - k_{LT, deg} \cdot T_{{LT, free\left( {nM} \right)}} - \left( {k_{on} \cdot X_{{LT, free\left( {nM} \right)}} \cdot T_{{LT, free\left( {nM} \right)}} - k_{on} \cdot K_{D} \cdot C_{{LT\left( {nM} \right)}} } \right),$$

37 $$\frac{{d C_{C(nM)} }}{dt} = \left( {k_{on} \cdot X_{{C, free\left( {nM} \right)}} \cdot T_{{C, free\left( {nM} \right)}} - k_{on} \cdot K_{D} \cdot C_{{C\left( {nM} \right)}} } \right) - k_{int} \cdot C_{{C\left( {nM} \right)}} ,$$

38 $$\frac{{d C_{TT(nM)} }}{dt} = \left( {k_{on} \cdot X_{{TT, free\left( {nM} \right)}} \cdot T_{{TT, free\left( {nM} \right)}} - k_{on} \cdot K_{D} \cdot C_{{TT\left( {nM} \right)}} } \right) - k_{int} \cdot C_{{TT\left( {nM} \right)}} ,$$

39 $$\frac{{d C_{LT(nM)} }}{dt} = \left( {k_{on} \cdot X_{{LT, free\left( {nM} \right)}} \cdot T_{{LT, free\left( {nM} \right)}} - k_{on} \cdot K_{D} \cdot C_{{LT\left( {nM} \right)}} } \right) - k_{int} \cdot C_{{LT\left( {nM} \right)}} ,$$

40 $$X_{C, free} = \frac{{A_{C, free} }}{{V_{c} }},$$

41 $$X_{C, free(nM)} = \frac{{X_{C, free} }}{{MW_{Ab} }},$$

42 $$X_{TT, free} = \frac{{A_{TT, free} }}{{V_{TT} }},$$

43 $$X_{TT, free(nM)} = \frac{{X_{TT, free} }}{{MW_{Ab} }},$$

44 $$X_{LT, free} = \frac{{A_{LT, free} }}{{V_{LT} }},$$

45 $$X_{LT, free(nM)} = \frac{{X_{LT, free} }}{{MW_{Ab} }},$$

46 $$X_{L, free} = \frac{{A_{L, free} }}{{V_{L} }}.$$

Similar to Case Study #2, *A*_*C, free*_, *A*_*TT, free*_, *A*_*LT, free*_, and *A*_*L, free*_ represent the amount of free drug in the central (plasma), tight tissues, leaky tissues and lymph. The concentration of free target and the drug:target complex in plasma is represented by *T*_*C, free(nM)*_ and *C*_*c(nM)*_. The concentration of free drug in plasma is denoted by *X*_*C, free*_. Similar nomenclature was used to define the concentration of drug, target and complex in leaky and tight tissues. Unless otherwise noted, the units for antibody amounts, and concentrations are μg, and μg/L, whereas the units for the concentration of target and complex are nM. The volume of plasma, tight and leaky tissues and lymph are represented by *V*_*C*_, *V*_*TT*_, *V*_*LT*_, and *V*_*L*_. Parameters *σ*_*TT*_ and *σ*_*LT*_ represent vascular reflection coefficients for tight and leaky tissues and *L*_*TT*_ and *L*_*LT*_ are lymph flows in these tissues. Parameters *L* and *σ*_*L*_ denote lymphatic flow and reflection coefficient. Parameters *k*_*on*_, *K*_*D*_, *MW*_*Ab*_, and *k*_*int*_ represent association rate constant, dissociation constant, molecular weight of drug, and internalization rate of the drug:target complex, whereas *k*_*x,deg*_, and *T*_*x,init(nM)*_ denote the degradation rate constant and initial amount of target in compartment *x* (see Supplementary File casestudy4_minPBPK_final.pdf for the units of parameters and species in the model).

**Fig. 7:**
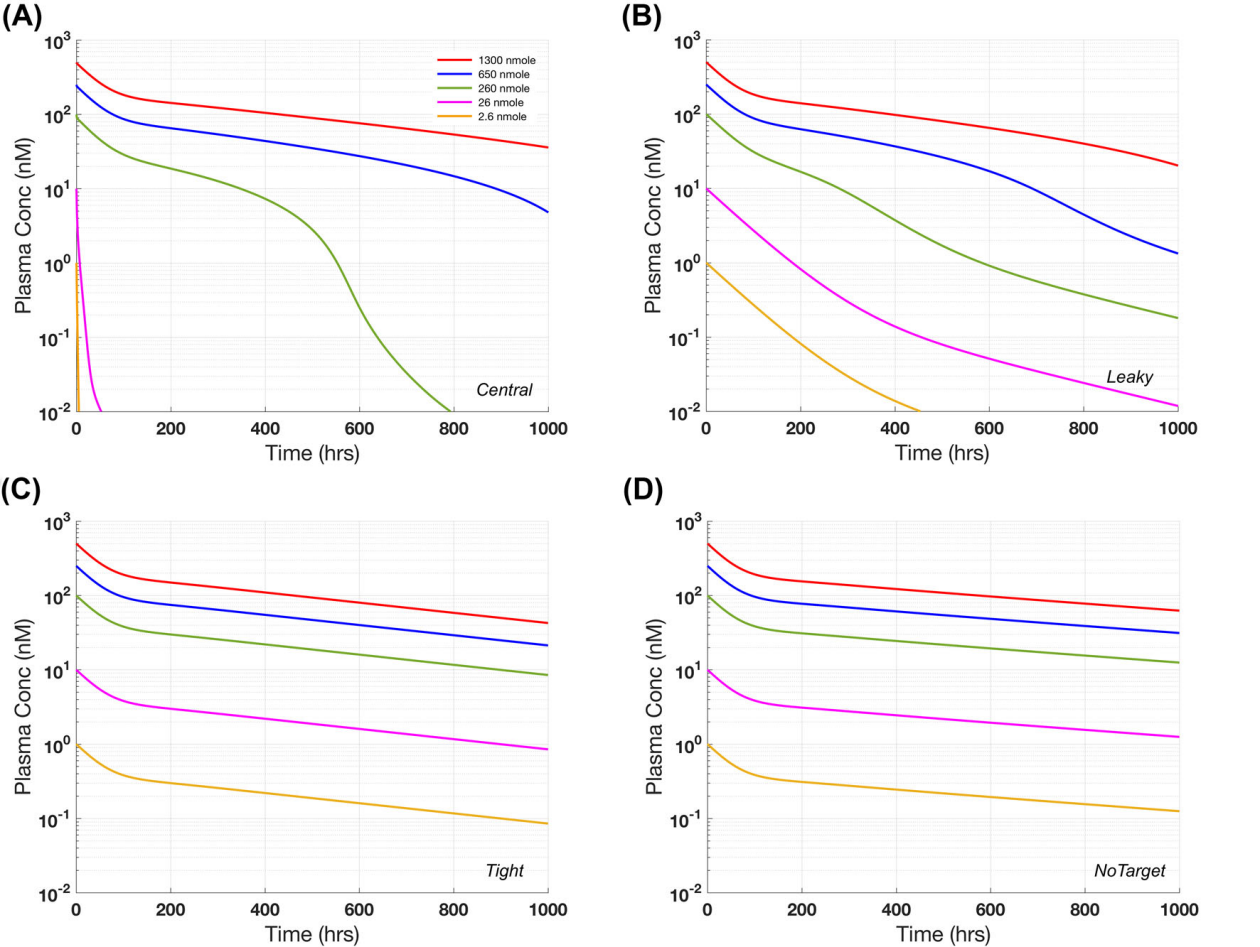
Case Study #4: minimal physiologically based pharmacokinetic (PBPK) model. Plasma concentration versus time profiles for increasing doses from 2.6 to 1300 nmole based on target-mediated drug disposition in **a** central compartment, **b** leaky tissues, **c** tight tissues, or **d** no target in any compartment

Here, we replicate the simulated plasma concentrations in the presence of 10 nM target in plasma, leaky or tight tissues (Fig. 2 from [7]) and compare the results with the case of no target. The parameter values are taken from Table 1 from [7], where *CLp* should have been listed as 10^−4^ L/h/kg. Figure 7a and b show nonlinear profiles when target is present in circulation or tight tissues where nonlinearity is more pronounced for the target in the central compartment. In contrast, in tight tissues, the PK profile appears to be dose proportional (Fig. 7c). When compared to the case with no target, we observed that overall clearance is slightly higher when target is present in tight tissues (Fig. 7d). Note the difference in PK profiles depicted in this paper and Fig. 2 of [7] (this is due to the typographical error in the original binding equations, corrected here). The instructions in Supplementary Box 4 can be followed for generating Fig. 7a–d (the instructions for this case study is provided Supplementary Instruction S4; see Table S5 for initial conditions). Once again, the case study presented here showcases how researchers can use gPKPDSim to corroborate the results from published papers, compare the results with other scenarios that may not have been included in the original publications and use those to informal internal decisions.

## Discussion

With the increasing application of PKPD modeling in pharmaceutical research and development, one key challenge is to *efficiently* deploy models built by expert modelers and enable scientists and collaborators with limited M&S experience to *correctly* use these models to answer research questions. To do so, we have developed gPKPDSim, a multi-purpose MATLAB^®^ application with a user-friendly interface for deploying SimBiology^®^ models and allowing scientists to perform various PKPD analyses and model-based tasks, regardless of their modeling expertise. The application has a single screen interface to perform common PKPD tasks and includes additional capabilities that may not currently exist in SimBiology^®^, but “hides” the MATLAB^®^ code and involved features of SimBiology^®^ from end-users. And therefore it removes a significant bottleneck of software training for most end-users. Furthermore, modeling experts need not perform all routine analyses, but can serve instead as advisors and reviewers on these tasks, increasing their bandwidth for more complex modeling and analysis efforts.

In this work, we have used MATLAB^®^ and SimBiology^®^ as the engine behind gPKPDSim because they provide a powerful and flexible platform to build, simulate, and analyze mechanistic PKPD and systems biology models. Note that several programming and modeling platforms are available to the M&S community and similar applications can be developed in some of these platforms as well. Note that gPKPDSim provides PKPD M&S capabilities to users in an organization with access to SimBiology^®^/MATLAB^®^ at no additional cost; however, each organization has to make an informed investment based on their needs in software tools such as SimBiology^®^/MATLAB^®^ and others that have costs associated with them.

Along with the gPKPDSim application, we have provided a library of PKPD models including (1) a two-compartment pharmacokinetics model with specific and non-specific clearance, (2) a target-mediated drug disposition (TMDD) model, (3) a physiologic indirect response model and (4) a minimal physiologically based pharmacokinetic (PBPK) model with the drug target represented in the central compartment, leaky and tight tissues. These models have a wide range of applications in drug development such as characterization of PKPD relationships, molecule selection, human PK projection, and preclinical and clinical dose and schedule determination, especially for antibody therapeutics, and the application can be used with additional models (e.g., small molecule PK). The case studies presented in this paper demonstrate how scientists familiar with the concepts of PKPD modeling can efficiently use gPKPDSim to analyze PKPD data and address their drug research and development questions. Furthermore, the application can be used for exploratory research with PKPD models for hypothesis testing and evaluation of what-if scenarios. It also enables easy sharing and communication of the results.

The application is open-source; thus the M&S community can extend software as they see fit for their respective use. Additionally, gPKPDSim works with any SimBiology^®^-based model, providing flexibility to researchers developing their own models. gPKPDSim is also equipped with the NCA functionality, which can handle datasets that have IV and/or extravascular dosing regimens in single-dose or multi-dose scenarios with sparse and serial sampling schemes. This is an extra benefit over WinNonlin^®^, in which these different scenarios must be provided in multiple datasets to calculate the NCA results. The population simulation functionality in gPKPDSim is intended for evaluation of parameter variability on PKPD predictions primarily in the preclinical and translational settings; this functionality should not be used for applications that require a formal population PK model.

At Genentech, gPKPDSim was initially developed for and is now regularly used by PKPD scientists working on preclinical/translational PKPD of biologics and large molecules. We have found that by empowering pharmaceutical scientists and drug development team members to conduct straightforward model-based tasks, gPKPDSim encourages broader use of PKPD models by collaborators across multiple functions to support drug development, optimize study design, reduce animal usage and help with decision-makings.

## Electronic supplementary material

Below is the link to the electronic supplementary material.

Electronic supplementary material 1 (DOCX 717 kb)

Electronic supplementary material 2 (ZIP 7898 kb)
