# Supplementary material for: gPKPDSim: a SimBiology®-based GUI application for PKPD modeling in drug development
Source: J Pharmacokinet Pharmacodyn. 2018 Jan 4;45(2):259–75. doi: 10.1007/s10928-017-9562-9 (PMC5845055; doi:10.1007/s10928-017-9562-9)
Supplement: Supplementary file 1 — Electronic supplementary material 1 (DOCX 717 kb) [file 10928_2017_9562_MOESM1_ESM.docx]

TITLE

gPKPDSim: A SimBiology^®^-based GUI application for PKPD modeling in drug development

AUTHORS

Iraj Hosseini^1,*^, Anita Gajjala^2^, Daniela Bumbaca Yadav^1^, Siddharth Sukumaran^1^, Saroja Ramanujan^1^, Ricardo Paxson^3^, and Kapil Gadkar^1^

^1^ Genentech Inc., South San Francisco, CA

^2^ MathWorks Inc., Consulting Services, Natick, MA

^3^ MathWorks Inc., Natick, MA

^*^ Corresponding author (email: hosseini.iraj@gene.com)

**SUPPLEMENTARY MATERIAL**

SUPPLEMENTARY FIGURES

**Fig. S1. SimBiology Variant Example.** The top table shows the parameter values stored in Var1 and Var2. In the bottom table, column 2 shows the default parameter values in the mode, whereas columns 3 and 4 show how parameter values change when each variant is applied. Columns 5 and 6 show how parameter values change when variants are applied in different orders.


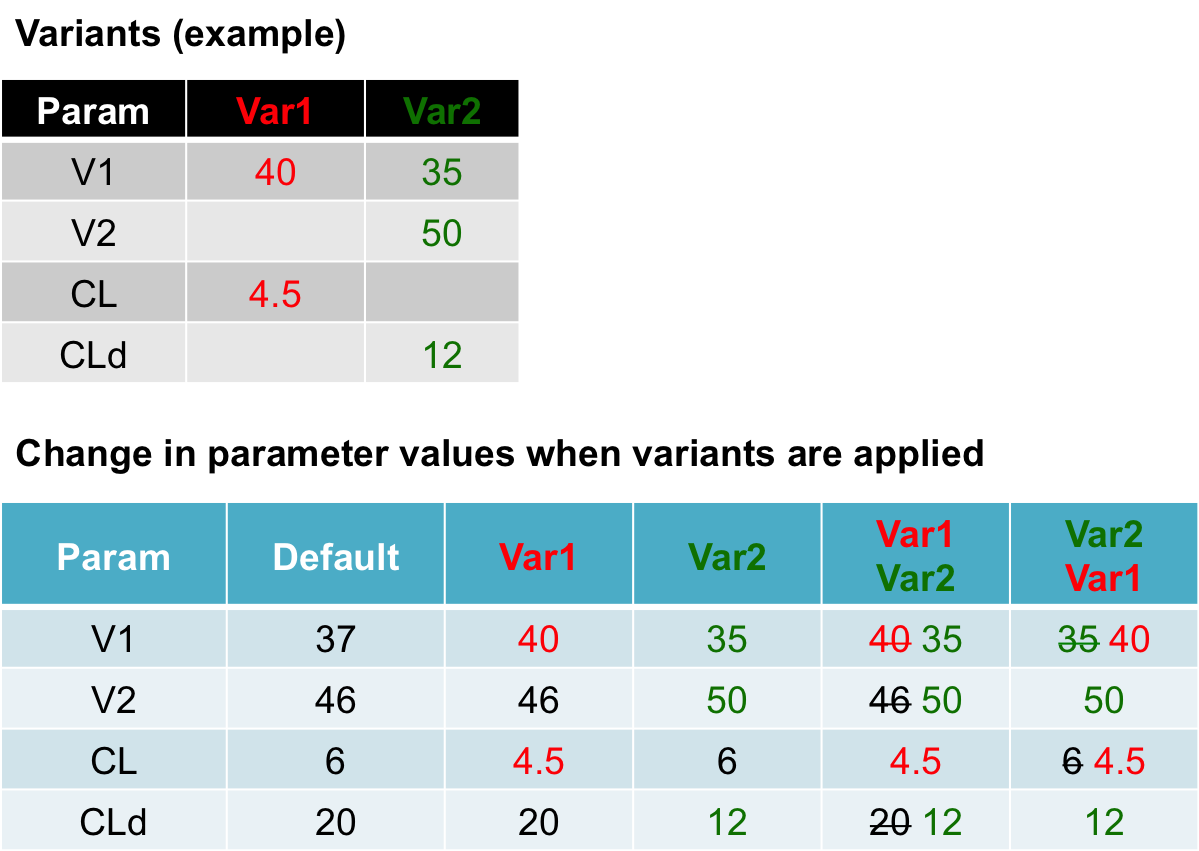


**Fig S2. gPKPDSim: Import Dataset.** This view enables the user to import a dataset for the purpose of visualization, data fitting, and/or NCA. The imported dataset must be in a specific format defined in Supplementary Method S2. See Methods for detailed description of each field.


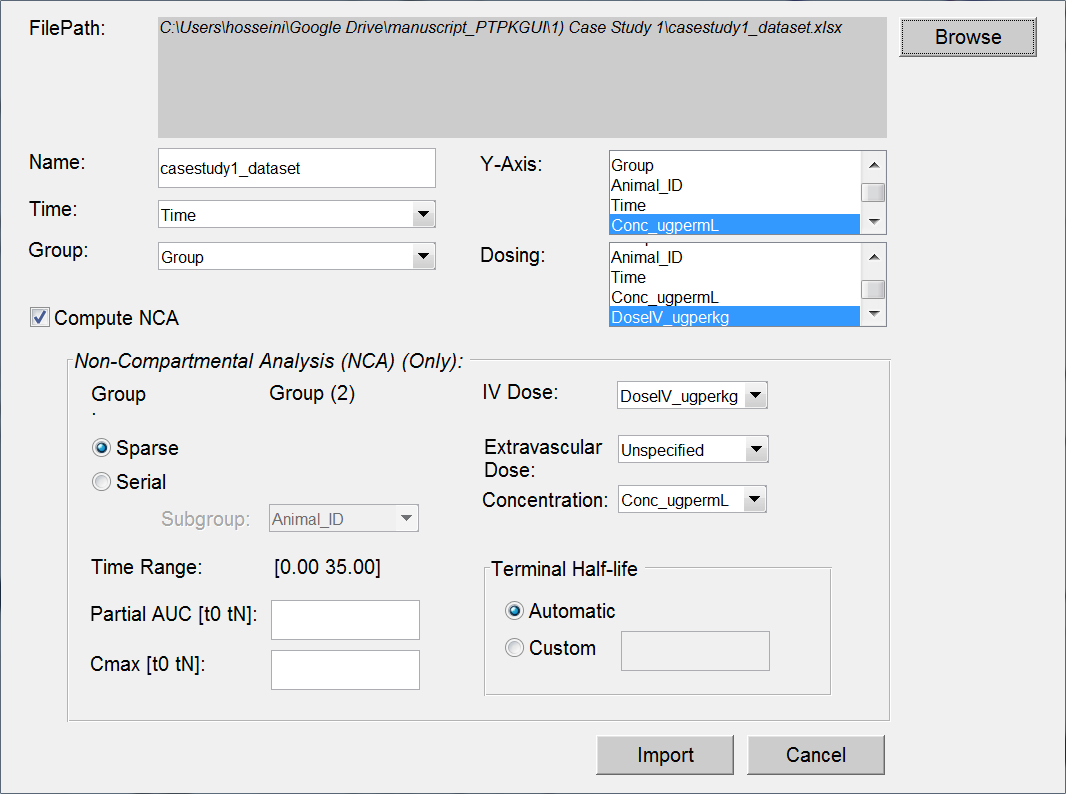


**Fig S3. gPKPDSim: Data Fitting Functionality View.** This view has multiple sections: 1) general settings (top left); 2) functionality-specific settings including dose mapping, species mapping, pooled fitting, error model, and selection of parameters to be estimated (bottom left); 3) plots (top middle). See Supplementary Methods for detailed description of each section.


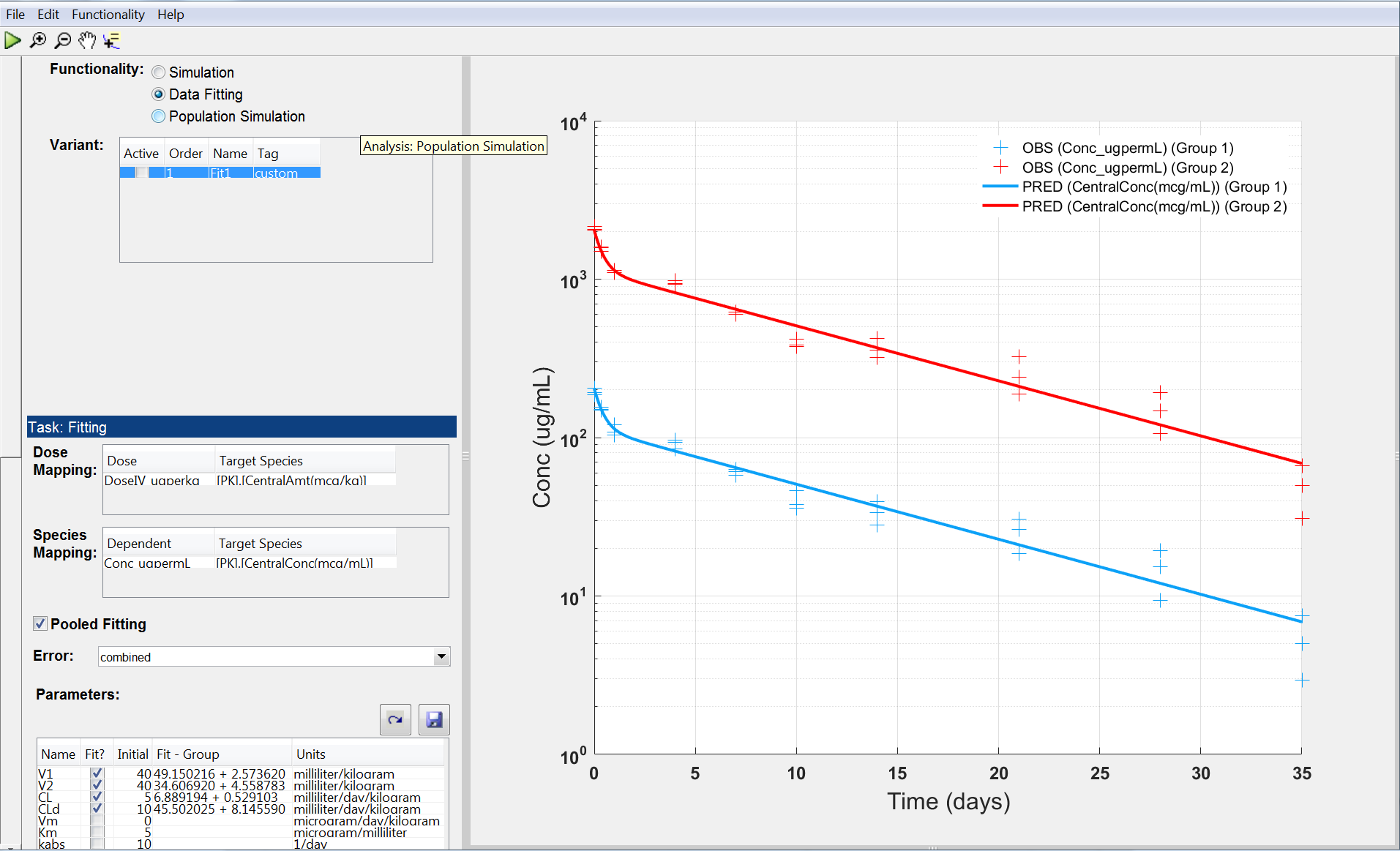


**Fig S4. gPKPDSim: Simulation Population Functionality View.** This view has multiple sections similar to the simulation functionality view. The only difference is that in the functionality-specific settings, in addition to simulation time and parameter values, the user can specify the number of simulations and the percent CV variability for each parameter (bottom left).


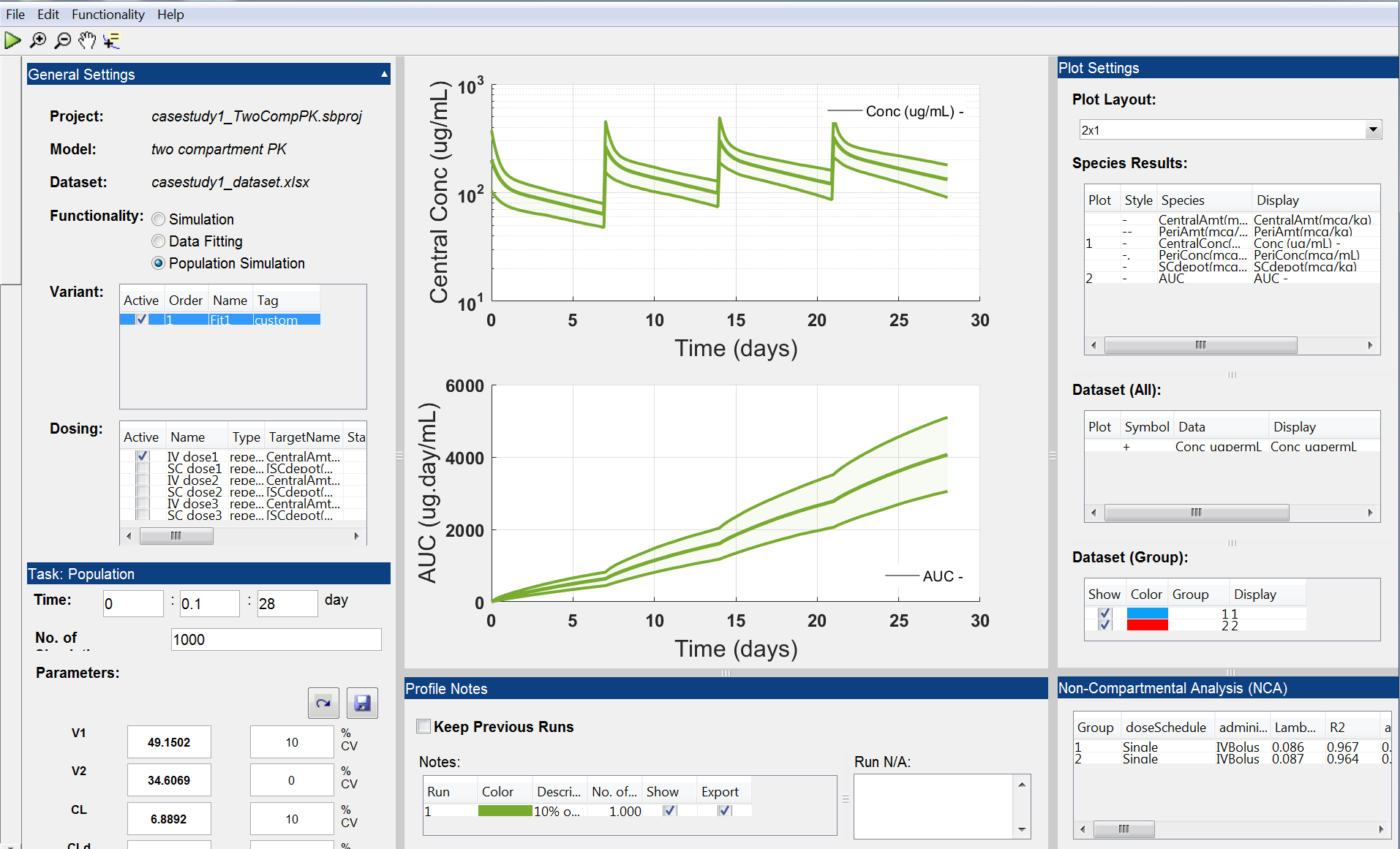


SUPPLEMENTARY TABLES

**Table S1.** A sample dataset, for which the dosing information is defined at a group level.

| **Include** | **Group** | **Subject** | **Time** | **Conc_ugmL** | **DoseIV** | **DoseEV** |
| --- | --- | --- | --- | --- | --- | --- |
|  | 1 |  | 0 |  | 10000 |  |
|  | 1 | 1.1 | 0.01047 | 5 |  |  |
|  | 1 | 1.2 | 0.01047 | 7 |  |  |
|  | 1 | 1.3 | 0.01047 | 3 |  |  |
|  | 1 | 1.1 | 0.3333333 | 80 |  |  |
|  | 1 | 1.2 | 0.3333333 | 85 |  |  |
|  | 1 | 1.3 | 0.3333333 | 77 |  |  |
|  | 1 | 1.1 | 1 | 190 |  |  |
|  | 1 | 1.2 | 1 | 195 |  |  |
|  | 1 | 1.3 | 1 | 187 |  |  |
|  | 2 |  | 0 |  |  | 10000 |
|  | 2 | 2.1 | 0.01047 | 90 |  |  |
|  | 2 | 2.2 | 0.01047 | 95 |  |  |
|  | 2 | 2.3 | 0.01047 | 87 |  |  |
|  | 2 | 2.1 | 0.3333333 | 60 |  |  |
|  | 2 | 2.2 | 0.3333333 | 65 |  |  |
|  | 2 | 2.3 | 0.3333333 | 57 |  |  |
|  | 2 | 2.1 | 1 | 40 |  |  |
|  | 2 | 2.2 | 1 | 45 |  |  |
|  | 2 | 2.3 | 1 | 37 |  |  |

**Table S2.** A sample dataset, for which the dosing information is defined at a subject (subgroup) level.

| **Include** | **Group** | **Subject** | **Time** | **Conc_ugmL** | **DoseIV** | **DoseEV** |
| --- | --- | --- | --- | --- | --- | --- |
|  | 1 | 1.1 | 0 |  | 10000 |  |
|  | 1 | 1.2 | 0 |  | 10000 |  |
|  | 1 | 1.3 | 0 |  | 10000 |  |
|  | 1 | 1.1 | 0.01047 | 5 |  |  |
|  | 1 | 1.2 | 0.01047 | 7 |  |  |
|  | 1 | 1.3 | 0.01047 | 3 |  |  |
|  | 1 | 1.1 | 0.3333333 | 80 |  |  |
|  | 1 | 1.2 | 0.3333333 | 85 |  |  |
|  | 1 | 1.3 | 0.3333333 | 77 |  |  |
|  | 1 | 1.1 | 1 | 190 |  |  |
|  | 1 | 1.2 | 1 | 195 |  |  |
|  | 1 | 1.3 | 1 | 187 |  |  |
|  | 2 | 2.1 | 0 |  |  | 10000 |
|  | 2 | 2.2 | 0 |  |  | 10000 |
|  | 2 | 2.3 | 0 |  |  | 10000 |
|  | 2 | 2.1 | 0.01047 | 90 |  |  |
|  | 2 | 2.2 | 0.01047 | 95 |  |  |
|  | 2 | 2.3 | 0.01047 | 87 |  |  |
|  | 2 | 2.1 | 0.3333333 | 60 |  |  |
|  | 2 | 2.2 | 0.3333333 | 65 |  |  |
|  | 2 | 2.3 | 0.3333333 | 57 |  |  |
|  | 2 | 2.1 | 1 | 40 |  |  |
|  | 2 | 2.2 | 1 | 45 |  |  |
|  | 2 | 2.3 | 1 | 37 |  |  |

**Table S3.** NCA Results for the serial sampling scheme from WinNonlin^®^ (two groups × three animals = six sets of NCA parameters)

|  | **WinNonlin^®^** | | | | | |
| --- | --- | --- | --- | --- | --- | --- |
| *Group* | 1 | 1 | 1 | 2 | 2 | 2 |
| *Dose Schedule* | Single | Single | Single | Single | Single | Single |
| *Administration Route* | IV Bolus | IV Bolus | IV Bolus | IV Bolus | IV Bolus | IV Bolus |
| *Animal_ID* | A | B | C | D | E | F |
| *Lambda_Z (1/day)* | 0.085 | 0.076 | 0.102 | 0.087 | 0.077 | 0.101 |
| *R2* | 0.956 | 0.963 | 0.980 | 0.964 | 0.945 | 0.979 |
| *adjusted_R2* | 0.951 | 0.957 | 0.977 | 0.959 | 0.937 | 0.976 |
| *Num_points* | 9 | 9 | 9 | 9 | 9 | 9 |
| *AUC_0_last (day.ug/mL)* | 1384.6 | 1565.7 | 1217.9 | 14012.4 | 15591.9 | 13023.23 |
| *Tlast (day)* | 35 | 35 | 35 | 35 | 35 | 35 |
| *C_max (ug/mL)* | 191.3 | 205.7 | 186.7 | 2051.2 | 2145.9 | 2042.8 |
| *C_max_Dose (kg.ug/mL/ug)* | 19.1 | 20.6 | 18.7 | 20.5 | 21.5 | 20.4 |
| *T_max (day)* | 0.01047 | 0.01047 | 0.01047 | 0.01047 | 0.01047 | 0.01047 |
| *MRT (day)* | 11.6 | 12.8 | 9.7 | 11.3 | 12.7 | 9.8 |
| *T_half (day)* | 8.2 | 9.1 | 6.8 | 8.0 | 9.0 | 6.9 |
| *AUC_infinity (day.ug/mL)* | 1443.7 | 1663.5 | 1246.9 | 14585.4 | 16448.2 | 13330.7 |
| *AUC_infinity_dose (day.kg.ug/mL/ug)* | 144.4 | 166.3 | 124.7 | 145.9 | 164.5 | 133.3 |
| *AUC_extrap_percent (%)* | 4.1 | 5.9 | 2.3 | 3.9 | 5.2 | 2.3 |
| *CL (mL/day/kg)* | 6.9 | 6.0 | 8.0 | 6.9 | 6.1 | 7.5 |
| *DM (ug/kg)* | 10000 | 10000 | 10000 | 100000 | 100000 | 100000 |
| *V_z (mL/kg)* | 81.9 | 78.7 | 79.0 | 78.9 | 78.7 | 74.5 |
| *AUMC_0_last (day.day.ug/mL)* | 13984.1 | 16580.5 | 10791.4 | 137861.0 | 167597.1 | 116225.4 |
| *AUMC (day.day.ug/mL)* | 16750.1 | 21282.4 | 12093.8 | 164507.2 | 208641.3 | 130042.0 |
| *AUMC_extrap_percent (%)* | 16.5 | 22.1 | 10.8 | 16.2 | 19.7 | 10.6 |
| *V_ss (mL/kg)* | 80.4 | 76.9 | 77.9 | 77.3 | 77.1 | 73.2 |
| *C_0 (ug/mL)* | 192.8 | 207.6 | 188.1 | 2068.8 | 2166.5 | 2063.2 |

**Table S4.** List of datasets used to compare NCA results from gPKPDSim and WinNonlin^®^

| **Dataset No.** | **Administration Route** | **Dose Schedule** | **No. of Groups** | **No. of Subjects / Group** | **Type of Sampling** | **Dose Levels (mg/kg)** | **Duration (days)** |
| --- | --- | --- | --- | --- | --- | --- | --- |
| 1 | IV | Single | 6 | 4 | Serial | 10 | 35 |
| 2 | SC | Single | 6 | 4 | Serial | 10 | 35 |
| 3 | IV | Single | 6 | 9 | Sparse | 10 | 28 |
| 4 | SC | Single | 6 | 9 | Sparse | 10 | 28 |
| 5 | IV | Single | 6 | 3 | Serial | 10 | 28 |
| 6 | SC | Single | 6 | 3 | Serial | 10 | 28 |
| 7 | IV | Single | 4 | 18 | Sparse | 40 | 42 |
| 8 | IV | Single | 3 | 5 | Serial | 5 | 20 |
| 9 | IV | Single | 4 | 6 | Sparse | 0.1, 10, and 50 | 14 |
| 10 | IV | Multi | 1 | 5 | Sparse | 30 | 54 |
| 11 | IV | Single & Multi | 4 | 3 | Sparse | 10 and 100 | 35 |
| 12 | SC | Single & Multi | 4 | 3 | Sparse | 5 and 50 | 35 |
| 13 | IV & SC | Single & Multi | 4 | 3 | Serial | 1, 15, and 150 | 35 |

**Table S5.** Initial conditions for Case Studies #1, #2, #3, and #4

| **Case Study #1** | | | **Case Study #3** | | |
| --- | --- | --- | --- | --- | --- |
| **Parameter** | **Value** | **Units** | **Parameter** | **Value** | **Units** |
| *V1* | 40 | mL/kg | *V1* | 40 | mL/kg |
| *V2* | 40 | mL/kg | *V2* | 40 | mL/kg |
| *CL* | 5 | mL/day/kg | *CL* | 5 | mL/day/kg |
| *CLd* | 10 | mL/day/kg | *CLd* | 10 | mL/day/kg |
| *Vm* | 0 | μg/day/kg | *Vm* | 0 | μg/day/kg |
| *Km* | 5 | μg/mL | *Km* | 5 | μg/mL |
| *kabs* | 10 | 1/day | *kabs* | 10 | 1/day |
| *fbio* | 0.7 | fraction | *fbio* | 0.7 | fraction |
| **Case Study #2** | | | *kin* | 0.1 |  |
| **Parameter** | **Value** | **Units** | *kout* | 0.1 |  |
| *V1* | 40 | mL/kg | *IC50* | 0.1 | μg/mL |
| *V2* | 40 | mL/kg | *EC50* | 0.1 | μg/mL |
| *CL* | 5 | mL/day/kg | *Emax* | 0.1 |  |
| *CLd* | 10 | mL/day/kg | **Case Study #4** | | |
| *kabs* | 10 | 1/day | **Parameter** | **Value** | **Units** |
| *fbio* | 0.7 | fraction | *Vplasma* | 2.6 | L |
| *kon* | 400 | 1/nM/day | *Vleaky* | 4.368 | L |
| *KD* | 0.1 | nM | *Vtight* | 8.112 | L |
| *MWtarget* | 40 | μg/nM | *Vlymph* | 5.2 | L |
| *MWab* | 150 | μg/nM | *CLp* | 0.07 | L/hr |
| *target_init* | 0 | nM | *sig_leaky* | 0.687 |  |
| *target_thalf* | 1 | day | *sig_tight* | 0.945 |  |
| *complCLfactor* | 1 | fold | *sig_lymph* | 0.2 |  |
|  |  |  | *CentralTarget0* | 0 | nM |
|  |  |  | *LeakyTarget0* | 0 | nM |
|  |  |  | *TightTarget0* | 0 | nM |
|  |  |  | *kdeg_central* | 0.01 | 1/hour |
|  |  |  | *kdeg_leaky* | 0.01 | 1/hour |
|  |  |  | *kdeg_tight* | 0.01 | 1/hour |
|  |  |  | *kon* | 0.1 | 1/nM/hour |
|  |  |  | *KD* | 0.01 | nM |
|  |  |  | *MWab* | 150 | μg/nM |
|  |  |  | *L_leaky* | 0.081 | L/hr |
|  |  |  | *L_tight* | 0.04 | L/hr |
|  |  |  | *L* | 0.121 | L/hr |
|  |  |  | *kint* | 0.03 | 1/hr |

SUPPLEMENTARY METHODS

**Method S1: How To Create A Session File**

In this section, we provide the MATLAB^®^ code to generate a session file. The assumption is that, the modeler has built and quality-checked a SimBiology^®^ model and aims to create a session file for it. To do so, the modeler needs to know the SimBiology^®^ project filename and SimBiology^®^ model’s name and decide which species, parameters, doses, and variants to be included. This code is available as “configuration.m” in the supplementary material.

% Configuration.m: code to generate a session file

clear;clc

% Create Analysis object

Analysis = PKPD.Analysis;

% Set sbproj file path

PathName = fileparts(which(mfilename));

ProjectPath = fullfile(PathName,'models','SimBiolModel.sbproj');

ModelName = 'Model1';

% Import SimBiology model

importModel(Analysis,ProjectPath,ModelName);

% Set the default functionality view when the session file is launched

Analysis.Task = 'Simulation';

%% Set debug flag; if true, parameter values are shown in MATLAB command window

Analysis.FlagDebug = true;

%% Set simulation Start time, time step, and stop time

Analysis.StartTime = 0;

Analysis.TimeStep = 1;

Analysis.StopTime = 28;

%% Set species to shown in gPKPDSim

Analysis.SelectedSpecies = Analysis.Species(1:end);

species_list = {'A', 'B', 'C', 'D'};

Analysis.SelectedSpecies = Analysis.Species(1);

for i = 1 : length(species_list)

Analysis.SelectedSpecies(i) = sbioselect(Analysis.Species, 'Name', species_list(i))

end

%% Set parameters to shown in gPKPDSim; for each parameter, the modeler should determine the default value, LB, UB, and Scale.

Analysis.SelectedParams = [...

PKPD.Parameter('Name','V1' ,'Value',40, 'Min',10,'Max',200,'Scale','log','FlagFit',false),...

PKPD.Parameter('Name','V2' ,'Value',40, 'Min',10,'Max',200,'Scale','log','FlagFit',false),...

PKPD.Parameter('Name','CL' ,'Value',5, 'Min',1, 'Max',30,'Scale','log','FlagFit',false),...

PKPD.Parameter('Name','CLd' ,'Value',10, 'Min',1, 'Max',100,'Scale','log','FlagFit',false)];

%% Set repeat doses to be shown in gPKPDSim

Match = strcmpi(get(Analysis.Doses,'Type'),'repeatdose');

Analysis.SelectedDoses = Analysis.Doses(Match);

%% Set variants to be shown in gPKPDSim; this could be empty if no variant is selected

Analysis.SelectedVariantNames = {'Variant1', 'Variant2'};

Analysis.SelectedVariantsOrder = 1:numel(Analysis.SelectedVariantNames);

%% Set fit function; if global optimization toolbox is installed, those methods can be used. UseFitBounds turns on/off the LB & UB columns in the parameter table in Data Fitting

Analysis.FitFunctionName = 'nlinfit';

Analysis.UseFitBounds = false;

%% Set the number of simulations for a population simulation

Analysis.NumPopulationRuns = 1000;

%% Set colormap1 for the profile notes

Analysis.ColorMap1 = [...

1 0 0

0 0 1

0 1 0

0.9686 0.0471 0.9529

0.9686 0.6314 0.0471

0.8 0.8 0.8

0 0 0

0.7529 0.9686 0.0471

];

%% Set colormap2 for groups in imported data

Analysis.ColorMap2 = [...

0.0471 0.6314 0.9686

1 0 0

0 1 0

];

%% Set line style for column headers in imported data

Analysis.LineStyleMap = {...

'-',...

'--',...

':',...

'-.',...

};

%% Save - must be saved with 'Analysis' name

[StatusOk,Message] = validate(Analysis);

if StatusOk

save('SimBiolModel_Session','Analysis')

else

error(Message)

end

**Method S2: Format of Import-Ready Datasets**

A gPKPDSim-compatible dataset has the following columns:

1. *Include:* The rows, for which this column is blank will be used for NCA, data fitting or visualization. If the value is “c” or “C”, that row will be ignored, when the data is used for analysis.
2. *Group:* Contains the Group information, which must have unique values. The end-user can define “Group” to suit the application of interest, e.g. a “Group” can be a dose arm/cohort of a study or can be each individual subject in a study. The choice of “Group” determines the type of NCA and data fitting that can be performed on the data (In the example below, the dataset has two groups).
3. *Subject:* Contains the subject information, e.g. the individual subjects within a Group, which must have unique values. This column is optional and can be used in Serial NCA or if the end-user aims to perform data fitting for each individual subject, separately (In the example below, each group has three subjects).
4. *Time:* Contains the time information. The units must be compatible with the units of time in the SimBiology^®^ model included in the session file.
5. *Concentration:* Contains the PK measurement at different time points. If PK is not measured for a specific time point for a group/subject, that row must be blank. The units must be compatible with the units of species corresponding to the drug concentration in the SimBiology^®^ model included in the session file (In Case Study #1, concentration has units of ug/mL).
6. *DoseIV:* Contains the IV dosing information. This column has a value at any given time point, the group or subjects within a group were dosed; otherwise, it is blank. If the dosing information is defined at a group level, only one dose value should be defined at any time a dose was given (Table S1). If the dosing information is defined at a subject level, one dose value should be defined per subject at any time a dose was given (Table S2). Additionally, this column can include both single-dose and multi-dose regimens, i.e. each group can have a different dosing regimen. Note that the concentration measurements are usually pre- or post-dose and there is no measurement at the time of dosing, which suggests that the value of the concentration column should be blank. The units must be compatible with the units of species corresponding to the drug amount in the SimBiology^®^ model included in the session file (In Case Study #1, dose has units of ug/kg).
7. *DoseEV:* It is similar to DoseIV except that it contains the extravascular dosing information. Note that a dataset can have columns corresponding IV dosing, extravascular dosing or both.
8. *Other measurements:* The dataset can have other columns corresponding to other measurements in the study.

**Method S3: gPKPDSim Menu Settings, Functionalities and Features**

***Menu Settings:***

*File > Open Session:* Loads a session file with a .mat extension.

*File > Open Recent Session:* Keeps a list of recently opened session files for easier access.

*File > Import Dataset:* Enables the user to import a dataset for the purpose of visualization, data fitting, and/or NCA. The imported dataset must be in a specific format defined in Supplementary Method S2. The import dialog has the following features (Fig. S2):

- *FilePath:* Opens a window to browse to an Excel file containing the dataset.
- *Name:* Describes the name of the dataset.
- *Time:* Maps the column header in the dataset that contains the time information.
- *Group:* Maps the column header in the dataset that contains the Group information. The user can define “Group” to suit their specific application, e.g. a “Group” can be a dose arm/cohort of a study or can be each individual subject in a study. The choice of “Group” determines the type of NCA and data fitting that can be performed on the data (see Case Study #1 for further information).
- *Y-axis:* Enables the user to select multiple column headers from the dataset to be used for the purpose of data fitting and/or visualization.
- *Dosing:* Enables the user to select multiple column headers from the dataset that contain the dosing information.
- *Compute NCA:* Enables the user to decide whether (non-compartmental analysis) NCA [8] should be computed on a dataset. Note that NCA is typically performed on PK datasets and not all applications require it. If checked, the following settings become available:
  - *Group:* Informs the user on how many unique groups exist in the “Group” column header.
  - *Sparse:* Suggests a PK sampling scheme in which not all the subjects were sampled at all the time points, i.e. a partial PK profile per subject. For this selection, the dose amount in the dataset is interpreted as the dose given to all the subjects in that group, and the NCA method uses the average of measurements at any given time point to calculate PK parameters.
  - *Serial:* Suggests a PK sampling scheme in which all the subjects were sampled at all the time points, i.e. a full PK profile per subject. For this selection, the user has the option to further map the column header in the dataset that contains the Subgroup information. If Subgroup is unspecified, the dose amount in the dataset is interpreted as the dose given to all the subjects in that group, and the NCA method calculates PK parameters for each subject. If Subgroup (i.e. subject) is specified, the dose amount in the dataset is interpreted as the dose given to the subject, and the NCA method calculates PK parameters for each subject. Further information on NCA functionality in SimBiology^®^/MATLAB^®^ is available at: https://www.mathworks.com/help/simbio/ug/non-compartmental-analysis.html.
  - *IV Dose and Extravascular Dose:* Maps the column headers in the dataset that contains the IV and extravascular dosing information. If that information doesn’t exist, the user should select unspecified. Note that a dataset can have either or both columns. Additionally, the column corresponding to IV dose (or extravascular dose) can include both single-dose and multi-dose regimens.
  - *Concentration:* Maps the column header in the dataset that contains the concentration data. The NCA is performed on only one column header in the dataset.
  - *Time Range:* Informs the user on the range of time points in the dataset.
  - *Partial AUC [t_0_ t_N_]:* Allows the user to provide an array of time ranges to estimate partial AUC values (for example, if the user aimed to calculate partial AUCs between 0-21 days and 7-14 days, the input would be [0 21; 7 14])
  - *C_max_ [t_0_ t_N_]:* Allows the user to provide multiple time ranges to calculate C_max_ values (the time format is the same as that of partial AUCs)
  - *Terminal Half-life (Automatic/Custom):* The “Automatic” option means that the NCA method determines the time points used in calculating the half-life, whereas the “Custom” option means that the user provides a time range [t_0_ t_N_], which determines the time points to be included in the estimation of terminal half-life.

*File > Save Session:* Saves the current session file.

*File > Save Session As:* Saves the current session file as a new file.

*File > Export:* Saves the output of different functionalities to Excel/pdf files. These include saving “Selected Simulation Results to Excel”, “Fitting Results To Excel”, “Fitting Summary to PDF”, “Selected Population Results to Excel”, and “Non-Compartment Analysis to Excel”. In Case Study #1, we have instructions to save the outputs of gPKPDSim functionalities.

*Edit > Extended Plot Settings:* Allows the user to modify various plot settings. These include:

- *1st Table > Title/XLabel/YLabel:* Sets the text for title and labels of each plot*.*
- *2nd Table > TitleFontSize/TitleFontWeight:* Sets the title font size & weight.
- *2nd Table > XLabelFontSize/XLabelFontWeight:* Sets the x-axis label font size & weight.
- *2nd Table > YLabelFontSize/YLabelFontWeight:* Sets the y-axis label font size & weight.
- *2nd Table > XTickLabelFontSize/XTickLabelFontWeight:* Sets the font size & weight for ticks on the x-axis.
- *2nd Table > YTickLabelFontSize/YTickLabelFontWeight:* Sets the font size & weight for ticks on the y-axis.
- *3rd Table > YScale*: sets the scale for the y-axis.
- *3rd Table > XGrid/XMinorGrid:* Turns on/off the x-axis major and minor grids.
- *3rd Table > YGrid/YMinorGrid:* Turns on/off the x-axis major and minor grids.
- *3rd Table > XLimMode/CustomXLim:* Sets whether the x-axis limits are determined automatically or manually by the user. If “Manual” is selected, the user can change CustomXLim.
- *3rd Table > YLimMode/CustomYLim:* Similar to above for the y-axis.
- *4th Table > LineWidth:* Set the line width for the curves depicted on a plot.
- *4th Table > DataSymbolSize:* Sets the symbol Size for the data points visualized on a plot.
- *4th Table > LegendVisibility:* Turns on/off the visibility of the legend on a plot.
- *4th Table > LegendLocation:* Determines the legend location on a plot.
- *4th Table > LegendFontSize/LegendFontWeight:* Sets the legend font size & weight.

Note that changes in the 1st, 2^nd^, and 3^rd^ tables will immediately apply, whereas changes in the 4th table will be applied after clicking “OK”.

***Simulation Functionality:***

This functionality enables simulation of the model encapsulated in the session file under different parameter values, variants, and dosing regimens. The functionality-specific settings (shown in Fig. 3) include:

*Time*: Determines the time period of the simulation including the time steps at which each output is sampled for visualization. The format of simulation time settings is start time: step: end time. The time units are extracted from the SimBiology^®^ model.

*Parameters*: Includes the parameters that can be altered by the user for each simulation. This section has two views: 1) the default view, in which only the parameter names and values are visible to the user; 2) the extended view, which becomes visible by resizing the section and displays additional features such as parameter units, the lower bounds (LBs) and upper bounds (UBs), and parameter scales. The slider ranges from the lower to upper bound along the chosen scale (linear or log). The bounds for each parameter can be altered. The user can change values by entering the desired value for each parameter in the ‘Value’ box or by use of the sliders. The values should be within the bounds; otherwise, the values will be floored at LBs, or capped at UBs. The user also has the option to restore parameters to their default values. If no variant is selected, the defaults values will be the ones defined in the session file. If there are some variants selected, those values will be the parameter values after they have been overwritten by the variants. The user can also save the current parameter values as a new variant.

*Plot Settings:* This section resides on the top right side and includes the following options:

- *Plot Layout:* Enables the user to select different plot layouts (1x1, 2x1, 2x2 and 2x3) that allow up to six plots.
- *Species Results:* Lists all the species names listed in the session file (these are selected by the modeler to be visible to the user in the session file) and enables assignment of each species output to be displayed to any of the subplots by selection of the plot number; selecting the blank option will remove the output from the plot; multiple outputs can be overlaid on a single plot; the line style for the output can be selected from one of the four choices in the drop-down list. The “Display” column is editable and allows the user to change the species display name shown in the plot legends (the “Species” column is the species name specified in the model and cannot be altered).
- *Dataset (All) and Dataset (Group)*: These enable visualization of the imported data. The tables list the “y-axis” column-headers and the number of groups in the dataset, respectively. Each data output is represented by a symbol and can be assigned to any plot. Groups in the dataset are differentiated by color, which can be altered by selecting the row, right-clicking on the row and clicking “Set Color”. The user can change the display name for the data groups and column headers. The user can also select which groups of data are displayed on the plots.

*Profile Notes:* This section resides below the plots and includes information regarding the most recent simulation runs:

- *Keep Previous Runs*: If checked, the simulation results are retained in memory for the purpose of exporting to Excel files and displaying on the plots. Each simulation run is differentiated by color. Note that when unchecked, the simulation results in memory will be erased once a new simulation is “run”. The user can also delete any simulation from the list of simulation runs.
- *Show and Export columns:* Enables a particular simulation run to be shown on plots and/or exported to an Excel file.
- *Notes & Summary:* This section includes details on all the simulation results saved in memory since selecting the *Keep Previous Runs*. The *Notes* table allows the end-user to enter a description for each simulation run. Selections of specific runs for show or export are made in this table. The user can right-click on each row to delete a simulation run, change the color, and toggle on/off “Show” and “Export” for all simulation runs. When a simulation run is selected, the curves on plots get highlighted (will be shown in thicker lines) and a detailed summary of the simulation run is displayed in the text section, which includes the selected variants, doses and parameter values for that run.

Note that this section is also available for population simulation but not the fitting functionality.

- *Plots Area:* Resides in the middle of the GUI and provides an interactive user-friendly functionality for visualizing simulation results and imported data. The user can right-click on each plot to change the scale of y-axis, “Save Current Axes …” or “Save All Axes in View Individually”. The user can then select any of the following file extensions: .png, .tiff, .eps, and .fig (MATLAB^®^ Figure). The user can also right-click on the legend to hide it. Additional plot properties are available from *Edit > Extended Plot Settings*.

*Running Simulations:* Once the user has selected variants, doses, and adjusted parameter values, the simulation can be executed by *Functionality > Run* or clicking the “Run” button from the toolbar. After execution, the outputs can be displayed on the plots in the middle of the GUI; the user can zoom in, zoom out and pan the plots. Additionally, the “Explore” button enables the user to interact with the plot and read data directly from it by displaying the values of points selected on plotted curves. Once all the desired simulations are run, the user can save plots or export the results as described above.

***Non-Compartmental Analysis (NCA):***

This section resides in the right bottom corner of the GUI and displays the estimated PK parameters for the imported dataset. The NCA outputs and their definitions are as follows:

- *Group:* Shows the identifier for the Group column header in the dataset. As mentioned before, a Group is defined as a number of Subjects receiving the same dose material, through the same dose route, and according to the same dose schedule.
- *Dose Schedule:* Frequency of administration. This could be single or multiple.
- *Administration Route:* Intravenous or extravascular administration.
- *Lambda_Z:* First-order rate constant of elimination.
- *R2:* A coefficient of determination that is a statistical measure of how well the regression line for half-life determination approximates the real data points.
- *adjusted_R2:* A modified version of R2 that has been adjusted for the number of points being included in the regression line for half-life determination.
- *Num_points:* Number of points included in the regression line for half-life determination.
- *AUC_0_last:* Area under the concentration time curve from time zero to the last measured time point.
- *Tlast:* The last measured time point.
- *C_max:* The maximum concentration following the first dose and before the second dose.
- *C_max_Dose:* Cmax normalized by the dose level.
- *T_max:* The time at which Cmax was assessed.
- *MRT:* Mean residence time; the average amount of time the drug remains in a compartment.
- *T_half:* Terminal half-life.
- *AUC_infinity:* Area under the concentration time curve from time zero and extrapolated to infinity.
- *AUC_infinity_dose:* AUC_infinity normalized by the dose level.
- *AUC_extrap_percent:* Percentage of the AUC_infinity value that is from extrapolation.
- *CL:* Clearance
- *DM:* Dose level
- *V_z:* Volume of distribution of the terminal phase.
- *AUMC_0_last:* Area under the moment curve from time zero to the last measured time point.
- *AUMC:* Area under the moment curve from time zero and extrapolated to infinity.
- *AUMC_extrap_percent:* Percentage of the AUMC_infinity value that is from extrapolation.
- *V_ss*: Volume of distribution at steady state.
- *C_0:* Concentration at time zero.
- *AUC_x__y:* Area under the concentration time curve from time x to time y.
- *C_max_x__y:* The maximum concentration between time x and time y.
- *T_max_x__y:* Time at which the maximum concentration between time x and time y occurred.
- *C_avg:* Average concentration at steady state.
- *PTF_Percent:* Percent the concentrations fluctuate between Cmax and Cmin at steady state.
- *Accumulation_Index:* Estimation of drug accumulation at steady state.
- *AUC_Tau:* Area under the concentration time curve at steady state for the dose interval.
- *AUMC_Tau:* Area under the moment curve at steady state for the dose interval.
- *Tlag:* Lag time between the time of dosing and the time of first appearance of measurable concentrations.

***Data Fitting (Parameter Estimation) Functionality:***

The purpose of data fitting is to estimate model parameter values, providing a best fit of model simulation to data. The dataset for the data fitting is imported as described above and must include all the data outputs to be fit as well as the dosing information for each group. The settings/options for data fitting functionality (Fig. S3) include:

*Dose Mapping*: Maps each of the “dosing” column headers in the dataset file to the corresponding species in the model.

*Species mapping:* Maps each of the “y-axis” column headers in the dataset file to the corresponding model species, which will be used in the objective function. If mapping is not specified, the particular column header will be ignored in the objective function.

*Error Model*: Allows selection from a set of four error models for the objective function.

*Pooled Fitting*: If checked, pooled data will be used for parameter estimation; otherwise, parameter estimation will be performed for each group, separately.

*Parameters*: Allows selection of parameters to be estimated with provision of providing initial estimate; if any parameter is not selected, its value will be fixed at the initial value provided in the table. After data fitting is complete, the user can save estimated parameter values as a new variant in the session, enabling use of the parameter estimates for simulation functionality. If pooled fitting is unchecked, the data fitting methods output multiple groups of parameter estimates, all of which can be saved as new variants by the user.

*Execution of Data Fitting*: Once the mappings and parameter selections are made, the fitting can be executed by *Functionality > Run* or clicking the “Run” button from the toolbar. After successful completion of the fitting, the model fits along with the data are shown on the right-hand side plot for visual check of the goodness of fit; the user can save the plot, export the fitting results to an Excel file and/or the fitting summary to a .pdf file (both options are available under *File > Export*), and save the parameter estimates as new variants.

***Population Simulation Functionality:***

In the simulation population view, all options are similar to those of the simulation view with the exception of parameters, where in addition to parameter values, the user also provides CV% for each parameter, which assigns a normal/lognormal (according to the chosen scale) distribution to it (Fig. S4). The purpose of this functionality is to explore the impact of parameter variability on the model outputs of interest. Here, we provide an example of how covariates are handled in gPKPDSim. Let’s assume we are interested in studying the impact of the body weight on clearance. One would define

$${CL}_{i}={CL}_{0}.e^{\eta_{i}}\left( \frac{{BW}_{i}}{\bar{BW}} \right)^{\theta},$$

Where *CL_i_* is the estimated clearance for subject *i*, parameter *η* is a normally distributed random variable and *CL_0_* is the mean clearance for the population of subjects in the study. And *BW_i_* denotes the body weight of subject *i*, $\bar{BW}$ is the median body weight across all subjects and *Θ* is a dimensionless parameter characterizing the magnitude of covariate effect.

Depending on the application of the model, the modeler can include any of the following parameters, *η*, *CL_0_*, *BW_i_*, $\bar{BW}$, and *Θ* in a session file. Within gPKPDSim, the end-user can specify the values in the simulation functionality or determine the parameter values and CV% in the population simulation functionality to determine the value of CL.

The user can also set the number of simulations, i.e. the number of times the parameter distributions are sampled and the model is run. Once the population simulation is complete, the user can plot each species, which will be depicted by the median curve as well as a shaded region corresponding to 5%-95% for that output. Similar to other functionalities, the user can save the plots and/or export the results to an Excel file.

SUPPLEMENTARY INSTRUCTION FOR CASE STUDIES

**Instruction S1: Two-Compartment PK Model**

| *Requirements:*   - Session file: casestudy1_TwoCompPK_template.mat - Dataset: casestudy1_dataset.xlsx - See Table S5 for initial conditions   *Instructions for data import and NCA:*   - Open the session file in gPKPDSim - Import the dataset and note the selections for the column headers from the dataset - Time: Time - Group: Group - y-axis: Conc_ugperml - Dose: DoseIV_ugperkg - Select “Compute NCA” check box and choose the following settings - Serial Analysis with Subgroup: Animal_ID - IV Dose: DoseIV_ugperkg - Concentration: Conc_ugperml - The NCA results for Serial analysis are available in the NCA panel - Export the results to Excel and save the file as casestudy1_NCA_serial.xslx - Open the import dataset dialog and change “Serial” to “Sparse” in the NCA section - Compare the results and save as casestudy1_NCA_sparse.xslx   *Instructions for data fitting:*   - Select the Fitting functionality - Make the following selections for fitting options - Dose mapping table: match DoseIV_ugperkg in the “Dose” column to [PK].[CentralAmt(mcg/kg)] in the “Target Species” column - Species mapping table: match Conc_ugperml in the “Dependent” column to [PK].[CentralConc(mcg/mL)] in the “Target Species” column - Pooled fitting: unchecked - Error model: combined - Select V1, V2, CL, CLd for fitting - Execute (run button on top panel) and export the fitting results to Excel (casestudy1_fitting_combined_unpooled.xlsx) - Select the “pooled fitting” checkbox - Execute (run button on top panel) and export the fitting results to Excel (casestudy1_fitting_combined_pooled.xlsx) - Export fitting summary to PDF (casestudy1_fitting_summary.pdf) - Click Edit > Extended Plot Settings and make appropriate changes on the plot. - Save the figure as casestudy1_fitting.png (Figure 3A) - Save a variant of the fitted parameters using the “Save as variant …” button and name it as “Fit1”   *Instructions for running simulations:*   - Switch to the simulation view - Activate variant “Fit1” by selecting the “Active” checkbox in the table - Select “IV Dose1” and enter the dosing information - Amount = 10000 - Interval = 7 - Repeat count = 3 - Change simulation time settings to 0: 0.1: 28 (start time: step: end time) - Execute the simulation - Change Plot Layout to 2x1 - In the “Species Results” table, assign “CentralConc(mcg/mL)” to plot 1 and “AUC” to plot 2 and change the line style to ‘-’ for both. Also, Change the display column to “Conc (ug/mL) - ” and “AUC - ” - Plot 1: right-click and change the scale to log - Click Edit > Extended Plot Settings, and change the xlabel to Time (days), and title to blank. Change the ylabel to “Central Conc (ug/mL)” and (AUC (ug.day/mL) for plots 1 and 2, respectively. Make other appropriate changes. - Select “Keep previous runs” - Change CL value to 3.45 and execute the simulation (Figs. 4B and 4C) - In the profile notes section, change the description for Run 1 and Run 2 to “CL = 6.89” and “CL = 3.45”, respectively. - Save plot 1 - From the toolbar, select the explore button and read the AUC value of the red curve on plot 2. - Right-click, create a new datatip and read the AUC value on the blue curve - Click Edit > Extended Plot Settings, change the legend position to “southeast” for plot 2 - Save plot 2 - Export the results for the first Simulation Run to an Excel file.   *Instructions for running population analysis:*   - Switch to the population view - Reset the parameters to original values using the “Reset to defaults …” button - Set the CV% to zero for CLd and V2 - Set the CV% to 10% for CL and V1 - Execute the population simulation(Figs. 4D and 4E) - In the profile notes, Change the color of simulation to green - Change the scale to log on plot 1 - Read the AUC value for 5% and 95% boundaries (since this is a random simulation, your numbers may be slightly different than what has been reported in the paper) - Change the description to “10% on V1 and CL” - Click Edit > Extended Plot Settings and make appropriate changes - Save plot 1 and plot 2 - Export the results for the Population Simulation Run to an Excel file. - Save the session file as casestudy1_TwoCompPK_final.mat |
| --- |

**Instruction S2: TMDD Model**

| *Requirements:*   - Session file: casestudy2_TMDD_template.mat - See Table S5 for initial conditions   *Instructions for running simulations:*   - Open the session file in gPKPDSim - Select “IV Dose1” and enter the dosing information - Amount = 1000 - Interval = 28 - Repeat count = 4 - Change simulation time settings to 0: 0.1: 140 (start time: step: end time) - Change target_init to 10 (keep KD at 0.1) - Execute the simulation - Change Plot Layout to 2x2 - Assign “TotalAbConc (mcg/ml)” to plot 1, “TargetFracBound” to plot 2, and “FreeTarget (ng/ml)” and “TotalTarget (ng/ml)” to plot 3. - Change the line style to ‘--’ for “FreeTarget (ng/ml)” and to ‘-’ for the rest of species. - Right-click on plots 1 and 3 to change the scale to log - Click Edit > Extended Plot Settings and change xlabel to “Time (days)”, and title to blank for all plots - Change ylabel to “Total Antibody Conc (ug/mL)”, “Fraction of Target Bound”, and “Free & Total Target (ng/mL)” for plots 1, 2, and 3, respectively. - Set “XLabelFontSize” and “YLabelFontSize” to 20. - Set “XTickLabelFontSize” and “YTickLabelFontSize” to 16. - Set “XTickLabelFontWeight” and “YTickLabelFontWeight” to bold. - Set “LineWidth” to 2. - Select “Keep previous runs” - Change KD to 10 - Execute the simulation - Deselect “IV Dose1”, Select “IV Dose2” and enter the dosing information - Amount = 5000 - Interval = 28 - Repeat count = 4 - Change KD to 0.1 - Execute the simulation - In the profile notes section, change the description for each run to appropriate names. - Click Edit > Extended Plot Settings and change legend location to southeast for plots 2 and 3. - Turn on “XGrid”, “YGrid” and “YMinorGrid” for all plots. - Save plots 1, 2 and 3. - Save the session file as casestudy2_TMDD_final.mat |
| --- |

**Instruction S3: Indirect Response Model**

| *Requirements:*   - Session file: casestudy3_IDR_TwoCompPK_template.mat - See Table S5 for initial conditions   *Instructions for running Simulation 1:*   - Open the session file in gPKPDSim - Select variant “Inhibition - Degradation (R2)” - Select “IV Dose1” and enter the dosing information - Amount = 71.43 - Change simulation time settings to 0: 0.001: 0.15 (start time: step: end time) - Change the parameter values according to column 2 “Simulation 1” in Table 9. - *Note that you need to resize the section containing the list of parameters and increase the UB value on some of the parameters of interest.* - Using the “Save as variant …” button in this section, save the parameter values as a new variant called “Example1”. - Then in the variants table, change the order of “Exmplae1” to 1 and activate the variant. - Execute the simulation - Change Plot Layout to 2x1 - Assign “CentralConc(mcg/mL)” to plot 1, and “Response” to plot 2. Change the scale to Log for plot 1. - Make the appropriate changes to xlabel, ylabel, title, font size, font weight, grids, line width and save the plots by selecting “Save All Axes in View Individually”.   *Instructions for running Simulation 2:*   - Unselect variant “Inhibition - Degradation (R2)” and select variant “Stimulation - Synthesis (R3)” - Unselect “IV Dose1”, select “IV Dose2” and enter the dosing information - Amount = 4285.7 - Change end time to 0.07 - Change the parameter values according to column 3 “Simulation 2” in Table 9 - Save the parameter values as a new variant called “Example2” and change the order to 2. Unselect “Example1” and select “Example2”. - Select “Keep Previous Runs” - Execute the simulation - Unselect the “show” checkbox for Run 1 - Make the appropriate changes to the plots and save them - Save the session file as casestudy3_IDR_TwoCompPK_final.mat |
| --- |

**Instruction S4: Minimal PBPK Model**

| *Requirements:*   - Session file: casestudy4_minPBPK_template.mat - See Table S5 for initial conditions   *Instructions for running simulation with target in the central compartment:*   - Open the session file in gPKPDSim - Select “IV Dose1” and enter the dosing information - Amount = 195000 ug (1300 nmole) - Select “Target_central” variant and notice the changes in the parameter values (CL and CentralTarget0) - Change end time to 1000 (hrs) - Execute the simulation - Assign “ConcCentral_nM” to plot 1 and change the display name to “Conc (nM) -”. - Right-click on plot 1 and change the scale to log - Click Edit > Extended Plot Settings, remove the title and change the xlabel to Time (hrs) and ylabel to “Plasma Conc (nM)” - Select “Keep Previous Runs” - Repeat this for the following dose values - Amount = 97500 ug (650 nmole) - Amount = 39000 ug (260 nmole) - Amount = 3900 ug (26 nmole) - Amount = 390 ug (2.6 nmole) - Change the description for each of the 5 runs to appropriate names (e.g. “1300 nmole (Central)” for the 1st run). - Click Edit > Extended Plot Settings, change the YLimMode to “manual” and CustomYLim to [0.01 1000]. Make other appropriate changes. - Save plot 1   *Instructions for running simulation with target in the leaky compartment:*   - Unselect “Target_central” variant, select “Target_leaky” variant and notice the changes in the parameter values (CL and LeakyTarget0) - Repeat all the 5 dosing scenarios - Change the color for runs 6-10 to Red, Blue, Green, Magenta and Brown. - Change the description for the new set of 5 runs to appropriate names (e.g. “1300 nmole (Leaky)” for the 6th run). - Compare the results for “Central” and “Leaky” targets. - Unselect the “show” checkbox for Runs 1-5. - Save Plot 1.   *Instructions for running simulation with target in the tight compartment:*   - Unselect “Target_leaky” variant, select “Target_tight” variant and notice the changes in the parameter values (CL and TightTarget0) - Repeat all the 5 dosing scenarios - Change the color for runs 11-15 to Red, Blue, Green, Magenta and Brown. - Change the description for the new set of 5 runs to appropriate names (e.g. “1300 nmole (Tight)” for the 11th run). - Compare the results for “Leaky” and “Tight” targets. - Unselect the “show” checkbox for Runs 6-10. - Save Plot 1.   *Instructions for running simulation with no target:*   - Unselect “Target_leaky” variant and notice the changes in the parameter values - Change CLp to 0.007 - Repeat all the 5 dosing scenarios - Change the color for runs 16-20 to Red, Blue, Green, Magenta and Brown. - Change the description for the new set of 5 runs to appropriate names (e.g. “1300 nmole (No Target)” for the 16th run). - Compare the results for “Tight” target and “No Target” - Unselect the “show” checkbox for Runs 11-15. - Save Plot 1. - Save the session file as casestudy4_minPBPK_final.mat |
| --- |

SUPPLEMENTARY FOLDERS

**Folder 1) Case Study #1: Two-compartment antibody PK model**

*casestudy1_dataset.xlsx:* The dataset containing PK samples for IV dosing at two dose levels

*casestudy1_TwoCompPK.sbproj:* The SimBiology^®^ file for the two-compartment PK model

*casestudy1_TwoCompPK_equations.pdf:* Contains the equations, species, parameters and their units in the SimBiology^®^ model

*casestudy1_TwoCompPK_template.mat:* Template Session file containing the SimBiology^®^ file

*casestudy1_NCA_serial.xlsx:* NCA Results for serial sampling scheme

*casestudy1_NCA_sparse.xlsx:* NCA Results for the sparse sampling scheme

*casestudy1_winnonlin_NCA.xlsx:* NCA results from gPKPDSim compared with the results from WinNonlin^®^

*casestudy1_fitting_combined_pooled.xlsx:* PK parameters estimated using the combined error mode for pooled data fitting

*casestudy1_fitting_combined_unpooled.xlsx:* PK parameters estimated using the combined error mode for un-pooled data fitting

*casestudy1_fitting_summary_combined_pooled.pdf:* The data fitting summary report containing information on the quality of parameter estimation and the visual predictive checks

*casestudy1_fitting.png:* The plot showing the models fits to the data for the pooled data fitting

*casestudy1_sim_results.xlsx:* Results of simulation runs

*casestudy1_sim_conc.png:* The plot showing the simulated PK profiles

*casestudy1_sim_AUC.png:* The plot showing the simulated AUC profiles

*casestudy1_popsim_results.xlsx:* Results of population simulation runs

*casestudy1_pop_AUC.png:* The plot showing the median, 5% and 95% boundaries on the simulated PK profiles

*casestudy1_pop_conc.png:* The plot showing the median, 5% and 95% boundaries on the simulated AUC profiles

*casestudy1_TwoCompPK_final.mat:* The Session file with all the data and analyses saved in it.

**Folder 2) Case Study #2: Target-mediated drug disposition model**

*casestudy2_TMDD.sbproj:* The SimBiology^®^ file for the TMDD model

*casestudy2_TMDD_equations.pdf:* Contains the equations, species, parameters and their units in the SimBiology^®^ model

*casestudy2_TMDD_template.mat:* Template Session file containing the SimBiology^®^ file

*casestudy2_fig_conc.png:* The plot showing the simulated PK profiles

*casestudy2_fig_target.png:* The plot showing the simulated profiles for free and total target concentration

*casestudy2_fig_fracbound.png:* The plot showing the simulated profiles for fraction of target bound

*casestudy2_TMDD_final.mat*: The Session file with all the data and analyses saved in it.

**Folder 3) Case Study #3: Physiologic indirect response model**

*casestudy3_IDR_TwoCompPK.sbproj:* The SimBiology^®^ file for the indirect response model

*casestudy3_IDR_TwoCompPK_equations.pdf:* Contains the equations, species, parameters and their units in the SimBiology^®^ model

*casestudy3_IDR_TwoCompPK_template.mat:* Template Session file containing the SimBiology^®^ file

*casestudy3_Example1_PK.png:* The plot showing the simulated PK profiles for Example 1

*casestudy3_Example1_PD.png:* The plot showing the simulated PD profiles for Example 1

*casestudy3_Example2_PK.png:* The plot showing the simulated PK profiles for Example 2

*casestudy3_Example2_PD.png:* The plot showing the simulated PD profiles for Example 2

*casestudy3_IDR_TwoCompPK_final.mat:* The Session file with all the data and analyses saved in it.

**Folder 4) Case Study #4: Minimal physiologically based pharmacokinetic (PBPK) model**

*casestudy4_minPBPK.sbproj*: The SimBiology^®^ file for the minimal PBPK model

*casestudy4_minPBPK_equations.pdf:* Contains the equations, species, parameters and their units in the SimBiology^®^ model

*casestudy4_minPBPK_template.mat*: Template Session file containing the SimBiology^®^ file

*casestudy4_Fig_Central.png:* The plot showing the simulated PK profiles based on target-mediated drug disposition in central compartment

*casestudy4_Fig_Leaky.png:* The plot showing the simulated PK profiles based on target-mediated drug disposition in leaky tissues

*casestudy4_Fig_Tight.png:* The plot showing the simulated PK profiles based on target-mediated drug disposition in tight tissues

*casestudy4_Fig_NoTarget.png:* The plot showing the simulated PK profiles based on no target in central compartment

*casestudy4_minPBPK_final.mat*: The Session file with all the data and analyses saved in it.

**Folder 5) NCA Results for Sample Datasets**

The NCA results from gPKPDSim and WinNonlin^®^ are compared for thirteen datasets. In our comparison, we use “Linear Trapezoidal Linear/Log Interpolation” calculation method in WinNonlin^®^. The maximum absolute difference in all cases is less than 0.1%, when the number of data points used for calculation of parameters is matched (see Table S4).

**Folder 6) How to Install the App in MATLAB**^®^
