# Supplementary material for: gPKPDSim: a SimBiology®-based GUI application for PKPD modeling in drug development
Source: J Pharmacokinet Pharmacodyn. 2018 Jan 4;45(2):259–75. doi: 10.1007/s10928-017-9562-9 (PMC5845055; doi:10.1007/s10928-017-9562-9)
Supplement: Supplementary file 2 — Electronic supplementary material 2 (ZIP 7898 kb) [file 10928_2017_9562_MOESM2_ESM.zip › Supplementary Material/1) Case Study 1/casestudy1_fitting_summary_combined_pooled.pdf]

Estimated Parameter Values

#	Name	EstimatedValue	StandardError
1	V1	49.15021602	2.5736199
2	V2	34.60691983	4.5587834
3	CL	6.88919382	0.52910269
4	CLd	45.50202536	8.14558963

Estimation Statistics

Property	Value
Error Model Parameters	a = 2.84301756 b = 0.15654477

Warning: An unexpected error occurred while validating the mex setup.
Please contact Technical Support if this problem persists.
Warning: A compiler supporting MATLAB Coder functionality and setup
(by
running "mex -setup") to take advantage of accelerated simulations. <a
href="matlab: warning('off',
'SimBiology:CodeGeneration:InvalidMexCompilerFitting')">Click here
if
you do not want to see this message again.

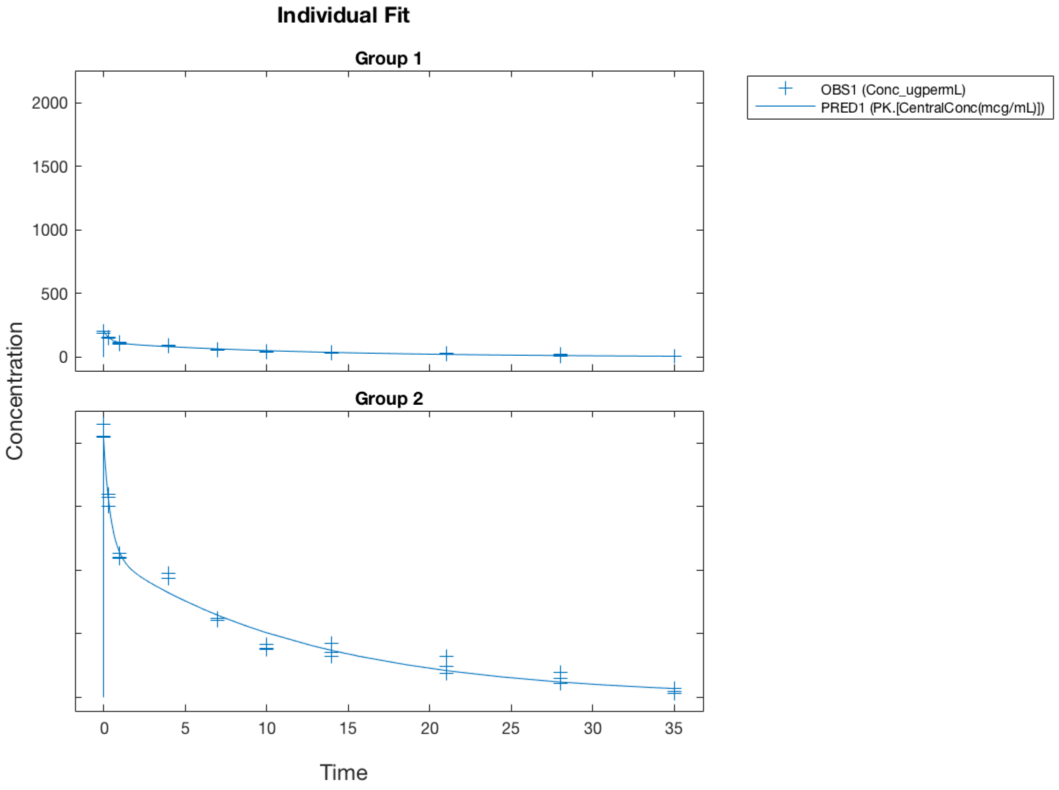

Individual Fit
Group 1
Group 2
Concentration
Time
0
500
1000
1500
2000
0
5
10
15
20
25
30
35
OBS1 (Conc_ugpermL)
PRED1 (PK.[CentralConc(mcg/mL)])

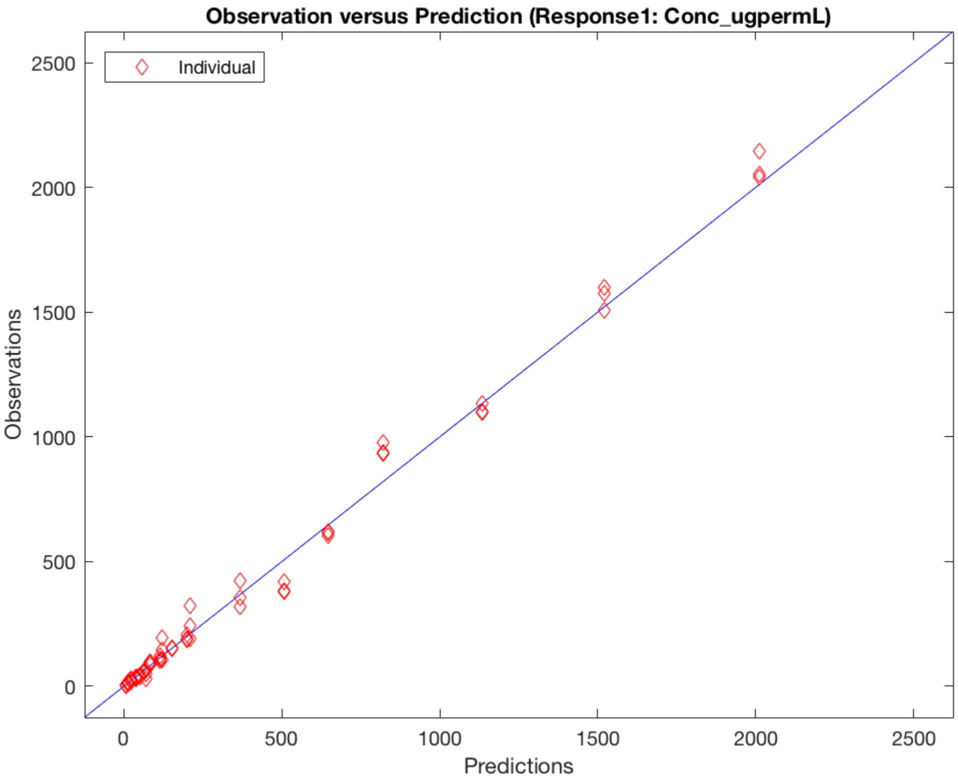
Observation versus Prediction (Response1: Conc_ugpermL)
Individual
Observations
Predictions
0
500
1000
1500
2000
2500

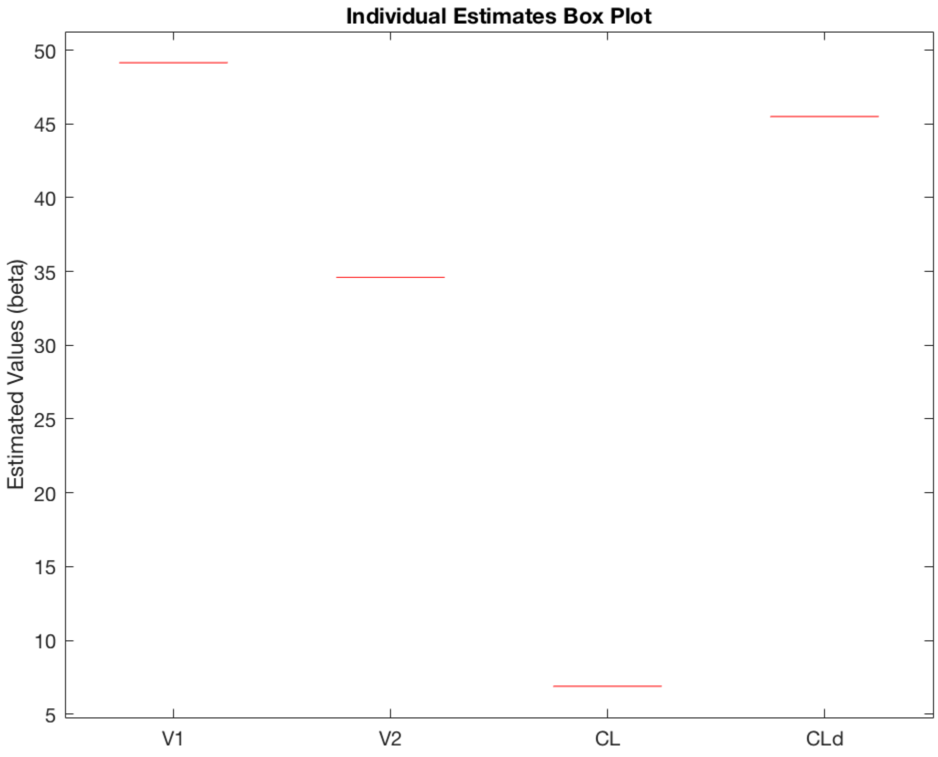
Individual Estimates Box Plot
Estimated Values (beta)
5
10
15
20
25
30
35
40
45
50
V1
V2
CL
CLd

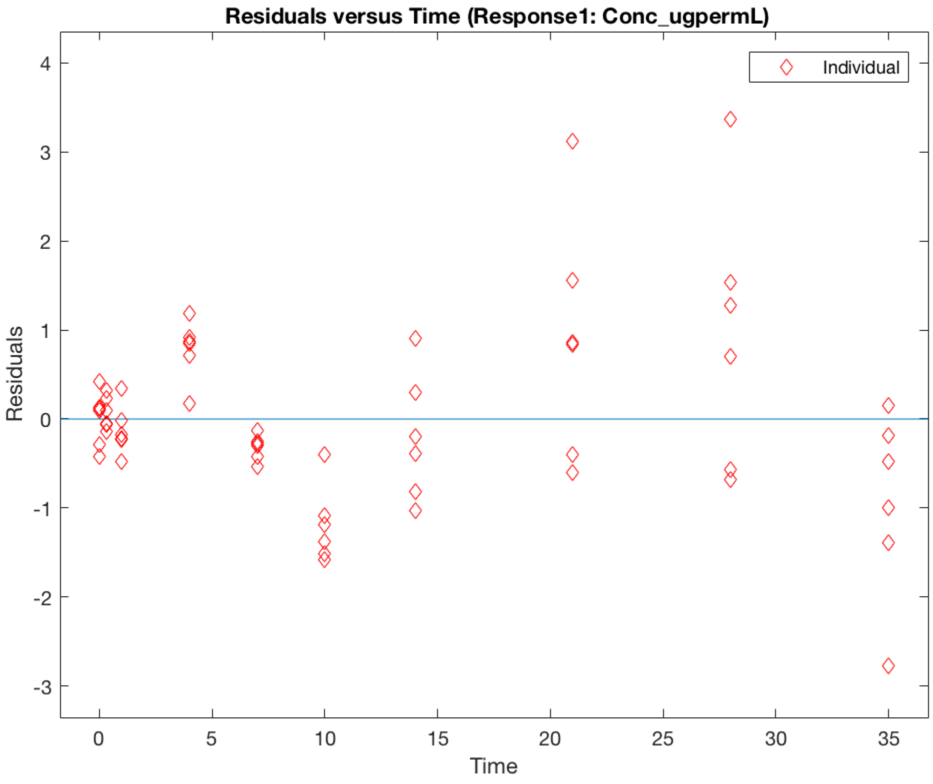
Residuals versus Time (Response1: Conc_ugpermL)
Individual
Residuals
Time
4
3
2
1
0
-1
-2
-3
0
5
10
15
20
25
30
35

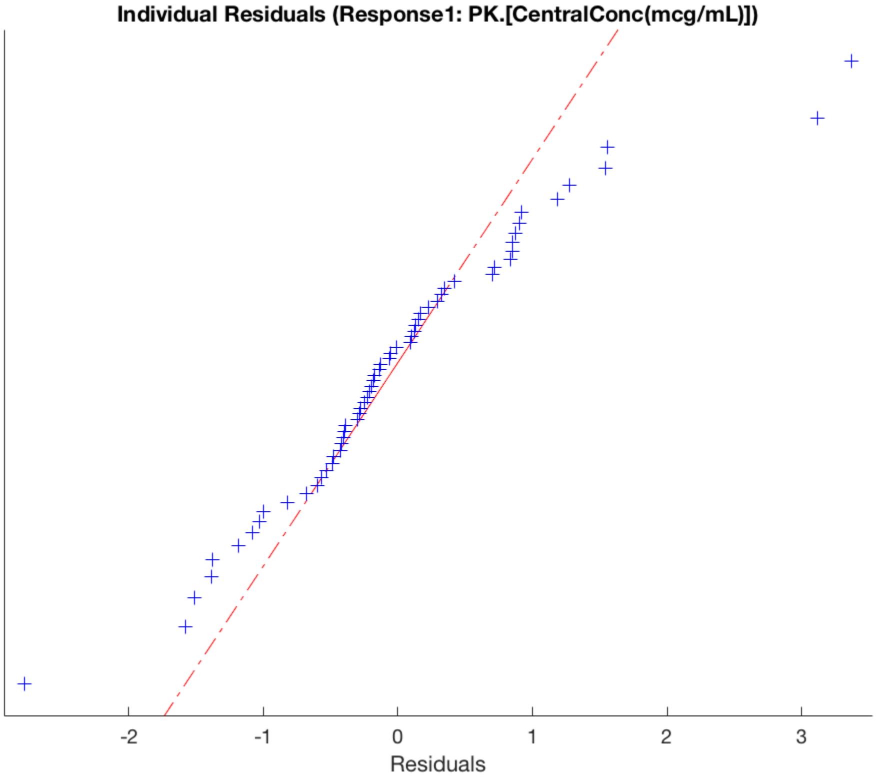
Individual Residuals (Response1: PK.[CentralConc(mcg/mL)])
Residuals
-2
-1
0
1
2
3

Published with MATLAB® R2016b
