# Supplementary material for: gPKPDSim: a SimBiology®-based GUI application for PKPD modeling in drug development
Source: J Pharmacokinet Pharmacodyn. 2018 Jan 4;45(2):259–75. doi: 10.1007/s10928-017-9562-9 (PMC5845055; doi:10.1007/s10928-017-9562-9)
Supplement: Supplementary file 2 — Electronic supplementary material 2 (ZIP 7898 kb) [file 10928_2017_9562_MOESM2_ESM.zip › Supplementary Material/1) Case Study 1/casestudy1_TwoCompPK_equations.pdf]

SimBiology Model: two compartment PK

Repeated Assignments:

1. [CentralConc(mcg/mL)] = [CentralAmt(mcg/kg)]/V1
2. [PeriConc(mcg/mL)] = [PeriAmt(mcg/kg)]/V2
3. log10Conc = log10([CentralConc(mcg/mL)]+1e-6)

ODEs:

1. d([CentralAmt(mcg/kg)])/dt = 1/PK*(-(CLd*([CentralConc(mcg/mL)]-[PeriConc(mcg/mL)])) - (CL*[CentralConc(mcg/mL)]) - (Vm*[CentralConc(mcg/mL)]/(Km+[CentralConc(mcg/mL)])) + ((kabs*fbio*[SCdepot(mcg/kg)])*PK))
2. d([PeriAmt(mcg/kg)])/dt = 1/PK*((CLd*([CentralConc(mcg/mL)]-[PeriConc(mcg/mL)])))
3. d([SCdepot(mcg/kg)])/dt = 1/PK*(-((kabs*fbio*[SCdepot(mcg/kg)])*PK) - ((kabs*(1-fbio)*[SCdepot(mcg/kg)])*PK))
4. d(AUC)/dt = 1/PK*(([CentralConc(mcg/mL)]))
5. d(Cmax)/dt = 1/PK*((50*([CentralConc(mcg/mL)]-Cmax)*([CentralConc(mcg/mL)]>Cmax)))

Name	Type	Scope	Initial Value	Units
PK	compartment	two compartment PK	1.0	
AUC	species	PK	0.0	
CentralAmt(mcg/kg)	species	PK	0.0	
CentralConc(mcg/mL)	species	PK	0.0	
Cmax	species	PK	0.0	
log10Conc	species	PK	-6.0	
PeriAmt(mcg/kg)	species	PK	0.0	
PeriConc(mcg/mL)	species	PK	0.0	
SCdepot(mcg/kg)	species	PK	0.0	
CL	parameter	two compartment PK	5.0	milliliter/day/kilogram
CLd	parameter	two compartment PK	10.0	milliliter/day/kilogram
fbio	parameter	two compartment PK	0.7	fraction
kabs	parameter	two compartment PK	10.0	1/day
Km	parameter	two compartment PK	5.0	microgram/milliliter
V1	parameter	two compartment PK	40.0	milliliter/kilogram
V2	parameter	two compartment PK	40.0	milliliter/kilogram
Vm	parameter	two compartment PK	0.0	microgram/day/kilogram
