# Supplementary material for: gPKPDSim: a SimBiology®-based GUI application for PKPD modeling in drug development
Source: J Pharmacokinet Pharmacodyn. 2018 Jan 4;45(2):259–75. doi: 10.1007/s10928-017-9562-9 (PMC5845055; doi:10.1007/s10928-017-9562-9)
Supplement: Supplementary file 2 — Electronic supplementary material 2 (ZIP 7898 kb) [file 10928_2017_9562_MOESM2_ESM.zip › Supplementary Material/2) Case Study 2/casestudy2_TMDD_equations.pdf]

**SimBiology Model: TMDDmodel**

**Repeated Assignments:**

1. [UnboundAbConc (mcg/ml)] = [UnboundAbAmt (mcg/kg)]/V1
2. [PeriConc (mcg/ml)] = [PeriAbAmt (mcg/kg)]/V2
3. [UnboundAb (nM)] = [UnboundAbConc (mcg/ml)] * 1e3/MWab
4. [TotalAbConc (mcg/ml)] = ([UnboundAb (nM)]+[Complex (nM)])*MWab/1e3
5. [FreeTarget (ng/ml)] = [FreeTarget (nM)]*MWtarget
6. [TotalTarget (ng/ml)] = [FreeTarget (ng/ml)]+[Complex (nM)]*MWtarget
7. TargetFracBound = min(1,max(0,(1-[FreeTarget (nM)]/target_init)))

**ODEs:**

1. d([UnboundAbAmt (mcg/kg)])/dt = 1/[TMDD model]*(-(CLd*([UnboundAbConc (mcg/ml)]-[PeriConc (mcg/ml)])) - (CL*[UnboundAbConc (mcg/ml)]) + ((fbio*kabs*[SCdepot (mcg/kg)])*[TMDD model]) - ((kon*[UnboundAb (nM)]*[FreeTarget (nM)]-kon*KD*[Complex (nM)])*MWab/1e3*V1))
2. d([PeriAbAmt (mcg/kg)])/dt = 1/[TMDD model]*((CLd*([UnboundAbConc (mcg/ml)]-[PeriConc (mcg/ml)])))
3. d([SCdepot (mcg/kg)])/dt = 1/[TMDD model]*(-((fbio*kabs*[SCdepot (mcg/kg)])*[TMDD model]) - (((1-fbio)*kabs*[SCdepot (mcg/kg)])*[TMDD model]))
4. d([FreeTarget (nM)])/dt = 1/[TMDD model]*(-(((kon*[UnboundAb (nM)]*[FreeTarget (nM)]-kon*KD*[Complex (nM)]))*[TMDD model]) + ((log(2)/target_thalf)*target_init) - ((log(2)/target_thalf)*[FreeTarget (nM)]))
5. d([Complex (nM)])/dt = 1/[TMDD model]*((((kon*[UnboundAb (nM)]*[FreeTarget (nM)]-kon*KD*[Complex (nM)]))*[TMDD model]) - ((complCLfactor*(CL/V1*[Complex (nM)]))*[TMDD model]))

| Name | Type | Scope | Initial Value | Units |
|---|---|---|---|---|
| TMDD model | compartment | TMDDmodel | 1.0 | |
| Complex (nM) | species | TMDD model | 0.0 | |
| FreeTarget (ng/ml) | species | TMDD model | 0.0 | |
| FreeTarget (nM) | species | TMDD model | 0.0 | |
| PeriAbAmt (mcg/kg) | species | TMDD model | 0.0 | |
| PeriConc (mcg/ml) | species | TMDD model | 0.0 | |
| SCdepot (mcg/kg) | species | TMDD model | 0.0 | |
| TargetFracBound | species | TMDD model | 0.0 | |
| TotalAbConc (mcg/ml) | species | TMDD model | 0.0 | |
| TotalTarget (ng/ml) | species | TMDD model | 0.0 | |
| UnboundAb (nM) | species | TMDD model | 0.0 | |
| UnboundAbAmt (mcg/kg) | species | TMDD model | 0.0 | |
| UnboundAbConc (mcg/ml) | species | TMDD model | 0.0 | |
| CL | parameter | TMDDmodel | 5.0 | milliliter/day/kilogram |
| CLd | parameter | TMDDmodel | 10.0 | milliliter/day/kilogram |
| complCLfactor | parameter | TMDDmodel | 1.0 | fold |
| fbio | parameter | TMDDmodel | 0.7 | fraction |
| kabs | parameter | TMDDmodel | 0.2 | 1/day |
| KD | parameter | TMDDmodel | 0.1 | nM |
| kon | parameter | TMDDmodel | 400.0 | 1/nM/day |
| MWab | parameter | TMDDmodel | 150.0 | microgram/nanomolarity |
| MWtarget | parameter | TMDDmodel | 38.0 | microgram/nanomolarity |
| target_init | parameter | TMDDmodel | 0.0 | nM |
| target_thalf | parameter | TMDDmodel | 1.0 | day |
| V1 | parameter | TMDDmodel | 40.0 | milliliter/kilogram |
| V2 | parameter | TMDDmodel | 40.0 | milliliter/kilogram |
