# Supplementary material for: gPKPDSim: a SimBiology®-based GUI application for PKPD modeling in drug development
Source: J Pharmacokinet Pharmacodyn. 2018 Jan 4;45(2):259–75. doi: 10.1007/s10928-017-9562-9 (PMC5845055; doi:10.1007/s10928-017-9562-9)
Supplement: Supplementary file 2 — Electronic supplementary material 2 (ZIP 7898 kb) [file 10928_2017_9562_MOESM2_ESM.zip › Supplementary Material/3) Case Study 3/casestudy3_IDR_TwoCompPK_equations.pdf]

SimBiology Model: IDR-TwoCompPK

Repeated Assignments:

1. [CentralConc(mcg/mL)] = [CentralAmt(mcg/kg)]/V1
2. [PeriConc(mcg/mL)] = [PeriAmt(mcg/kg)]/V2
3. PK.Response = (SimNo == 1) * [R1 (Inhibition-kin)] + (SimNo == 2) * [R2 (Inhibition-kout)] + (SimNo == 3) * [R3 (Stimulation-kin)] + (SimNo == 4) * [R4 (Stimulation-kout)]

ODEs:

1. d([CentralAmt(mcg/kg)])/dt = 1/PK*(-(CLd*([CentralConc(mcg/mL)]-[PeriConc(mcg/mL)])) - (CL*[CentralConc(mcg/mL)]) - (Vm*[CentralConc(mcg/mL)]/(Km+[CentralConc(mcg/mL)])) + ((kabs*fbio*[SCdepot(mcg/kg)])*PK))
2. d([PeriAmt(mcg/kg)])/dt = 1/PK*((CLd*([CentralConc(mcg/mL)]-[PeriConc(mcg/mL)])))
3. d([SCdepot(mcg/kg)])/dt = 1/PK*(-((kabs*fbio*[SCdepot(mcg/kg)])*PK) - ((kabs*(1-fbio)*[SCdepot(mcg/kg)])*PK))
4. d(AUC)/dt = 1/PK*(([CentralConc(mcg/mL)]))
5. d([R1 (Inhibition-kin)])/dt = 1/PK*((kin*(1-[CentralConc(mcg/mL)]/(IC50+[CentralConc(mcg/mL)]))) - (kout*[R1 (Inhibition-kin)]))
6. d([R2 (Inhibition-kout)])/dt = 1/PK*(-(kout*(1-[CentralConc(mcg/mL)]/(IC50+[CentralConc(mcg/mL)]))*[R2 (Inhibition-kout)]) + (kin))
7. d([R3 (Stimulation-kin)])/dt = 1/PK*(-(kout*[R3 (Stimulation-kin)]) + (kin*(1+Emax*[CentralConc(mcg/mL)]/(EC50+[CentralConc (mcg/mL)]))))
8. d([R4 (Stimulation-kout)])/dt = 1/PK*(-(kout*(1+Emax*[CentralConc(mcg/mL)]/(EC50+[CentralConc(mcg/mL)]))*[R4 (Stimulation-kout)]) + (kin))

Name	Type	Scope	Initial Value	Units
PK	compartment	IDR-TwoCompPK	1.0	
AUC	species	PK	0.0	
CentralAmt(mcg/kg)	species	PK	0.0	
CentralConc(mcg/mL)	species	PK	0.0	
PeriAmt(mcg/kg)	species	PK	0.0	
PeriConc(mcg/mL)	species	PK	0.0	
R1 (Inhibition-kin)	species	PK	1.0	
R2 (Inhibition-kout)	species	PK	1.0	
R3 (Stimulation-kin)	species	PK	1.0	
R4 (Stimulation-kout)	species	PK	1.0	
Response	species	PK	0.0	
SCdepot(mcg/kg)	species	PK	0.0	
CL	parameter	IDR-TwoCompPK	5.0	milliliter/day/kilogram
CLd	parameter	IDR-TwoCompPK	10.0	milliliter/day/kilogram
EC50	parameter	IDR-TwoCompPK	1.0	microgram/milliliter
Emax	parameter	IDR-TwoCompPK	1.0	
fbio	parameter	IDR-TwoCompPK	0.7	fraction
IC50	parameter	IDR-TwoCompPK	1.0	microgram/milliliter
kabs	parameter	IDR-TwoCompPK	10.0	1/day
kin	parameter	IDR-TwoCompPK	1.0	
Km	parameter	IDR-TwoCompPK	5.0	microgram/milliliter
kout	parameter	IDR-TwoCompPK	1.0	
SimNo	parameter	IDR-TwoCompPK	0.0	
V1	parameter	IDR-TwoCompPK	40.0	milliliter/kilogram
V2	parameter	IDR-TwoCompPK	40.0	milliliter/kilogram
Vm	parameter	IDR-TwoCompPK	0.0	microgram/day/kilogram
