# Supplementary material for: gPKPDSim: a SimBiology®-based GUI application for PKPD modeling in drug development
Source: J Pharmacokinet Pharmacodyn. 2018 Jan 4;45(2):259–75. doi: 10.1007/s10928-017-9562-9 (PMC5845055; doi:10.1007/s10928-017-9562-9)
Supplement: Supplementary file 2 — Electronic supplementary material 2 (ZIP 7898 kb) [file 10928_2017_9562_MOESM2_ESM.zip › Supplementary Material/4) Case Study 4/casestudy4_minPBPK_equations.pdf]

SimBiology Model: minPBPK

Repeated Assignments:

1. min_PBPK.ConcCentral = AmtCentral/Vplasma
2. min_PBPK.ConcCentral_nM = ConcCentral*(1/MWab)
3. min_PBPK.ConcTight = AmtTight/Vtight
4. min_PBPK.ConcTight_nM = ConcTight*(1/MWab)
5. min_PBPK.ConcLeaky = AmtLeaky/Vleaky
6. min_PBPK.ConcLeaky_nM = ConcLeaky*(1/MWab)
7. min_PBPK.Target_Tot = TargetCentral + ComplexCentral

ODEs:

1. d(AmtCentral)/dt = -((kon*ConcCentral_nM*TargetCentral-kon*KD*ComplexCentral)*Vplasma*MWab) - ((1-sig_tight)*L_tight*ConcCentral) - ((1-sig_leaky)*L_leaky*ConcCentral) + (L*AmtLymph/Vlymph) - (CLp*ConcCentral)
2. d(AmtTight)/dt = ((1-sig_tight)*L_tight*ConcCentral) - ((1-sig_lymph)*L_tight*ConcTight) - ((kon*ConcTight_nM*TargetTight-kon*KD*ComplexTight)*Vtight*MWab)
3. d(AmtLeaky)/dt = ((1-sig_leaky)*L_leaky*ConcCentral) - ((1-sig_lymph)*L_leaky*ConcLeaky) - ((kon*ConcLeaky_nM*TargetLeaky-kon*KD*ComplexLeaky)*Vleaky*MWab)
4. d(AmtLymph)/dt = ((1-sig_lymph)*L_tight*ConcTight) + ((1-sig_lymph)*L_leaky*ConcLeaky) - (L*AmtLymph/Vlymph)
5. d(TargetCentral)/dt = 1/min_PBPK*(((ksyn_central)*min_PBPK) - ((kdeg_central*TargetCentral)*min_PBPK) - (kon*ConcCentral_nM*TargetCentral-kon*KD*ComplexCentral))
6. d(TargetLeaky)/dt = 1/min_PBPK*(((ksyn_leaky)*min_PBPK) - ((kdeg_leaky*TargetLeaky)*min_PBPK) - (kon*ConcLeaky_nM*TargetLeaky-kon*KD*ComplexLeaky))
7. d(TargetTight)/dt = 1/min_PBPK*(((ksyn_tight)*min_PBPK) - ((kdeg_tight*TargetTight)*min_PBPK) - (kon*ConcTight_nM*TargetTight-kon*KD*ComplexTight))
8. d(ComplexCentral)/dt = 1/min_PBPK*((kon*ConcCentral_nM*TargetCentral-kon*KD*ComplexCentral) - ((kint*ComplexCentral)*min_PBPK))
9. d(ComplexLeaky)/dt = 1/min_PBPK*((kon*ConcLeaky_nM*TargetLeaky-kon*KD*ComplexLeaky) - ((kint*ComplexLeaky)*min_PBPK))
10. d(ComplexTight)/dt = 1/min_PBPK*((kon*ConcTight_nM*TargetTight-kon*KD*ComplexTight) - ((kint*ComplexTight)*min_PBPK))

Name	Type	Scope	Initial Value	Units
min_PBPK	compartment	minPBPK	1.0	
AmtCentral	species	min_PBPK	0.0	microgram
AmtLeaky	species	min_PBPK	0.0	microgram
AmtLymph	species	min_PBPK	0.0	microgram
AmtTight	species	min_PBPK	0.0	microgram
ComplexCentral	species	min_PBPK	0.0	nM
ComplexLeaky	species	min_PBPK	0.0	nM
ComplexTight	species	min_PBPK	0.0	nM
ConcCentral	species	min_PBPK	0.0	microgram/liter
ConcCentral_nM	species	min_PBPK	0.0	nM
ConcLeaky	species	min_PBPK	0.0	microgram/liter
ConcLeaky_nM	species	min_PBPK	0.0	nM
ConcTight	species	min_PBPK	0.0	microgram/liter
ConcTight_nM	species	min_PBPK	0.0	nM
Target_Tot	species	min_PBPK	10.0	nM
TargetCentral	species	min_PBPK	10.0	nM
TargetLeaky	species	min_PBPK	0.0	nM
TargetTight	species	min_PBPK	0.0	nM
CentralTarget0	parameter	minPBPK	10.0	nM
CLp	parameter	minPBPK	0.07	liter/hour
KD	parameter	minPBPK	0.01	nM
kdeg_central	parameter	minPBPK	0.01	1/hour
kdeg_leaky	parameter	minPBPK	0.01	1/hour
kdeg_tight	parameter	minPBPK	0.01	1/hour
kint	parameter	minPBPK	0.03	1/hour
kon	parameter	minPBPK	0.1	1/nM*hour
ksyn_central	parameter	minPBPK	0.1	nM/hour
ksyn_leaky	parameter	minPBPK	0.0	nM/hour
ksyn_tight	parameter	minPBPK	0.0	nM/hour
L	parameter	minPBPK	0.121	liter/hour
L_leaky	parameter	minPBPK	0.081	liter/hour
L_tight	parameter	minPBPK	0.04	liter/hour
LeakyTarget0	parameter	minPBPK	0.0	nM
MWab	parameter	minPBPK	150.0	microgram/nanomolarity
sig_leaky	parameter	minPBPK	0.687	
sig_lymph	parameter	minPBPK	0.2	
sig_tight	parameter	minPBPK	0.945	
TightTarget0	parameter	minPBPK	0.0	nM
Vleaky	parameter	minPBPK	4.368	liter
Vlymph	parameter	minPBPK	5.2	liter
Vplasma	parameter	minPBPK	2.6	liter
Vtight	parameter	minPBPK	8.112	liter
