# Supplementary material for: gPKPDSim: a SimBiology®-based GUI application for PKPD modeling in drug development
Source: J Pharmacokinet Pharmacodyn. 2018 Jan 4;45(2):259–75. doi: 10.1007/s10928-017-9562-9 (PMC5845055; doi:10.1007/s10928-017-9562-9)
Supplement: Supplementary file 2 — Electronic supplementary material 2 (ZIP 7898 kb) [file 10928_2017_9562_MOESM2_ESM.zip › Supplementary Material/6) Instructions /Instructions to install gPKPDSim.docx]

**App installation:**

1- Open Matlab 2016a/2016b

2- Install the GUI Layout Toolbox (''GUI Layout Toolbox 2.3.1.mltbx")

- https://www.mathworks.com/matlabcentral/fileexchange/47982-gui-layout-toolbox

3- Install the NCA Toolbox (''SimBiologyNCA.mltbx")

- https://www.mathworks.com/matlabcentral/fileexchange/65303-simbiologynca

4- Install "gPKPDSimToolbox.mltbx"

- https://www.mathworks.com/matlabcentral/fileexchange/65399-gpkpdsimtoolbox

5- Restart Matlab

**App re-installation:**

1- Uninstall gPKPDSim from the App Toolstrip

2- Reinstall gPKPDSim from Google Drive

3- Restart MATLAB

4- Ensure the MATLAB path is clean via the pathtool command
