# Supplementary figures and images for: gPKPDSim: a SimBiology®-based GUI application for PKPD modeling in drug development
Source: J Pharmacokinet Pharmacodyn. 2018 Jan 4;45(2):259–75. doi: 10.1007/s10928-017-9562-9 (PMC5845055; doi:10.1007/s10928-017-9562-9)

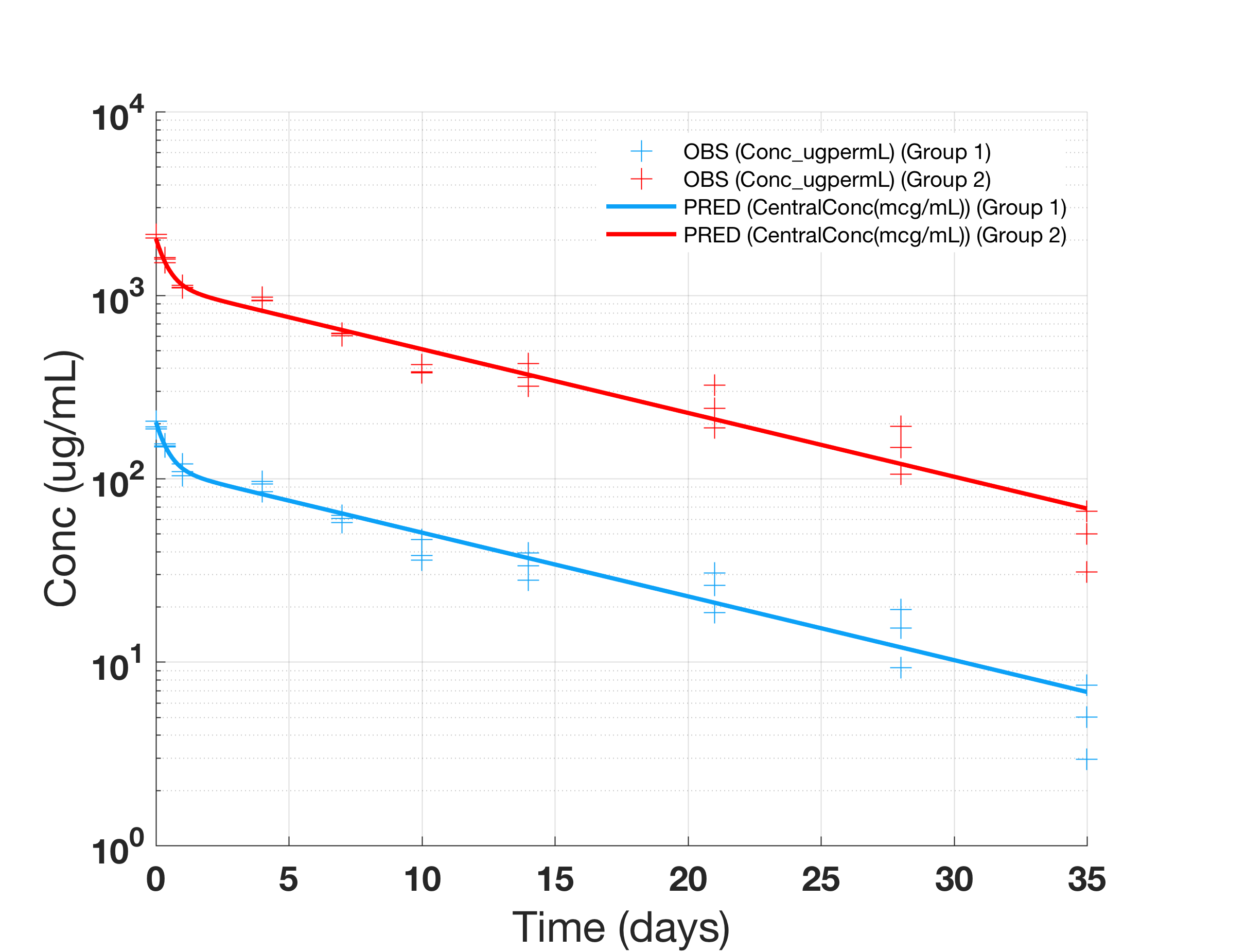

Supplement: Supplementary file 2 — Electronic supplementary material 2 (ZIP 7898 kb) [file 10928_2017_9562_MOESM2_ESM.zip › Supplementary Material/1) Case Study 1/casestudy1_fitting.png]

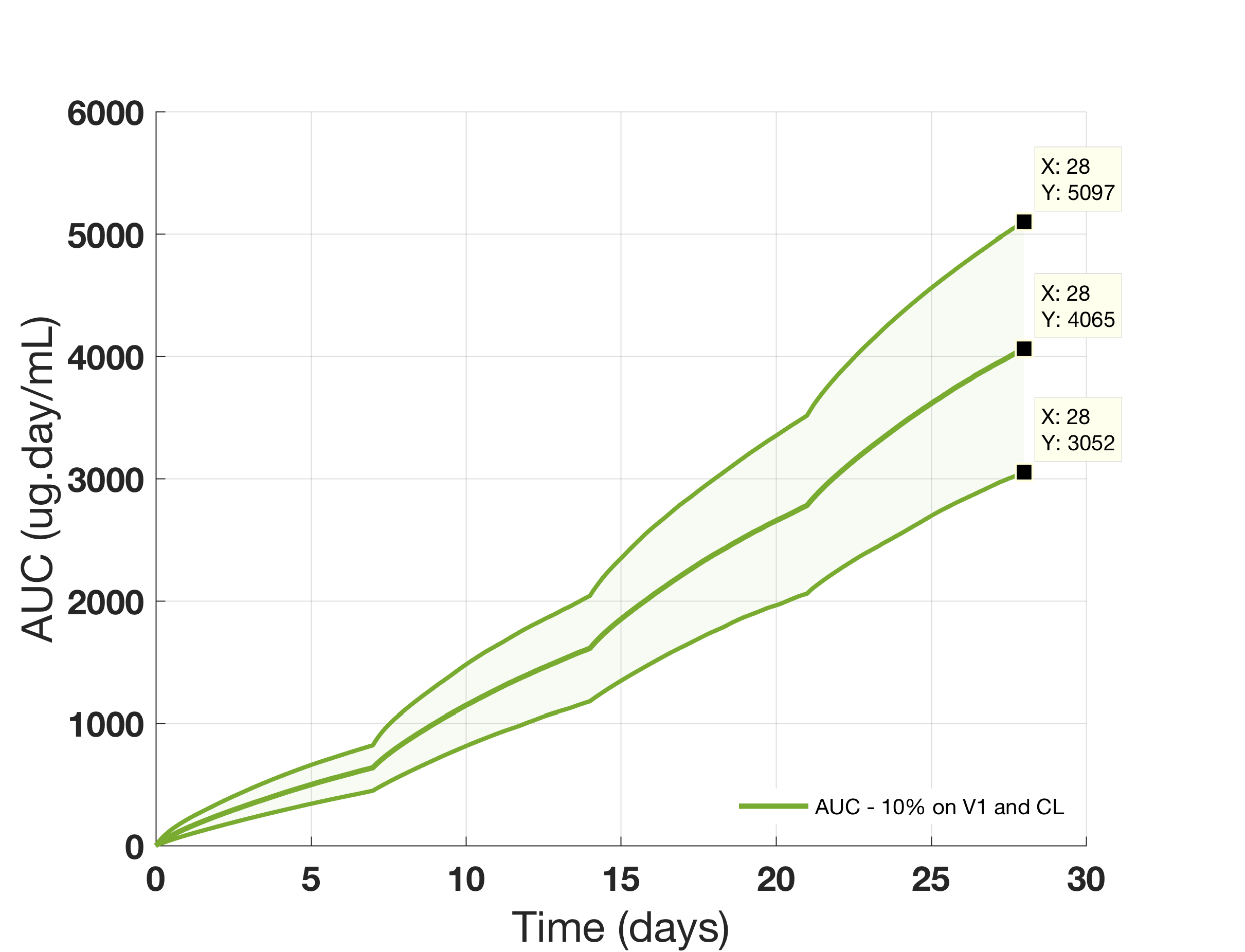

Supplement: Supplementary file 2 — Electronic supplementary material 2 (ZIP 7898 kb) [file 10928_2017_9562_MOESM2_ESM.zip › Supplementary Material/1) Case Study 1/casestudy1_pop_AUC.png]

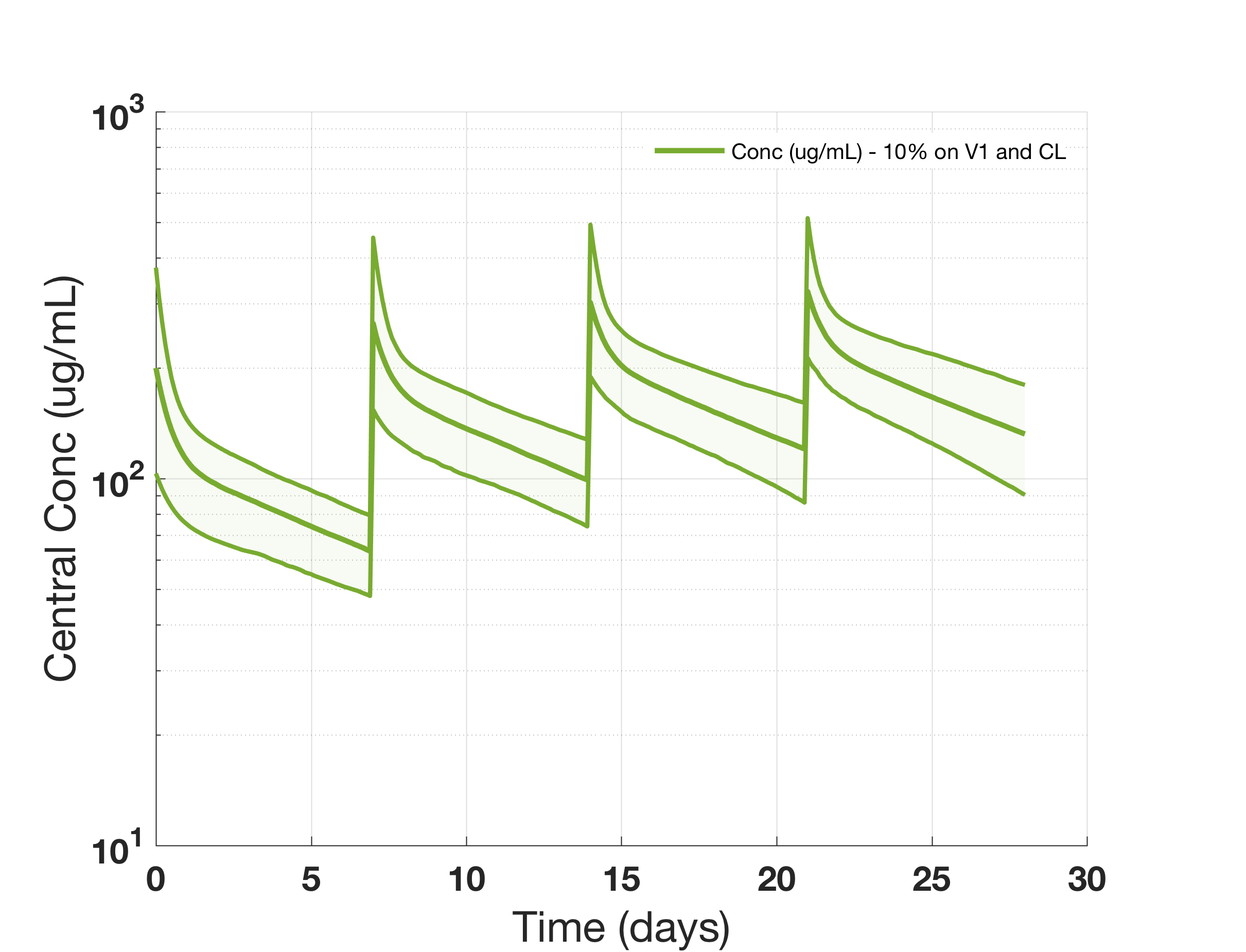

Supplement: Supplementary file 2 — Electronic supplementary material 2 (ZIP 7898 kb) [file 10928_2017_9562_MOESM2_ESM.zip › Supplementary Material/1) Case Study 1/casestudy1_pop_conc.png]

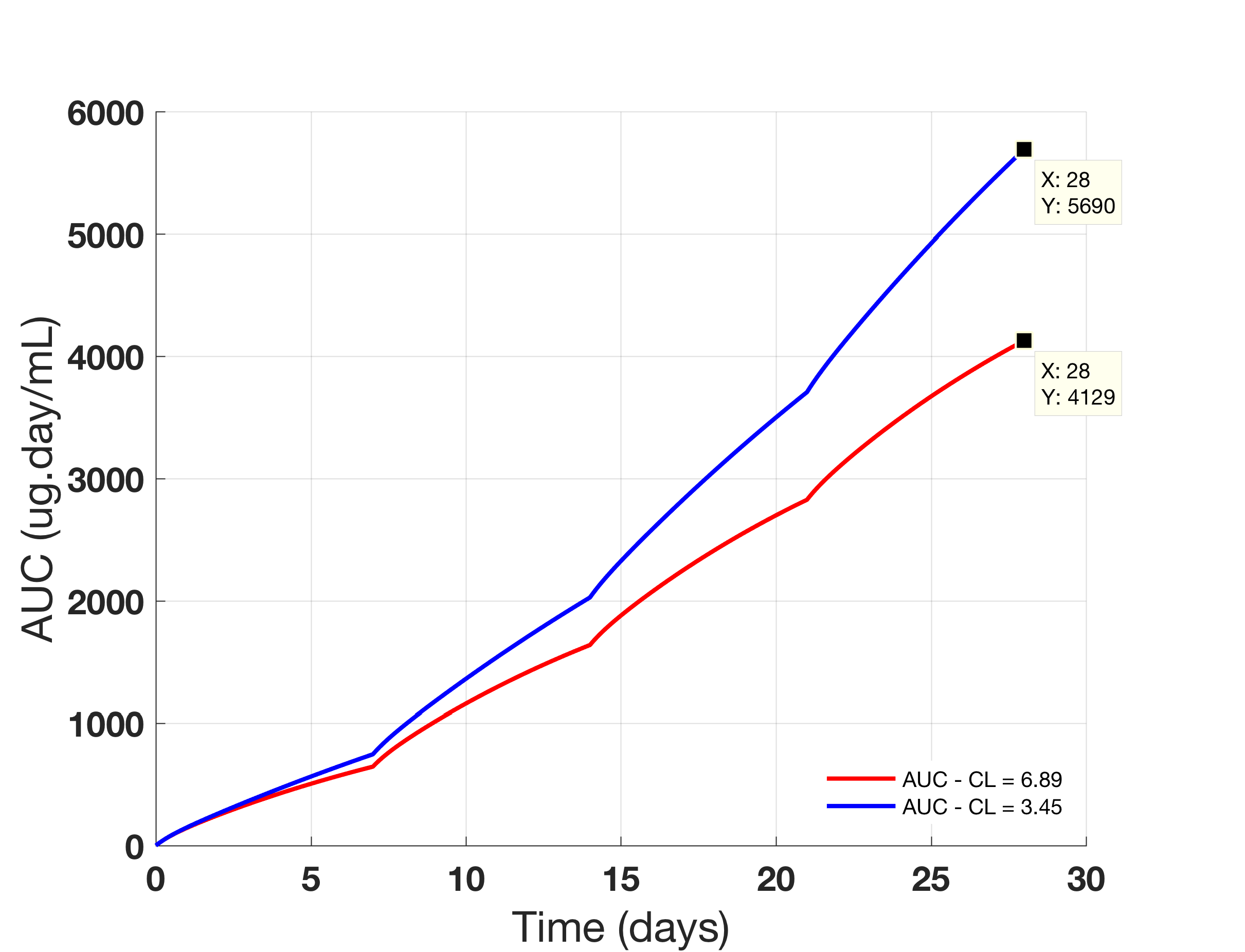

Supplement: Supplementary file 2 — Electronic supplementary material 2 (ZIP 7898 kb) [file 10928_2017_9562_MOESM2_ESM.zip › Supplementary Material/1) Case Study 1/casestudy1_sim_AUC.png]

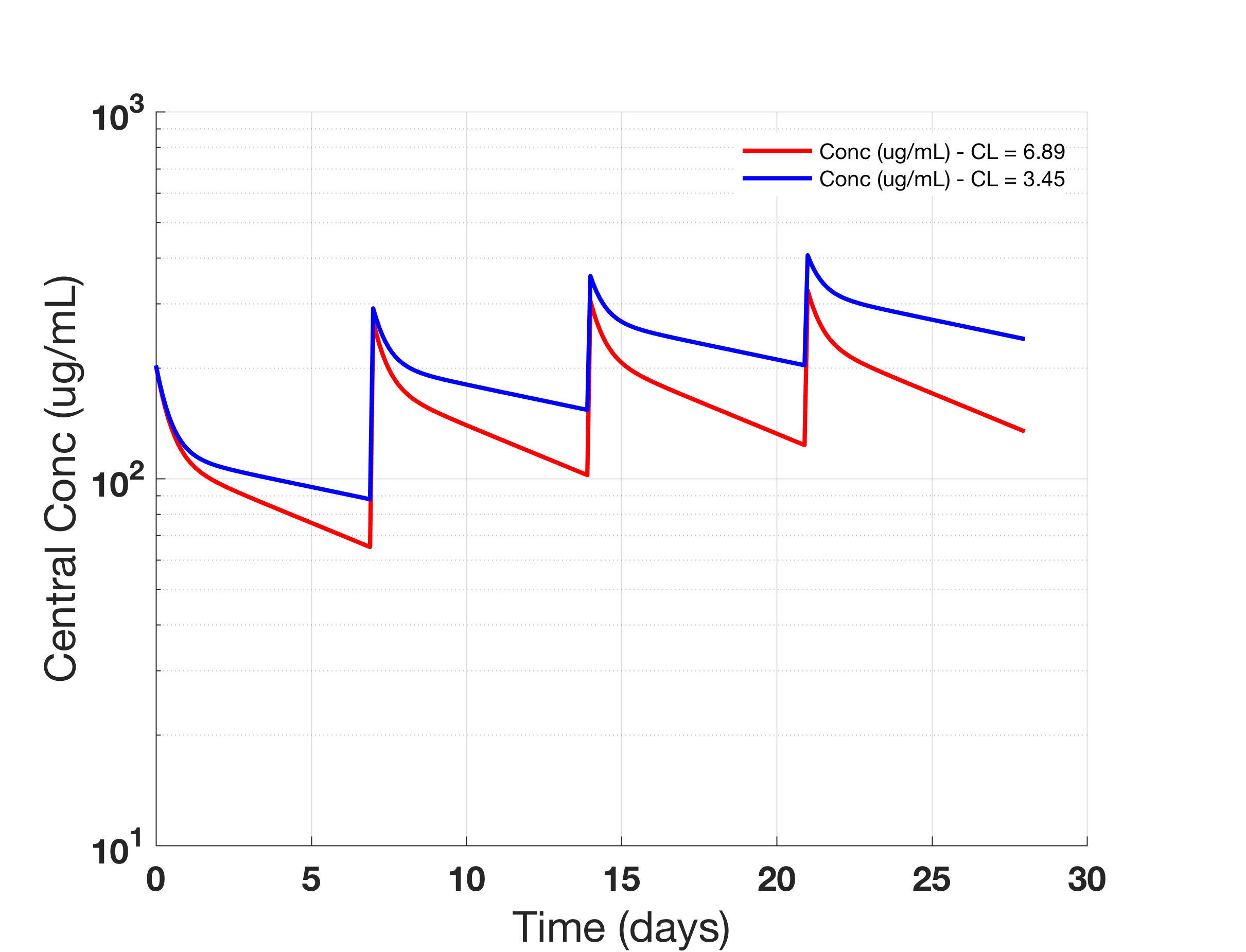

Supplement: Supplementary file 2 — Electronic supplementary material 2 (ZIP 7898 kb) [file 10928_2017_9562_MOESM2_ESM.zip › Supplementary Material/1) Case Study 1/casestudy1_sim_conc.png]

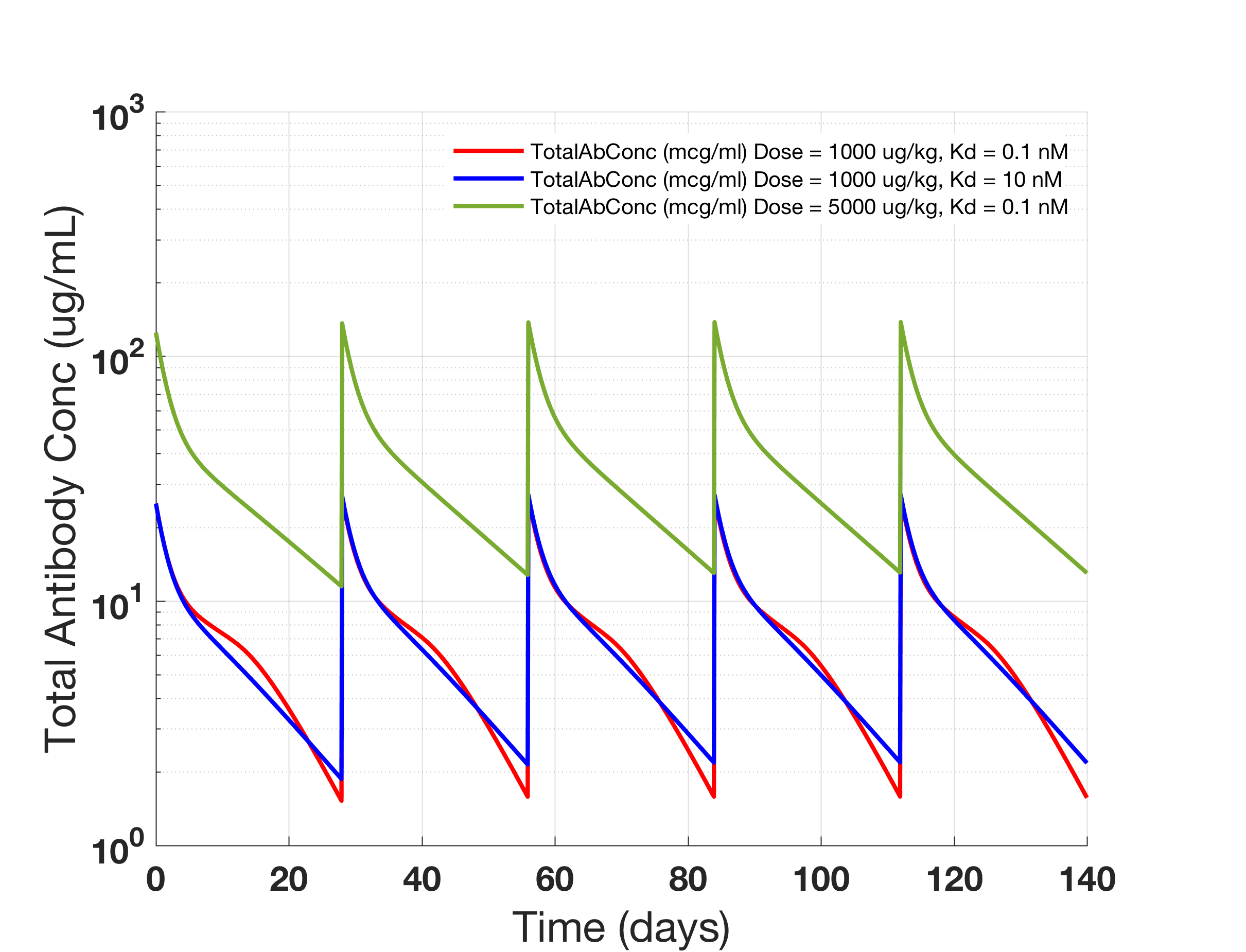

Supplement: Supplementary file 2 — Electronic supplementary material 2 (ZIP 7898 kb) [file 10928_2017_9562_MOESM2_ESM.zip › Supplementary Material/2) Case Study 2/casestudy2_fig_conc.png]

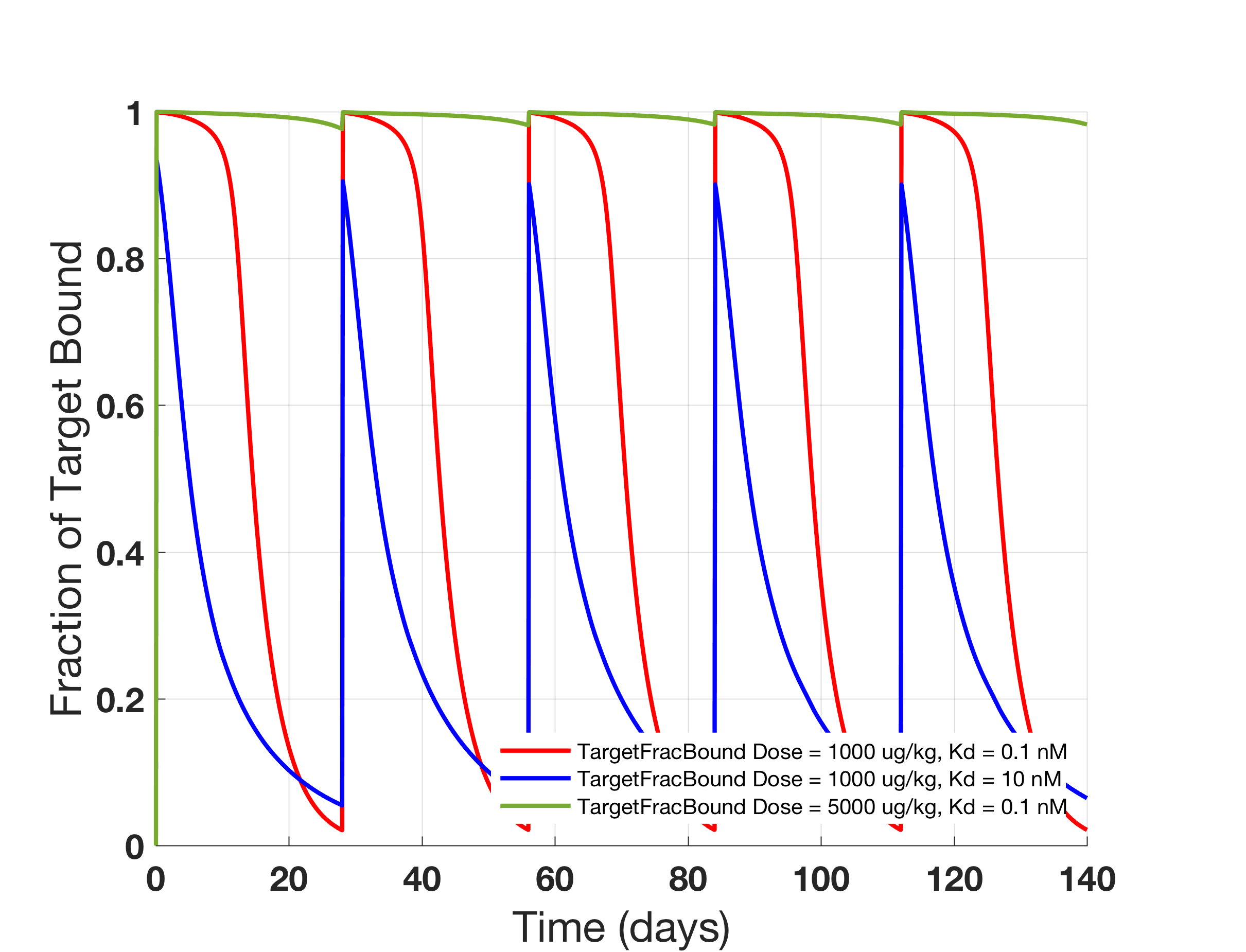

Supplement: Supplementary file 2 — Electronic supplementary material 2 (ZIP 7898 kb) [file 10928_2017_9562_MOESM2_ESM.zip › Supplementary Material/2) Case Study 2/casestudy2_fig_fracbound.png]

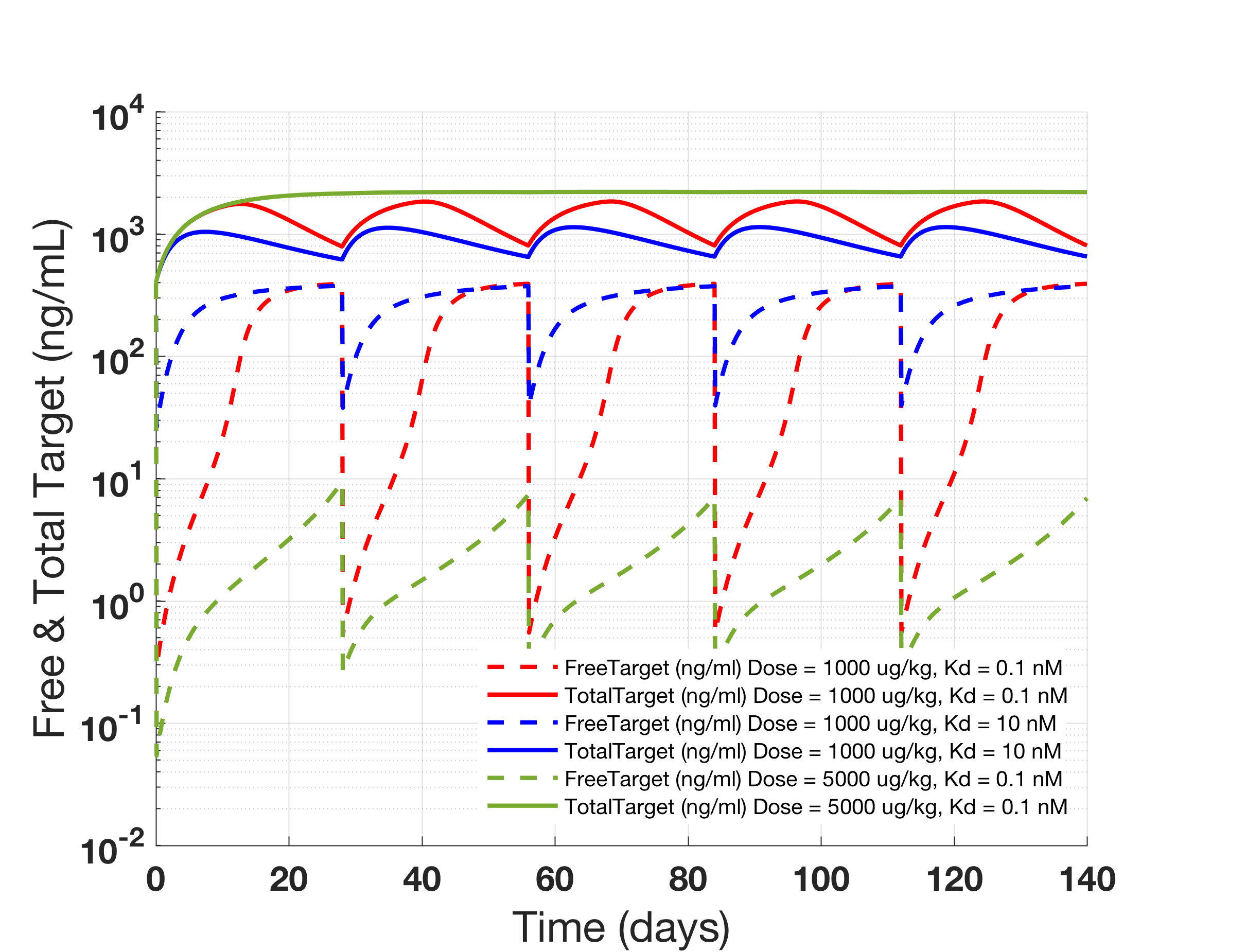

Supplement: Supplementary file 2 — Electronic supplementary material 2 (ZIP 7898 kb) [file 10928_2017_9562_MOESM2_ESM.zip › Supplementary Material/2) Case Study 2/casestudy2_fig_target.png]

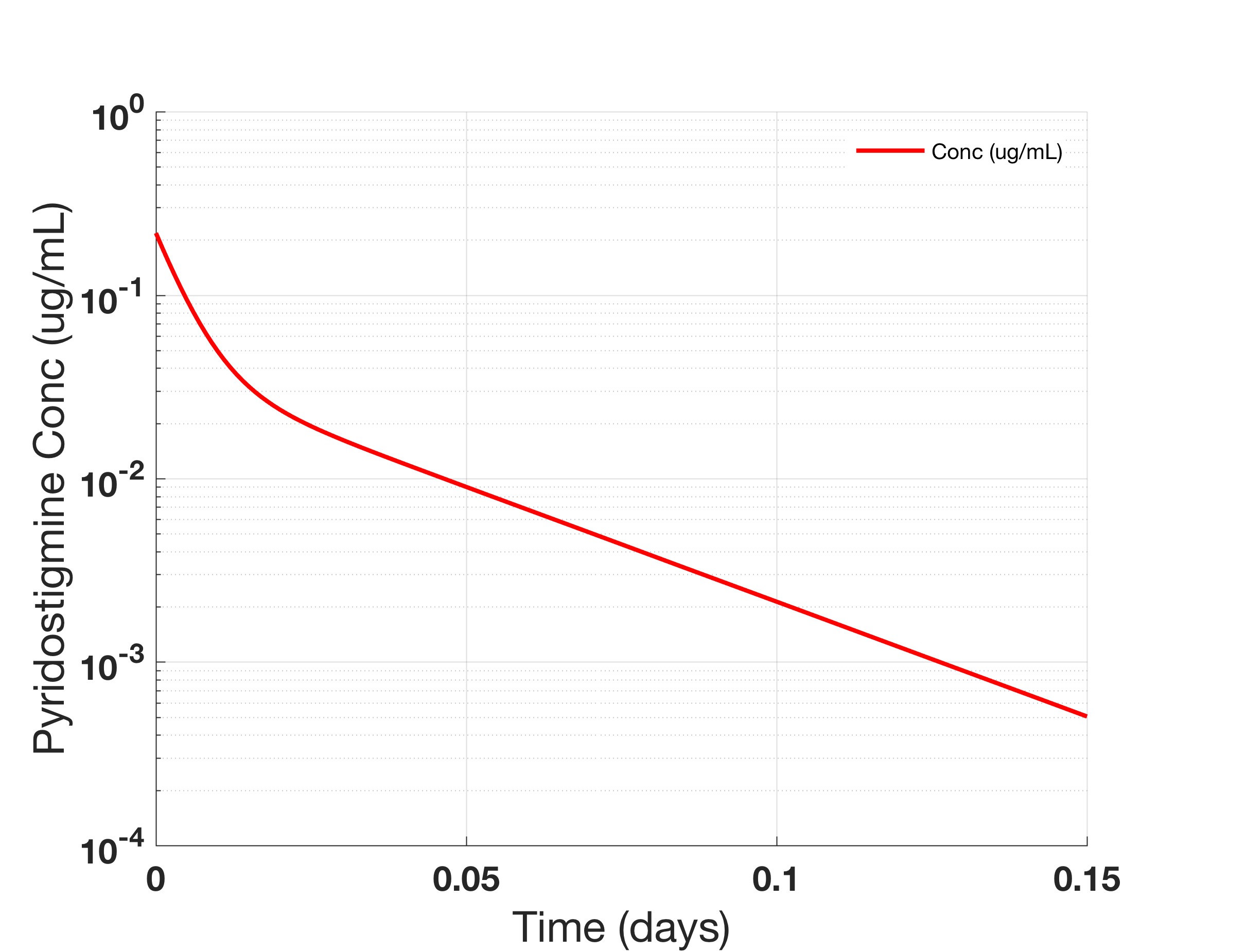

Supplement: Supplementary file 2 — Electronic supplementary material 2 (ZIP 7898 kb) [file 10928_2017_9562_MOESM2_ESM.zip › Supplementary Material/3) Case Study 3/casestudy3_Example1_Fig_1.png]

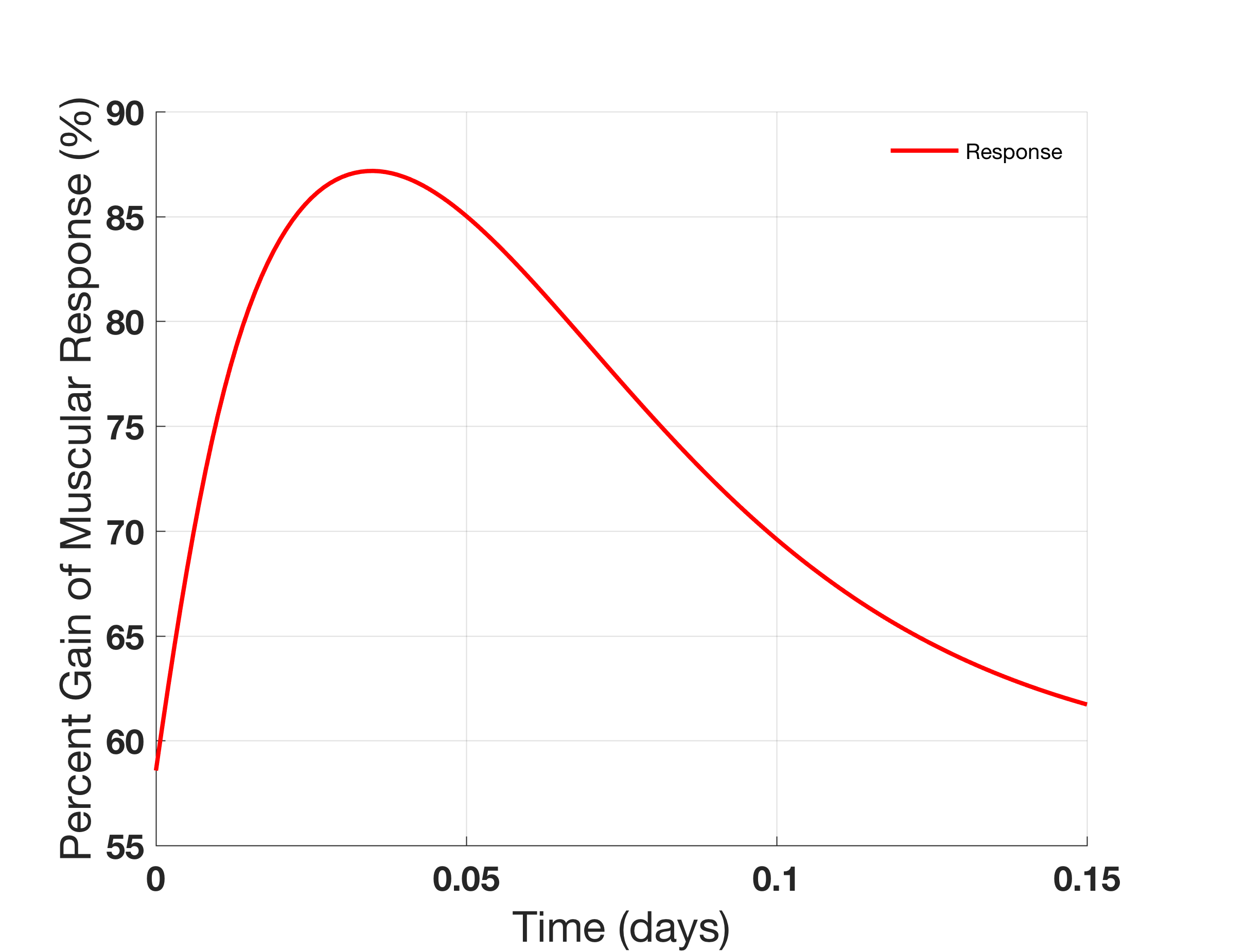

Supplement: Supplementary file 2 — Electronic supplementary material 2 (ZIP 7898 kb) [file 10928_2017_9562_MOESM2_ESM.zip › Supplementary Material/3) Case Study 3/casestudy3_Example1_Fig_2.png]

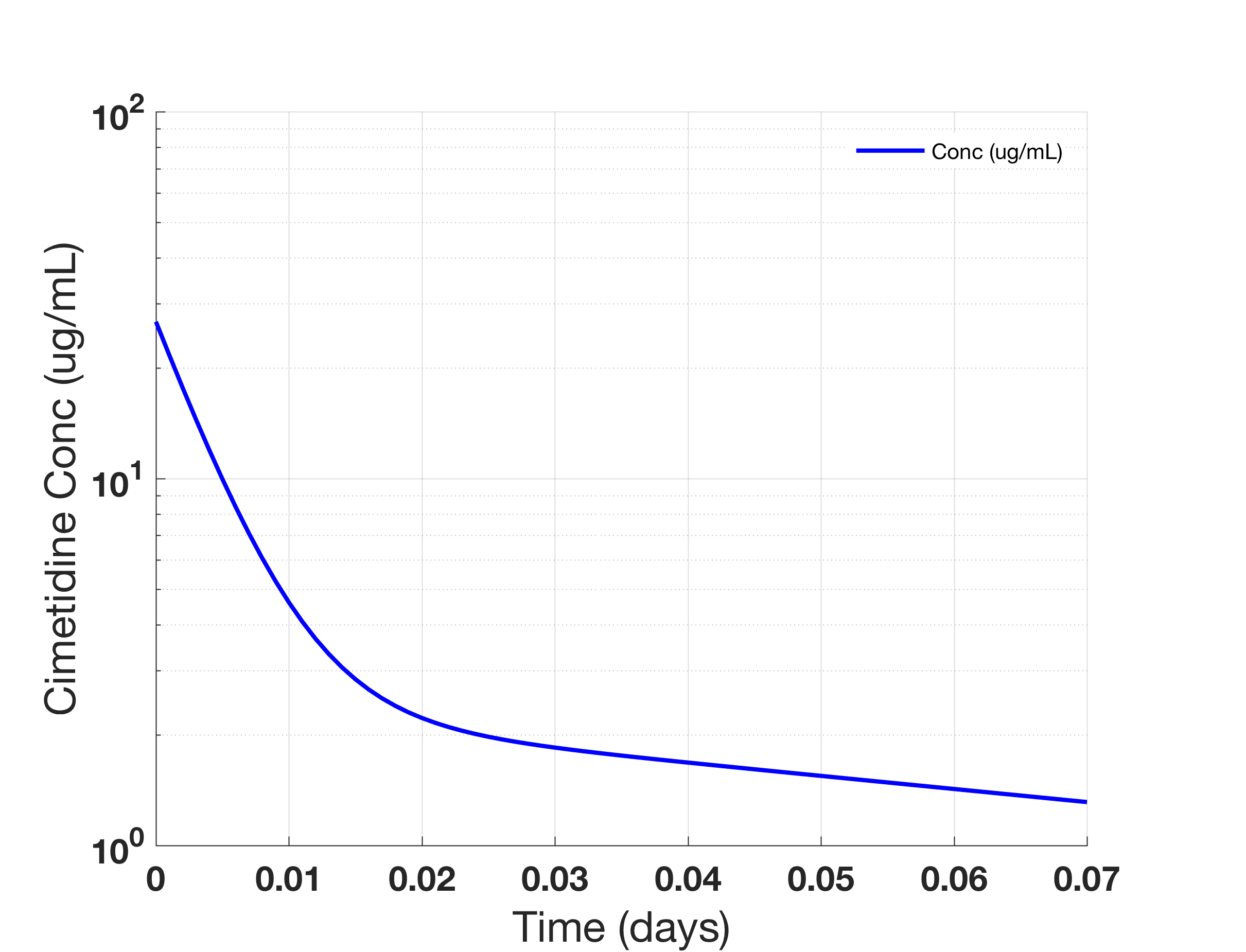

Supplement: Supplementary file 2 — Electronic supplementary material 2 (ZIP 7898 kb) [file 10928_2017_9562_MOESM2_ESM.zip › Supplementary Material/3) Case Study 3/casestudy3_Example2_Fig_1.png]

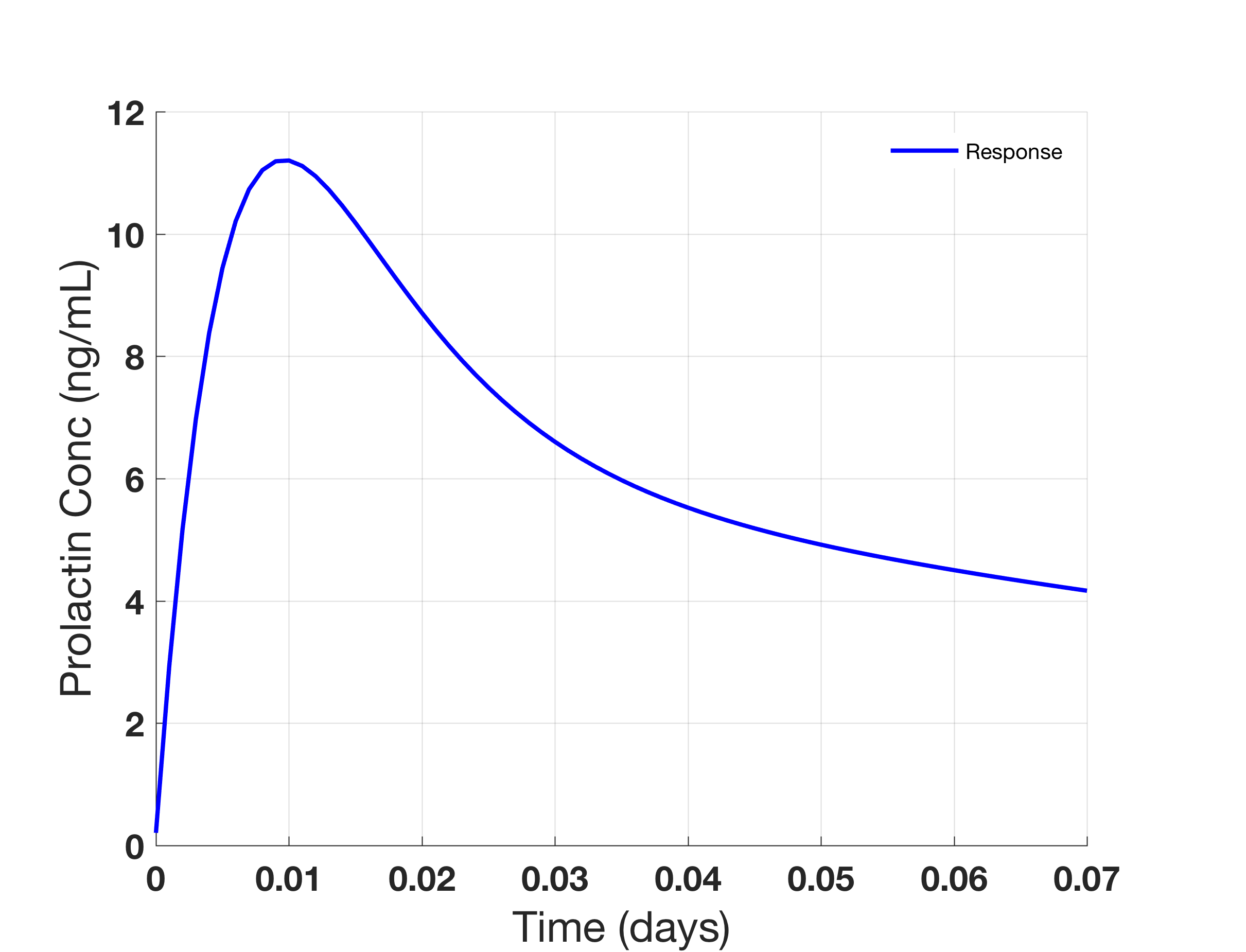

Supplement: Supplementary file 2 — Electronic supplementary material 2 (ZIP 7898 kb) [file 10928_2017_9562_MOESM2_ESM.zip › Supplementary Material/3) Case Study 3/casestudy3_Example2_Fig_2.png]

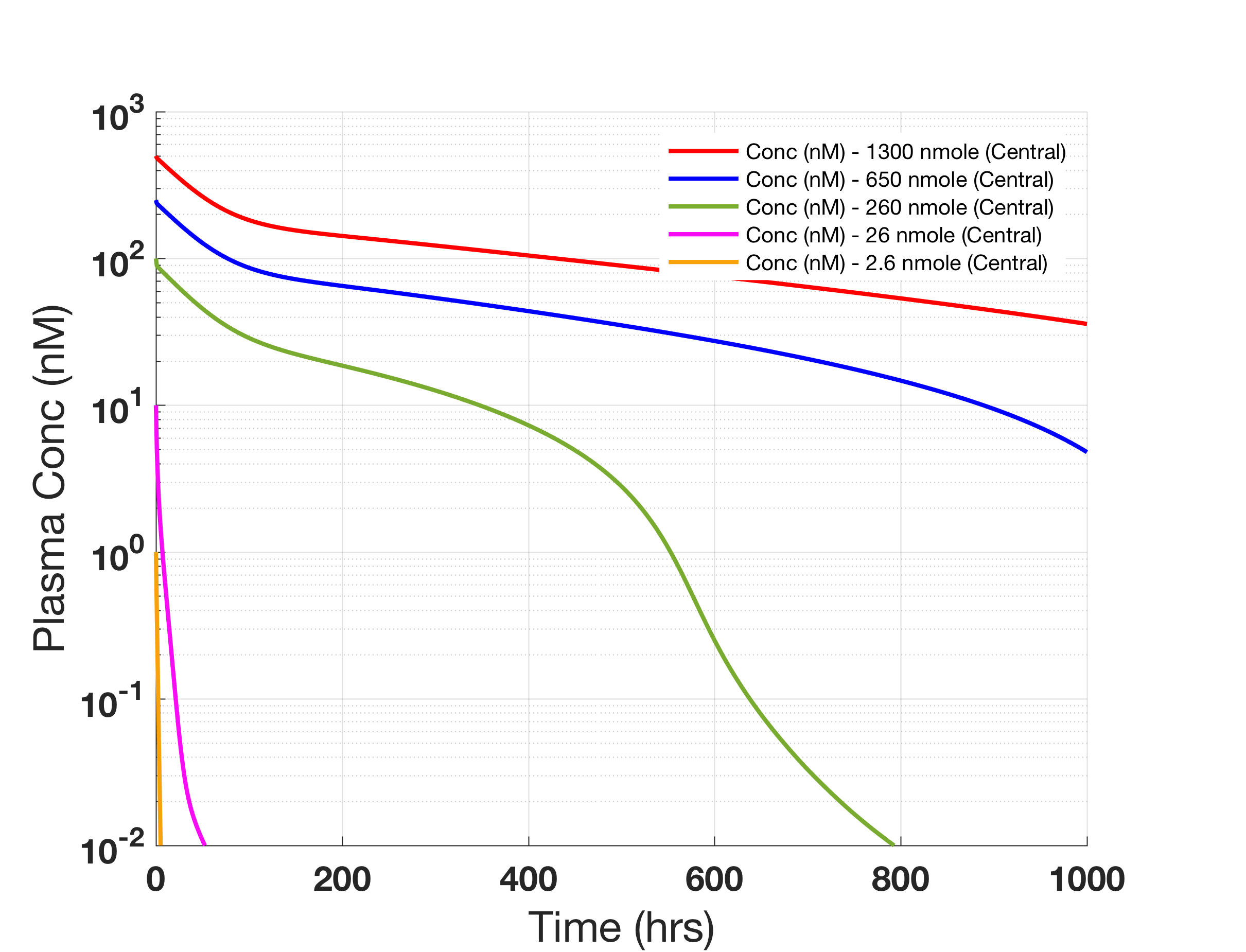

Supplement: Supplementary file 2 — Electronic supplementary material 2 (ZIP 7898 kb) [file 10928_2017_9562_MOESM2_ESM.zip › Supplementary Material/4) Case Study 4/casestudy4_Fig_Central.png]

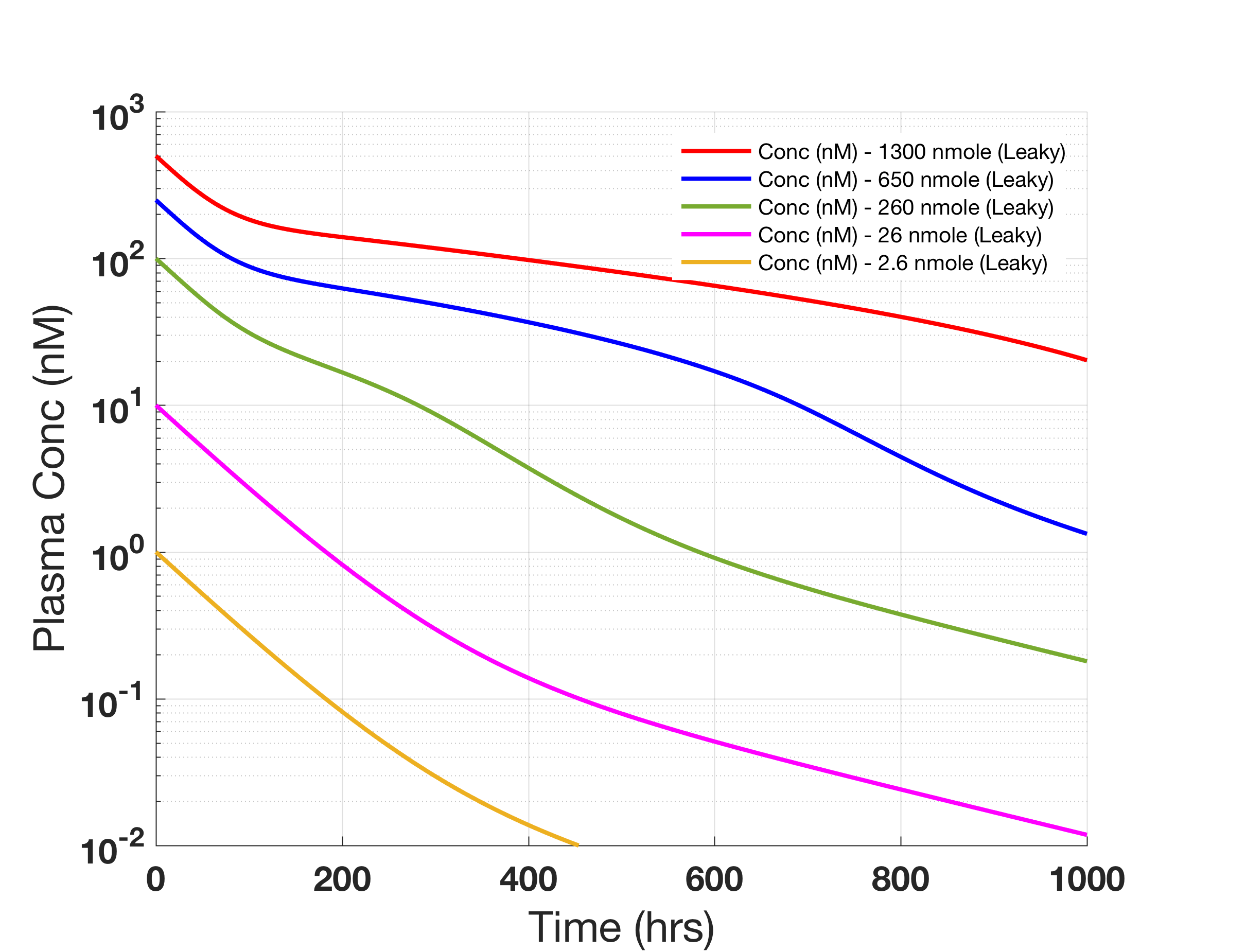

Supplement: Supplementary file 2 — Electronic supplementary material 2 (ZIP 7898 kb) [file 10928_2017_9562_MOESM2_ESM.zip › Supplementary Material/4) Case Study 4/casestudy4_Fig_Leaky.png]

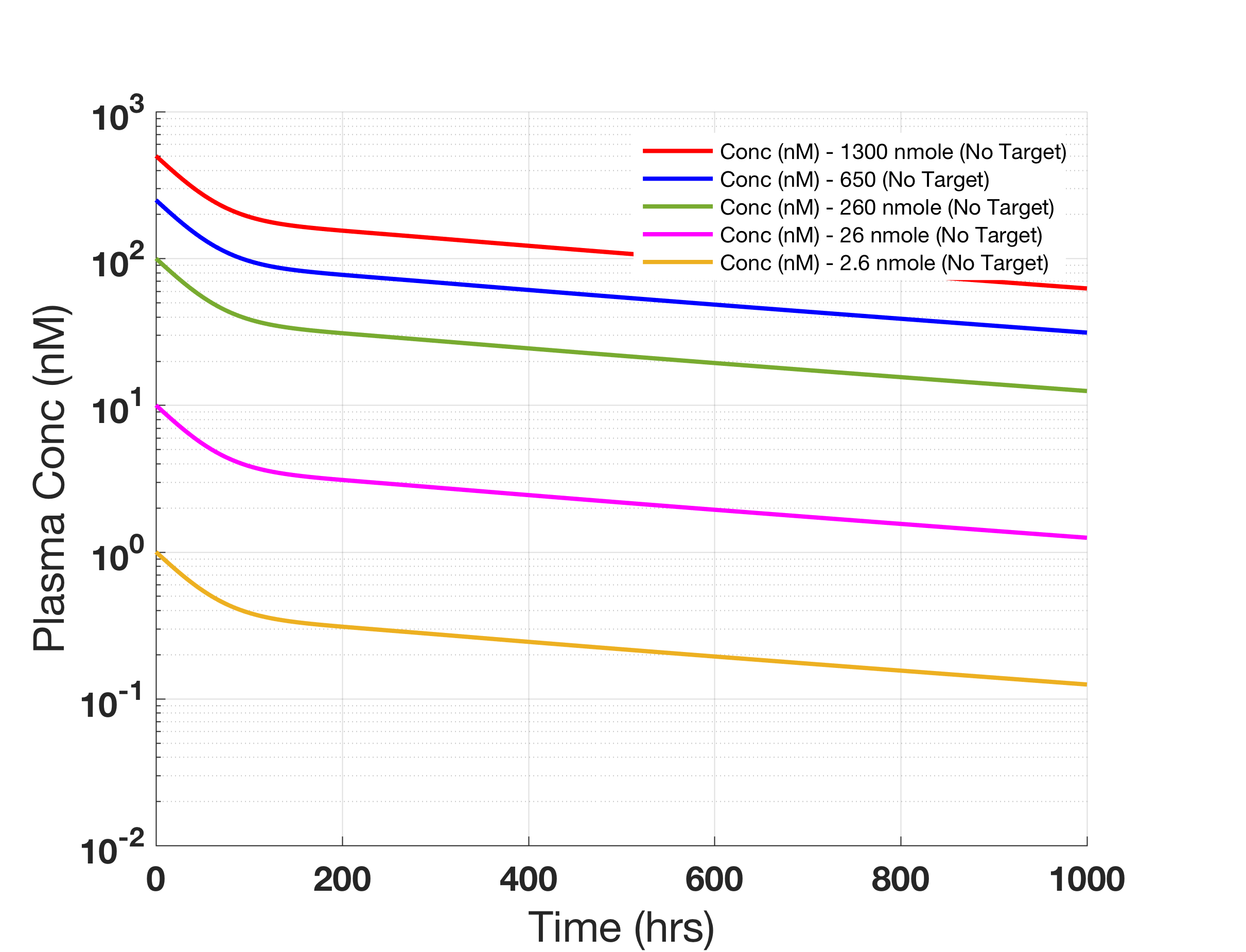

Supplement: Supplementary file 2 — Electronic supplementary material 2 (ZIP 7898 kb) [file 10928_2017_9562_MOESM2_ESM.zip › Supplementary Material/4) Case Study 4/casestudy4_Fig_NoTarget.png]

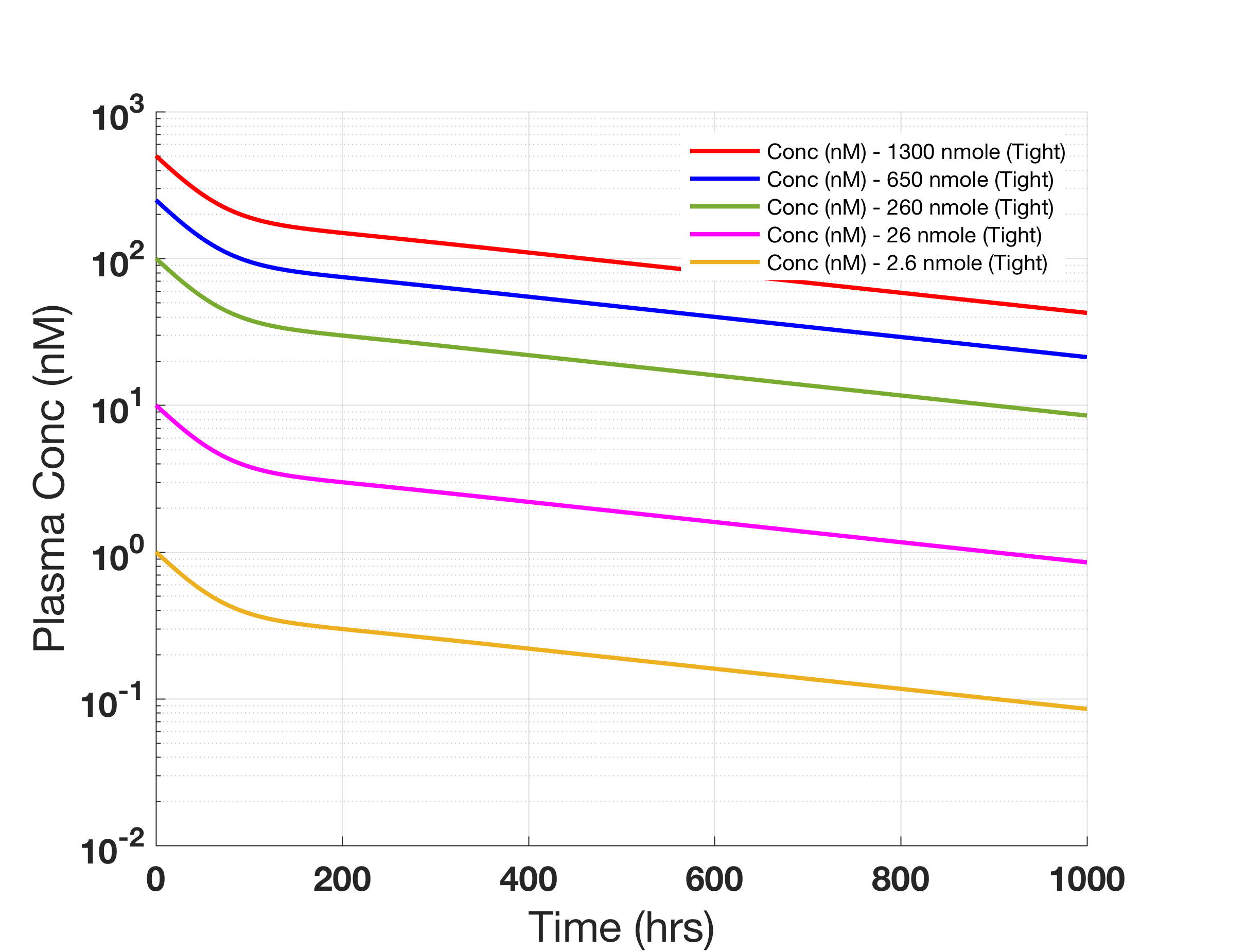

Supplement: Supplementary file 2 — Electronic supplementary material 2 (ZIP 7898 kb) [file 10928_2017_9562_MOESM2_ESM.zip › Supplementary Material/4) Case Study 4/casestudy4_Fig_Tight.png]
